# Cobalt-Catalyzed Asymmetric Hydrogenation of Enamides: Insights into Mechanisms and Solvent Effects

**DOI:** 10.1021/acs.organomet.2c00180

**Published:** 2022-07-25

**Authors:** Ljiljana Pavlovic, Lauren N. Mendelsohn, Hongyu Zhong, Paul J. Chirik, Kathrin H. Hopmann

**Affiliations:** † Department of Chemistry, UiT - The Arctic University of Norway, N-9037 Tromsø, Norway; ‡ Department of Chemistry, Princeton University, Princeton, New Jersey 08544, United States

## Abstract

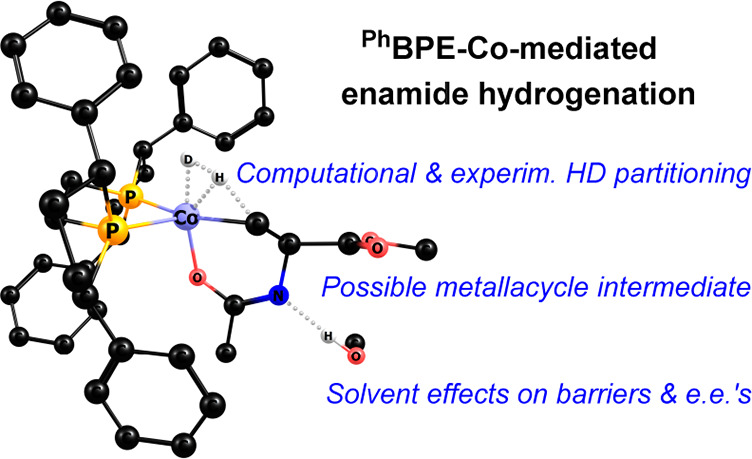

The mechanistic details of the (^Ph^BPE)Co-catalyzed asymmetric hydrogenation of enamides are investigated using computational and experimental approaches. Four mechanistic possibilities are compared: a direct Co(0)/Co(II) redox path, a metathesis pathway, a nonredox Co(II) mechanism featuring an aza-metallacycle, and a possible enamide–imine tautomerization pathway. The results indicate that the operative mechanism may depend on the type of enamide. Explicit solvent is found to be crucial for the stabilization of transition states and for a proper estimation of the enantiomeric excess. The combined results highlight the complexity of base-metal-catalyzed hydrogenations but do also provide guiding principles for a mechanistic understanding of these systems, where protic substrates can be expected to open up nonredox hydrogenation pathways.

## Introduction

In homogeneous hydrogenation catalysis, increasing attention is being devoted toward the use of earth-abundant 3d metals instead of their precious counterparts.^1,2^ The motivation to use non-noble metals lies in their abundance, lower toxicity, and reasonable cost.^3^ However, the 3d transition metals may have properties different from those of precious-metal systems. Whereas the latter typically react via two-electron processes, including elementary steps such as oxidative addition and reductive elimination,^4−7^ 3d metals have more accessible oxidation states, allowing for additional one-electron processes.^8,9^ They may also simultaneously display redox and nonredox pathways,^10,11^ making the search for their reaction mechanisms more unpredictable and challenging.

A number of experimental^12−23^ and computational hydrogenation studies^10,24−29^ have been reported with 3d transition metal catalysts; however, the use of such systems in enantioselective hydrogenation remains less explored.^30−39^ Examples include the Fe-based asymmetric hydrogenation of ketones^35,39^ and imines^38^ and Co-based protocols for the asymmetric hydrogenation of alkenes,^2,30,31,34,40^ carboxylic acids,^41−43^ and enynes.^44^

**Scheme 1:**
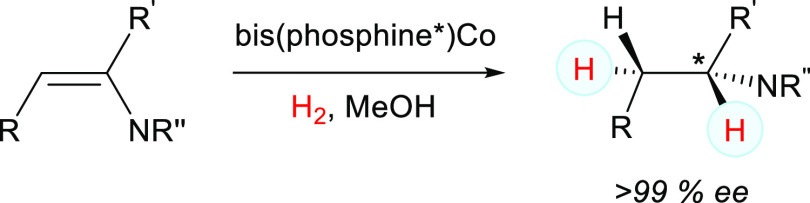
Enamide Hydrogenation with Bis(phosphine)-Co^34^

Recently, we reported the Co-catalyzed asymmetric hydrogenation of enamides^34^ and showed that chiral bidentate phosphine ligands, known to give high enantiomeric excesses in Rh- and Ru-based hydrogenations,^45,46^ also provide excellent results with cobalt (Scheme 1). Interestingly, the highest yields and enantiomeric purities were obtained with protic solvents such as methanol and ethanol.^34^ However, the mechanistic details of the bis(phosphine)-Co-catalyzed enamide reduction and the role of the solvent are not known.

**Scheme 2:**
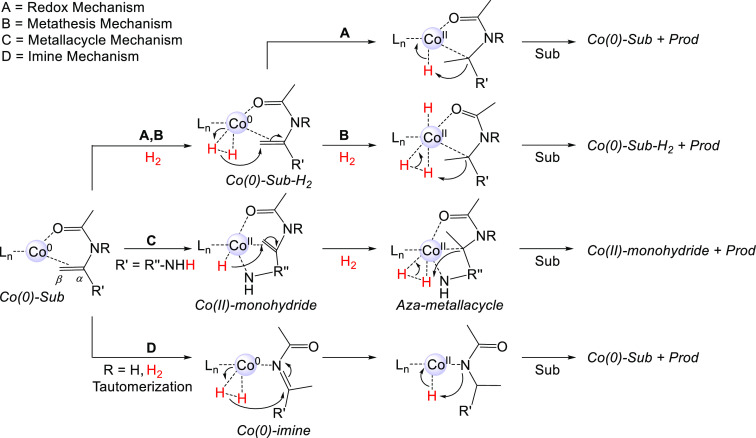
Possible Mechanisms for the Co-Catalyzed Hydrogenation of Enamides For a discussion and references, see the main text. Mechanisms **A**–**C** are shown with initial hydride transfer to Cβ, but Cα is also possible. For mechanism **D**, initial transfer to N is also possible.

We have previously shown that achiral bis(phosphine) cobalt complexes may access different mechanisms for the hydrogenation of alkenes.^10^ Whereas nonfunctionalized alkenes appear to be hydrogenated through a redox pathway cycling between Co(0) and Co(II) states, hydroxylated alkenes prefer a nonredox Co(II) metallacycle pathway. The OH group in the active substrates was placed a minimum of one atom from the double bond, with the computational results indicating that its primary function is to form a stable metallacyclic intermediate.^10^ From these previous results, it is not possible to predict which mechanism is preferred in the Co-mediated hydrogenation of enamides, which have a functional group (NR) directly at the double bond. If we assume a resting state of Co(0)-enamide,^47^ at least four mechanistic possibilities can be envisioned (**A–D**, Scheme 2). The classic Co(0)–Co(II) redox mechanism **A** has been proposed for bis(phosphine)-Co-catalyzed hydrogenation of alkenes and nitriles.^10,23,30,48^ Mechanism **B** is a σ-bond metathesis pathway related to proposals for alkene hydrogenation with Co(I)-diiminopyridine complexes.^25,33^ Mechanism **C** was proposed by us for the bis(phosphine)-Co-catalyzed hydrogenation of hydroxylated alkenes.^10^ Due to the possibility that enamides may tautomerize to imines, additional mechanistic possibilities arise. Mechanism **D** is related to the mechanisms studied for Ir-catalyzed imine hydrogenation^49,50^ and was also recently considered in Co-mediated imine reduction.^51^

Here, the possible mechanistic pathways of (^Ph^BPE)Co-catalyzed enamide hydrogenation were addressed using experimental and computational approaches, with the aim of establishing the preferred mechanistic routes and obtaining a better understanding of the potential role of the protic solvent. We note that the related (^iPr^DuPhos)Co complex shows a somewhat different mechanistic behavior, which will be reported elsewhere.^52^

## Methods

### Experimental Details

All air- and moisture-sensitive manipulations were carried out using vacuum-line, Schlenk, and cannula techniques or in an MBraun inert-atmosphere (nitrogen) drybox unless otherwise noted. All glassware was stored in a preheated oven prior to use. The solvents used for air- and moisture-sensitive manipulations were dried and deoxygenated using literature procedures.^53^^1^H NMR spectra were recorded on an I400 Varian Inova spectrometer operating at 400 MHz. ^13^C{^1^H} NMR spectra were recorded on a Bruker A500 spectrometer operating at 126 MHz. ^31^P{^1^H} NMR spectra were recorded on an I400 Varian Inova spectrometer operating at 162 MHz. All ^1^H chemical shifts are reported in ppm relative to SiMe_4_ using the ^1^H (CDCl_3_: 7.26 ppm) chemical shifts of the solvent as a standard. Gas chromatography for the alkane products was performed on a Shimadzu GC-2010 gas chromatograph. GC analyses were performed using a Restek 15 m × 0.25 mm RTX-5 5% diphenyl/95% dimethyl polysiloxane column with a film thickness of 0.25 μm. *dehydro-*Levetiracetam was purchased from Sundia Meditech (Shanghai, China) and used as is. Methyl 2-acetamidoacrylate was purchased from Sigma-Aldrich and purified by Et_2_O filtration through silica. Both chemicals were dried on a high-vacuum line prior to use.

### Hydrogenation of MAA

In a nitrogen-filled glovebox, a thick-walled glass vessel was charged with MAA (0.014 g, 0.10 mmol), (*S*,*S*)-(^Ph^BPE)CoCl_2_ (0.002 g, 0.003 mmol, 3 mol %), Zn (0.007 g, 0.10 mmol, 100 mol %), MeOH (1.5 mL), and a stir bar. The vessel was sealed and removed from the glovebox. On a high-vacuum line, the solution was frozen and the headspace removed under vacuum. The vessel was backfilled with 4 atm of H_2_. The solution was sealed, thawed, and stirred at 50 °C in an oil bath for 18 h. Following this time, the reaction was air-quenched and the solvent evaporated. The crude mixture was taken up in CDCl_3_ and filtered through an alumina plug. The resulting sample was analyzed by ^1^H NMR and chiral GC.

### HD Experiments

In a nitrogen-filled glovebox, a 4 mL vial was charged with a MeOH solution (the total volume for each trial was equal to 2 mL) with MAA or DHL (0.20 mmol) and (*R,R*)-(^Ph^BPE)Co(COD) or (*R,R*)-(^Ph^BPE)CoCl_2_ (0.04 mmol, 2 mol %; Zn (20 mol %) was used with the dihalide) and a stir bar. The vial was then placed into a high-pressure reactor, sealed, and removed from the glovebox. The reactor was backfilled with 60 psi of HD and the mixture allowed to react for 5 days. At this point the reaction was air-quenched and the volatiles were evaporated under air. The residue was then taken up with EtOAc and filtered through an alumina plug. The solvent was removed, and the residue was taken up in CHCl_3_ or CDCl_3_. Deuterium incorporations were determined using ^1^H, ^2^H, and quantitative ^13^C NMR spectroscopy.

### H_2_/D_2_ Scrambling

In a nitrogen-filled glovebox, a J. Young NMR tube was charged with a C_6_D_6_ (0.5 mL) solution of (*R,R*)-(^Ph^BPE)Co(COD) (0.010 g, 0.015 mmol) (tube 1). A second J. Young NMR tube was sealed but left empty (tube 2). The tubes were removed and taken to a high-vacuum line. The solution in tube 1 was frozen, and the headspace was removed under vacuum. The tube was backfilled with 4 atm of H_2_, and the solution was kept frozen. Tube 2 was similarly evacuated and backfilled with 4 atm of D_2_. The two tubes were subsequently placed on a two-port J-Young tube connector with an isolable headspace on the high-vacuum line, which was evacuated in the middle. The gases of both tubes were allowed to mix for 10 min with the solution still frozen, after which tube 1 was sealed and the contents were thawed and mixed. The contents were analyzed by ^1^H NMR.

### Computational Models

**Figure 1:**
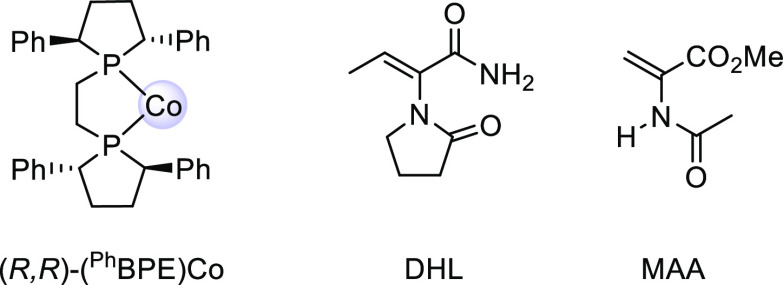
Metal complex and substrates studied computationally (DHL, *dehydro*-levetiracetam; MAA, methyl 2-acetamidoacrylate).

Full molecular systems, consisting of (*R,R*)-(^Ph^BPE)Co and the substrates, were computed (Figure 1), without truncations or symmetry constraints. A low-spin *S* = 1/2 spin state was employed in the computations, as determined experimentally for the (*R,R*)-(^Ph^BPE)Co complex.^34^ A computational evaluation of quartet states confirmed that they are more than 10 kcal/mol higher in energy (Table S4 in the Supporting Information). Zn was not included in the model, as the experimental studies have shown that it is not needed if the hydrogenation sets out from a (^Ph^BPE)Co(0)(COD) species.^34^

### Computational Methods

All geometry optimizations and frequency calculations were performed with the Gaussian09^54^ package, Rev. D01. The DFT hybrid functional B3LYP^55,56^ was employed with the Grimme empirical dispersion correction D3^57^ (results for other DFT functionals are given in Table S3 in the Supporting Information). The IEFPCM model with parameters for methanol was used in order to include solvent effects.^58,59^ For geometry optimizations, basis set BS1 was employed, which consists of 6-311G(d,p)^60^ on all nonmetals, and the LANL2TZ^61^ basis set and pseudopotential on Co. The optimized structures displayed only real vibrational frequencies, with the exception of all transition state structures, which exhibited one imaginary frequency. In order to obtain more accurate energies, single-point calculations were performed with 6-311++G(2df,2pd) on all nonmetals, whereas the basis set and the pseudopotential LANL2TZ were used on Co (BS2). Counterpoise corrections computed at the BS2 level (CP_BS2_) were included in order to correct for the artificial lowering of the electronic energy caused by the borrowing of basis functions, when molecular fragments are joined into one model. The computed free energies in the gas phase (Δ*G*°_1 atm, BS 1_) were converted into the corresponding 1 M standard state energies employing a standard state (SS) conversion term.^62^ Only reactions where the number of moles changes are affected. For the reaction A + B = C at 323.15 K, SS = −2.1 kcal/mol for a 1 M standard state. For explicit solvent, the standard state of the pure solvent was employed (24.7 M for MeOH, derived from the density of 0.792 g/mL), which results in a correction of −4.2 kcal/mol. Temperature corrections were included in all free energies to match the experimental temperature (50 °C). The standard state Gibbs free energies (Δ*G*°_1 M,323 K_) reported in the main text correspond to
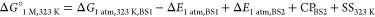
1

Enantiomeric excesses were evaluated from the computed barriers for the rate-limiting steps using the following formula:^63^
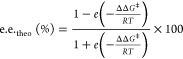
2

For computations on HD systems, the Gibbs free energies with deuterium were obtained by redoing the frequency calculations using freq = (readfc,readisotopes) with the mass of the selected hydrogen being replaced with the mass of deuterium. Isotopic ratios of the products were calculated from the ratio of the computed rates (at 298 K) obtained for initial H transfer versus initial D transfer from HD to the substrate.

## Results and Discussion

We have previously reported that (*R*,*R*)-(^Ph^BPE)Co provides excellent yields and high enantiomeric excesses in the reduction of methyl 2-acetamidoacrylate (MAA) and *dehydro*-levetiracteam (DHL) (Table 1), the hydrogenation of which leads to the chiral antiepileptic drug Keppra.^34^ For DHL, labeling studies with D_2_ supported a mechanistic pathway involving homolytic cleavage of hydrogen,^34^ but no other mechanistic information for (*R*,*R*)-(^Ph^BPE)Co-mediated enamide hydrogenation has been determined.

**Table 1:**
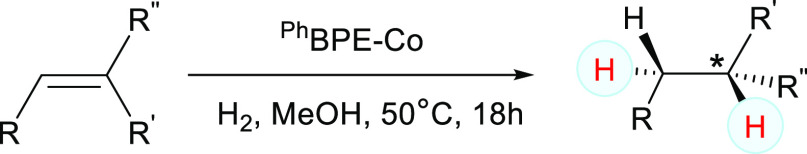

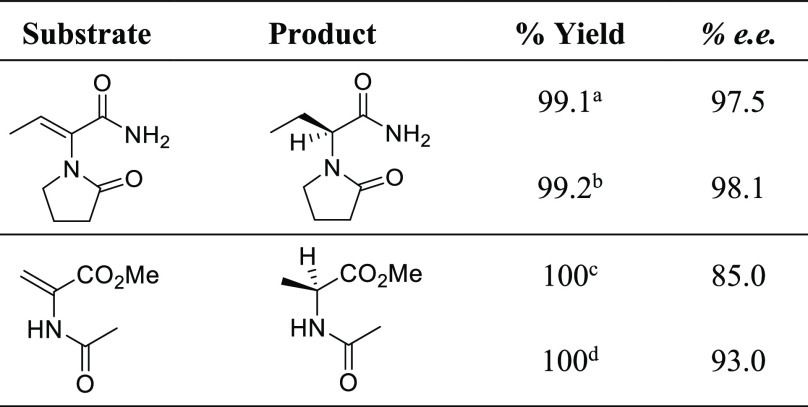
(*R,R*)-(^Ph^BPE)Co-Mediated Enamide Hydrogenation

a Conditions: 0.5 mol % (*R*,*R*)-(^Ph^BPE)Co(COD), 4 atm H_2_. e.e.: 97.5% (*S*).^34^

b Conditions: (*R*,*R*)-(^Ph^BPE)CoCl_2_ formed *in situ* from 10.5 mol % of the ligand, 10 mol % of CoCl_2_, 100 mol % of Zn, e.e.: 98.1% (*S*).^34^

c Conditions: (*R*,*R*)-(^Ph^BPE)CoCl_2_ formed *in situ* from 10.5 mol % of the ligand, 10 mol % of CoCl_2_, 100 mol % of Zn, 500 psi of H_2_. e.e.: 85.0% (*S*).^34^

d Conditions: 3 mol % of (*S*,*S*)-(^Ph^BPE)CoCl_2_, 100 mol % of Zn, 4 atm of H_2_. e.e.: 93.0% (*R*) (Figure S1).

**Figure 2:**
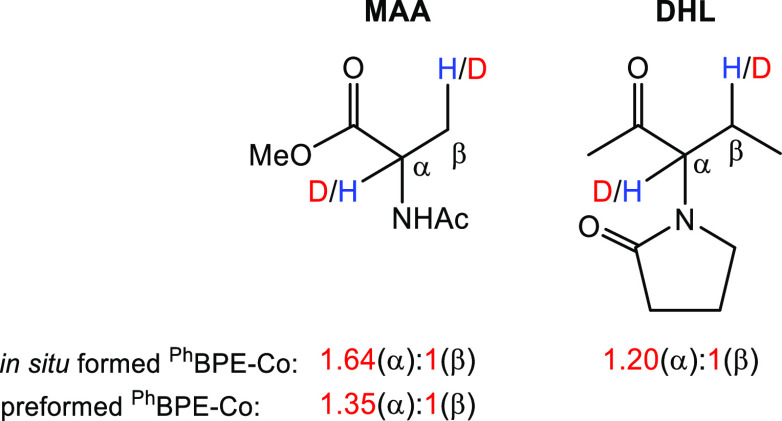
HD partitioning results for DHL and MAA (see Experimental Details).

In order to obtain additional mechanistic information, catalytic reduction of a MeOH solution of DHL or MAA (0.10 M) with HD (60 psi) was performed at room temperature, using (*R*,*R*)-(^Ph^BPE)Co(COD) (2 mol %) and/or (*R*,*R*)-(^Ph^BPE)CoCl_2_ (with *in situ* Zn reduction, 2 mol % cobalt) as the precatalysts (Figure 2, Figures S3–S12 (MAA), and Figures S13–S18 (DHL) in the Supporting Information). ^1^H, ^2^H, and quantitative ^13^C NMR spectroscopy demonstrated preferential deuterium incorporation into the Cα-position of MAA in a 1.35:1 ratio by (*R*,*R*)-(^Ph^BPE)Co(COD), which is comparable to the value found using identical conditions with (*R*,*R*)-(^iPr^DuPhos)Co(COD) as the precatalyst (1.45:1),^52^ as well as that reported with [Rh(DIPHOS)(NBD)][BF_4_] (1.36:1) in MeOH.^64^ (*R*,*R*)-(^Ph^BPE)CoCl_2_ formed *in situ* with Zn reduction also showed preferential deuterium incorporation into the Cα-position, with a 1.64:1 partitioning ratio for MAA and 1.20:1 for DHL. The higher ratio for MAA with the *in situ* formed catalyst may be due to the possibility that the preformed (*R*,*R*)-(^Ph^BPE)Co(COD) is more prone to form hydrides during its activation, which may lead to HD scrambling and formation of HH and DD, which would result in less partitioning. It should be noted that there is no direct comparison for the HD labeling of DHL in the rhodium literature.

For the splitting of HD, it can be expected that the first step will have a kinetic preference for transfer of H to the double bond (and formation of Co-D), with the transfer of D being more likely in the second hydrogen transfer step (this is also supported by computations, *vide infra*). The HD labeling results thus indicate a preference for a mechanism where the first step involves hydrogen transfer to the Cβ atom of MAA or DHL, such that deuterium primarily ends up on Cα. While this does not help to discriminate among mechanisms **A**–**C** (Scheme 2), it can be noted that mechanism **D** is less supported by these results, as the first hydrogen transfer from HD/H_2_ is to the α-carbon (see also Figure S30).

Interestingly, the ^13^C NMR spectra of both preformed and *in situ* MAA reactions demonstrated the formation of both HD-containing products, as well as HH and DD products (Figures S3–S12 in the Supporting Information), although the *in situ* reduction method appears to generate a smaller quantity of HH and DD products. For the classical redox pathway **A** (Scheme 2), the use of HD should give products containing one H and one D but should never have products with two H or two D. If either pathway **B** or **C** is operative, all possible HD, DH, HH, and DD products should be observed (as the proton and hydride transfer to the substrate occur from different molecules of hydrogen gas; Scheme 2). While the formation of all four types of products for MAA thus appears to be more in line with mechanism **B** or **C**, it is important to note that if a background scrambling reaction between the catalyst and HD to form H_2_ and D_2_ takes place, it may complicate the results, as has been shown for the related ^iPr^DuPhos catalyst.^52^ Indeed, exposure of a mixture of H_2_ and D_2_ gases to (*R,R*)-(^Ph^BPE)Co(COD) shows the formation of HD by ^1^H NMR within 20 min, supporting that scrambling does occur. Therefore, the labeled products do not provide conclusive evidence about the preferred mechanism. On the other hand, HD labeling of DHL appeared to give no HH and DD products (Figures S13−S18 in the Supporting Information), more supportive of mechanism **A** than either mechanism **B** or **C**.

In order to obtain more mechanistic insights into the enantioselective enamide hydrogenation (Table 1), detailed computational studies were performed, employing DFT methods (B3LYP-D3[IEFPCM]) and full molecular systems (Figure 1). Schematic drawings and energies for all studied pathways can be found in the Supporting Information. Initially, DHL was evaluated, which in addition to the enamide functional group also possesses an ionizable primary amide, making mechanisms **A**–**C** possible options (Scheme 2). Tautomerization of DHL to an imine is not possible, excluding mechanism **D**.

**Scheme 3:**
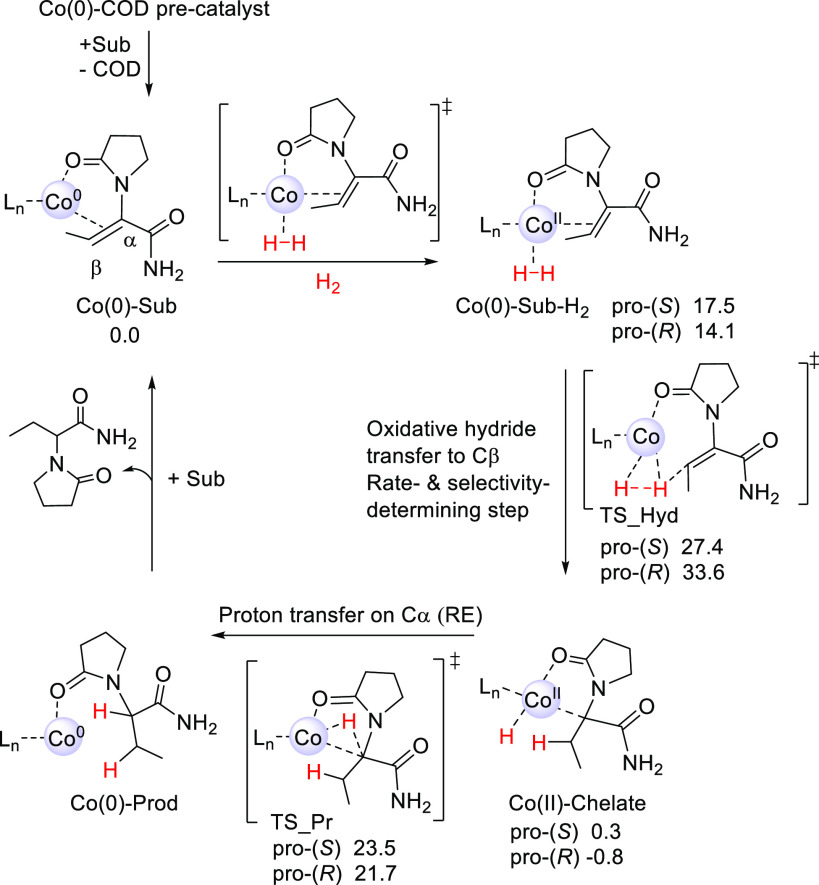
Redox Mechanism A for (*R,R*)-(^Ph^BPE)Co-Catalyzed Hydrogenation of DHL Free energies are relative to Co(0)-Sub (kcal/mol, 323 K, B3LYP-D3/BS2[IEFPCM]//B3LYP-D3/BS1[IEFPCM]).

Hydrogenation of DHL via the redox-type mechanism **A** sets out from a substrate-coordinated species, where the enamide coordinates to cobalt through both the double bond and the oxygen atom of the amide motif (Scheme 3). A similar coordination mode has been observed in the X-ray structure of a cationic [(*R,R*)-(^iPr^DuPhos)Co(MAA)][BAr^F^_4_] (BAr^F^_4_ = tetrakis-3,5-bis(trifluoromethyl)phenyl borate) complex.^2^ Our computations show a very high dissociation energy of almost 50 kcal/mol for breaking the Co-DHL interaction (Figure S20 in the Supporting Information), indicating that the enamide–cobalt bond is strong. It is thus unlikely that cobalt will be uncoordinated when H_2_ binds, as has been proposed in other studies on Co-catalyzed alkene or imine hydrogenation, via a redox mechanism.^2,48^ We further note that a Co(II)-dihydride species is 18.0 kcal/mol above the Co(0)-Sub complex, making the formation of the former unlikely in the presence of enamide.

Coordination of H_2_ to the enamide-coordinated complex leads to the formation of a Co(0)-Sub-H_2_ species, where H_2_ prefers to form a σ-bonded complex and is not oxidatively added to Co, as has also been shown previously for bis(phosphine)-Co-mediated alkene hydrogenation.^10^ In the following step, an oxidative hydride transfer to the β-atom (TS_Hyd) gives an alkyl intermediate, with a computed barrier of 27.4 kcal/mol for the *pro*-(*S*)-coordinated substrate (Scheme 3). TS_Hyd is the rate- and selectivity-determining step of mechanism **A**,^65^ with the overall barrier being considered feasible at the experimental temperature of 323 K.^66^ At the formed intermediate, the substrate behaves as a chelate and interacts with cobalt through the formally anionic carbon and the amide oxygen. Finally, reductive elimination liberates the product and regenerates the Co(0) species (Scheme 3).

Mechanism **B** sets out similar to mechanism **A** with a hydride transfer to the substrate (Scheme 2 and Figure S22 in the Supporting Information). However, after this step, an additional H_2_ molecule binds, which transfers a proton to the substrate. This σ-bond metathesis pathway has a computed barrier of 37.7 kcal/mol, making it nonfeasible.

**Figure 3:**
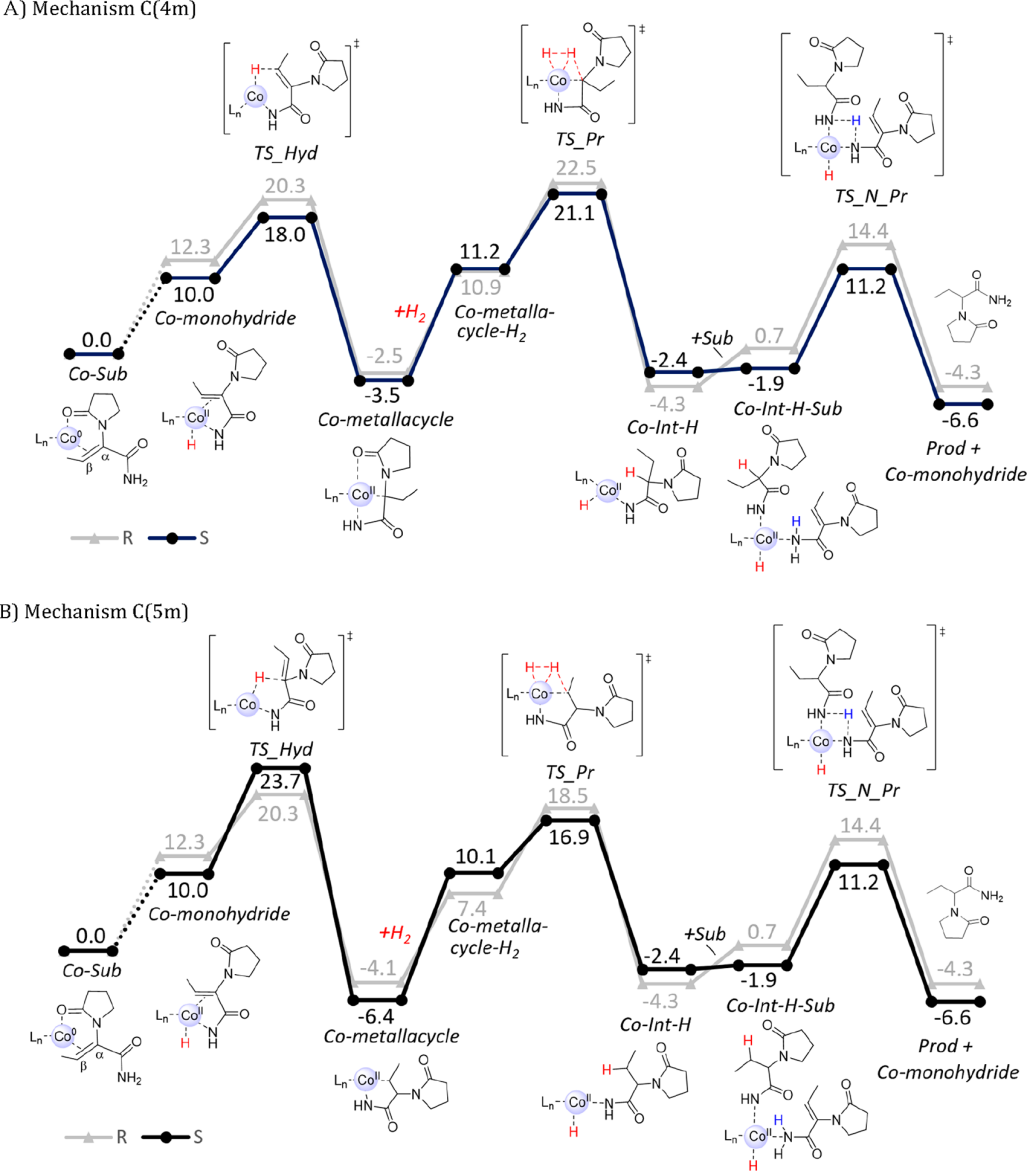
Metallacycle mechanisms (A) **C**(4m) and (B) **C**(5m) for (*R,R*)-(^Ph^BPE)Co-catalyzed hydrogenation of DHL. Free energies are relative to Co(0)-Sub (in kcal/mol, 323 K, B3LYP-D3/BS2[IEFPCM]//B3LYP-D3/BS1[IEFPCM]). Note that the free (*R*) and (*S*) products have identical energies; however, those of the pro-(*R*)- and pro-(*S*)-Co-monohydrides differ, resulting in the shown energy difference of −2.3 kcal/mol.

**Figure 4:**
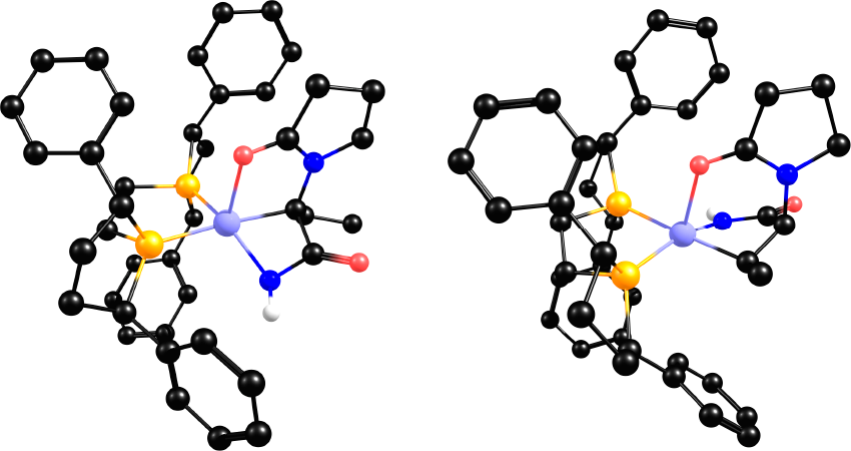
Possible metallacycle intermediates in the (^Ph^BPE)Co-catalyzed hydrogenation of DHL: (left) four-membered aza-metallacycle (initial H^–^ transfer to Cβ, mechanism **C**(4m)); (right) five-membered aza-metallacycle (initial H^–^ transfer to Cα, mechanism **C**(5m)). Hydrogens on carbons are not shown for clarity.

The metallacycle mechanism **C** starts from a Co(II)-monohydride species (Figure 3A), which is 10.0 kcal/mol above the reference structure Co(0)-Sub. Possible pathways for formation of the Co(II)-monohydride are described in Figures S25 and S26 in the Supporting Information and are discussed below. Hydride transfer from the monohydride to the β-atom of DHL has a low barrier and forms an interesting four-membered aza-metallacycle intermediate (mechanism **C**(4m), Figure 4, left). In the next step, H_2_ coordination takes place, followed by proton transfer to the α-atom to form the hydrogenated Co(II)-Int-H intermediate, with a barrier of 24.6 kcal/mol relative to Co(II)-metallacycle. The proton transfer step is rate- and selectivity-determining for mechanism **C**(4m).^66^ In the final step, coordination of another substrate allows for a low-barrier proton transfer to the nitrogen atom of the substrate (TS_N_Pr), resulting in the final product and the regeneration of the Co(II)-monohydride.

Metallacycle mechanism **C** was also tested with an initial hydride transfer from the Co-monohydride to the Cα atom of DHL (mechanism **C**(5m), Figure 3B and Figure S24). The formed intermediate is a five-membered aza-metallacycle species (Figure 4, right). The following steps are the same as for mechanism **C**(4m), with the only difference being that the subsequent proton transfer occurs to the Cβ atom, with an overall rate-limiting barrier of 23.7 kcal/mol for formation of the (*S*)-product.^67^

**Scheme 4:**
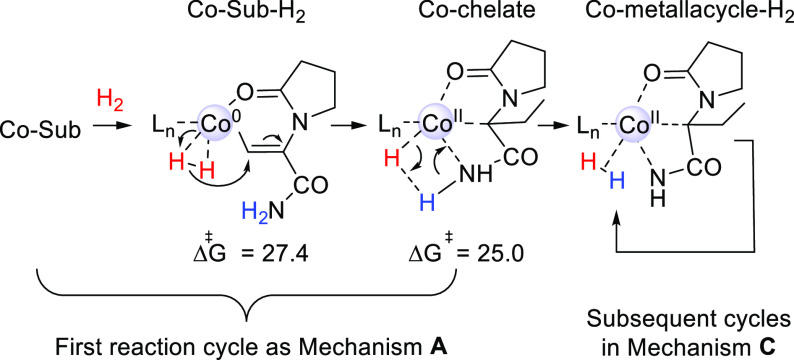
Proposed Route for the Initial Transformation of the Co(0)-Sub Species to an Intermediate in Mechanism **C** Energies (kcal/mol) were obtained with *dehydro*-levetiracetam (DHL).

The computed energies indicate that, for (^Ph^BPE)Co-catalyzed hydrogenation of DHL, both four-membered and five-membered aza-metallacycle mechanisms **C** are energetically feasible at 323 K, with computed barriers of ∼25 kcal/mol. However, a relevant question is how the active monohydride species initially could be formed in mechanism **C**. In the Co-dialkyl-mediated hydrogenation of hydroxylated alkenes, we proposed that a Co(II)-monohydride species can be formed from the Co(II) precatalyst through protonation and loss of the alkyl ligands.^10^ However, for the current system, the starting complex is a Co(0) species with a neutral ligand,^34^ making it less obvious how a Co(II)-monohydride can be formed. A direct oxidative addition of the ionizable group of the substrate to Co(0) is too costly (Figure S25 in the Supporting Information). Instead, we propose that the reaction starts from the Co(0)-enamide species, which binds H_2_ and undergoes a hydride transfer (Scheme 4). The formed hydride may then abstract a proton from the ionizable group of the substrate (−NH_2_ for DHL), resulting in formation of the aza-metallacycle that is part of mechanism **C**. The barrier from Co(0)-Sub to the metallacycle is 27.4 kcal/mol for DHL, making it feasible to occur once at the reaction temperature. After the aza-metallacycle is formed, mechanism **C** can operate in subsequent reaction cycles (overall barrier 24.6 kcal/mol). One can also envision alternative precatalytic pathways, where the solvent MeOH mediates proton transfer from NH_2_ of Co(0)-Sub to either the Cα or Cβ atom of the enamide (Figure S26 in the Supporting Information).

In conclusion, the computations indicate that the metallacycle mechanism **C** is energetically preferred for (^Ph^BPE)Co-catalyzed hydrogenation of DHL (barriers of 23.7–24.6 kcal/mol for the (*S*) pathways, Figure 3); however, it needs to be emphasized that also the classic redox path **A** appears to be within reach (barrier of 27.4 kcal/mol for the (*S*)-path, Scheme 3).

**Figure 5:**
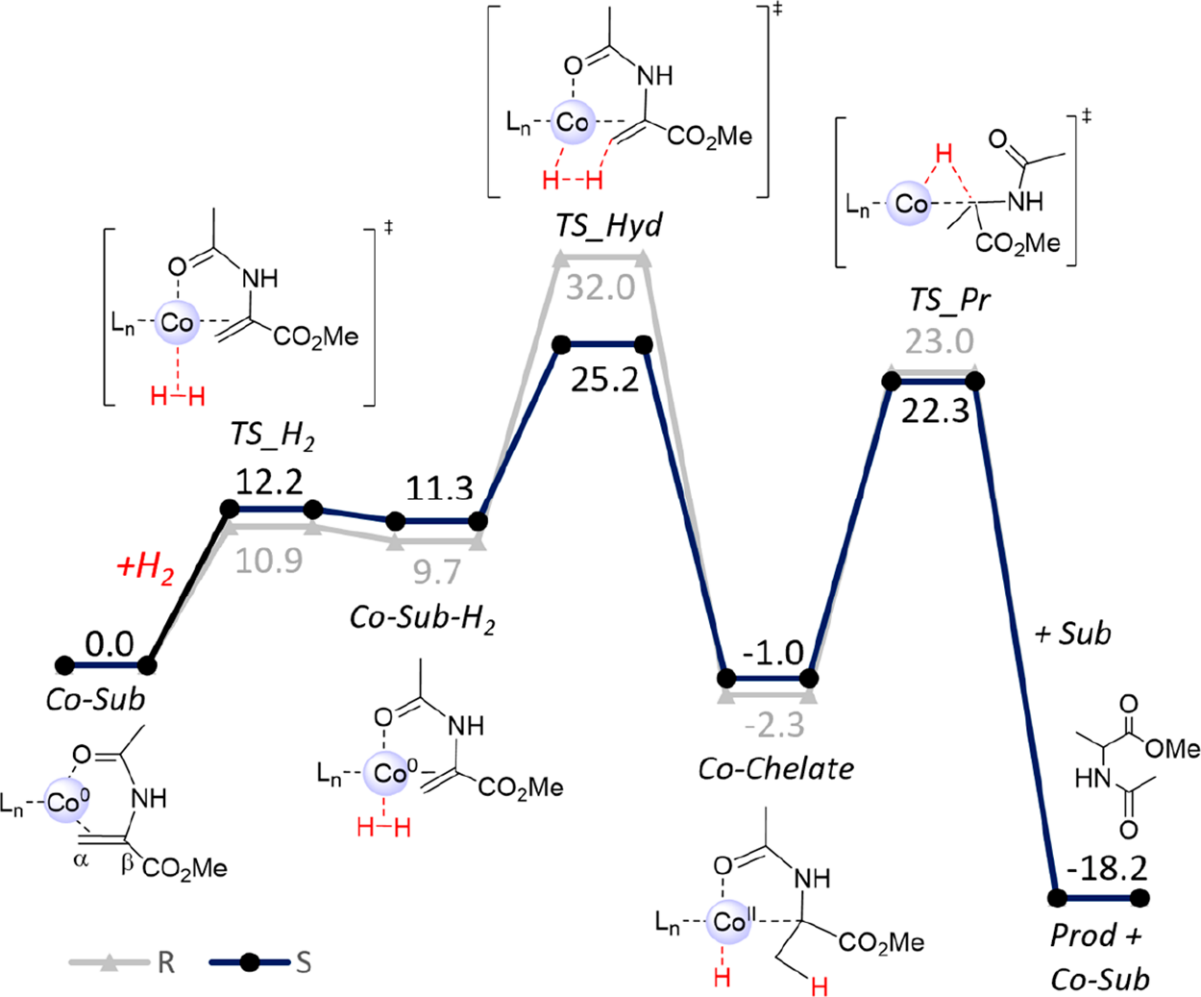
Computed energy profile (in kcal/mol, 323 K, B3LYP-D3/BS2[IEFPCM]//B3LYP-D3//BS1[IEFPCM]) for the (*R,R*)-(^Ph^BPE)Co-catalyzed hydrogenation of MAA via redox mechanism **A** (energies in the absence of explicit MeOH).

For the enamide MAA, comparable calculations were performed on all four mechanistic possibilities **A–D**. The overall barrier for pathway **A** is 25.2 kcal/mol for the formation of the *S* product via initial hydride transfer to the Cβ atom, with the full energy profile being shown in Figure 5. Hydride transfer to Cα is not feasible, and neither is the alternative mechanism **B** (Figures S27 and S28 in the Supporting Information). Mechanism **C** requires initial formation of a Co-monohydride, with the catalytic reaction proceeding through hydride transfer to Cα of MAA and formation of a six-membered metallacycle, with an overall barrier of 24.9 kcal/mol relative to Co(0)-enamide (mechanism **C**(6m), Figure S29 in the Supporting Information). It should be noted that transfer of a hydride to Cβ of MAA via mechanism **C** is not possible; this results instead in a proton transfer and formation of an imine tautomer of MAA (mechanism **C**(imine), Figure S30 in the Supporting Information). This imine can be hydrogenated through the same steps as in mechanism **C**(6m), with a final proton transfer from another substrate to the product and an overall barrier of 25.1 kcal/mol (Figure S30 in the Supporting Information). Hydrogenation of the imine via mechanism **D** as shown in Scheme 2 is not possible, as transfer of a proton from Co-hydride to N is not feasible (Figure S30 in the Supporting Information) and neither is a heterolytic H_2_ cleavage as the final step (Figure S31 in the Supporting Information). In conclusion, for MAA, mechanisms **A** and **C** (both **C**(6m) and **C**(imine)) are energetically accessible, similar to the computational findings for DHL above.

**Figure 6:**
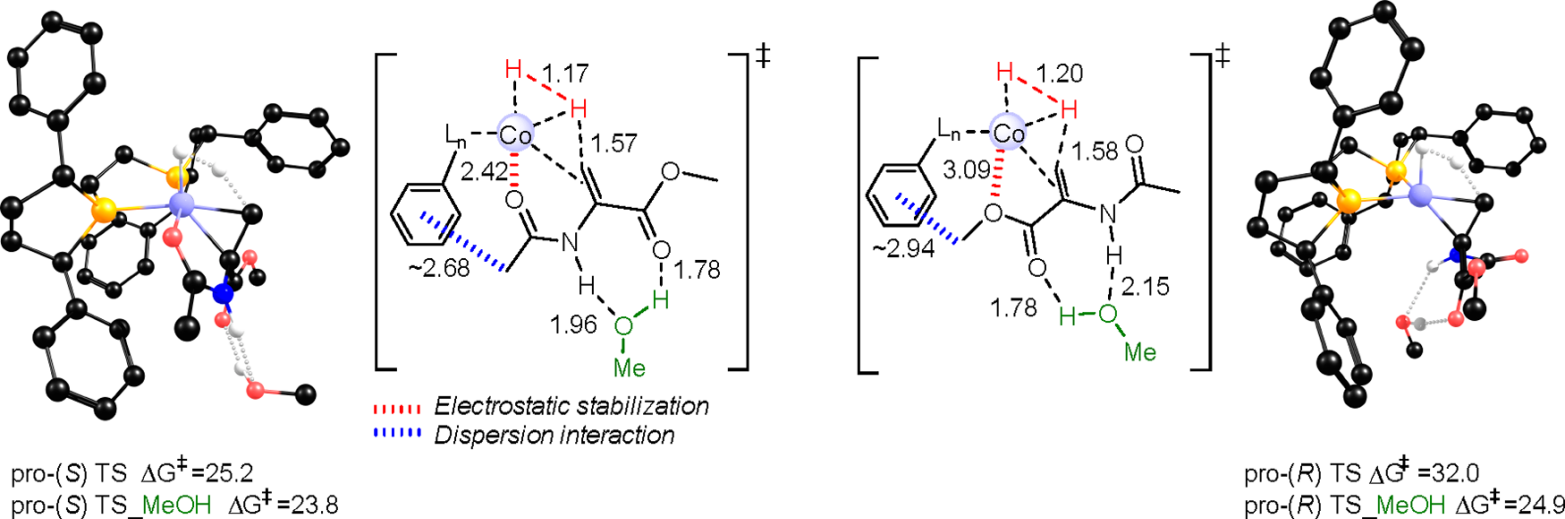
Optimized *pro*-(*S*) (left) and pro-(*R*) (right) hydride transfer TSs for (*R,R*)-(^Ph^BPE)Co-catalyzed hydrogenation of MAA via redox mechanism **A**, with a hydrogen-bonded MeOH molecule (barriers relative to Co(0)-Sub with or without MeOH, respectively, in kcal/mol, 323 K, distances in angstroms). Hydrogens bonded to carbon are omitted for clarity. Electrostatic and dispersion interactions that favor the (*S*)-TS are indicated.

In order to obtain further validation of these mechanistic possibilities, we turned to computing the enantiomeric excesses. This required optimization of all possible (*R*)-pathways for both enamides. Interestingly, during this analysis, the pro-(*R*) and pro-(*S*) transition states showed profound differences. For example, for hydrogenation of MAA via mechanism **A**, the (*S*)-TS shows a different coordination mode of the substrate, where interaction of the amido group with the Co center stabilizes the emerging negative charge on the substrate, whereas at the (*R*)-TS, such a stabilization is not possible (Figure 6). This is reflected in the computed barriers, with the (*R*)-pathway being around 7 kcal/mol higher. On the basis of the experimental results, the (*R*)-product should comprise 4–8% of the product (Table 1),^34^ which appears to be incompatible with the much higher barrier.

This observation led us to explore how explicit solvent, which has the potential to stabilize evolving charges, would affect the computed barriers. To this end, a MeOH molecule was hydrogen-bonded to the NH group of MAA, which was motivated by the X-ray structure of a cationic [(*R,R*)-(^iPr^DuPhos)Co(MAA)][BAr^F^_4_] (BAr^F^_4_ = tetrakis-3,5-bis(trifluoromethyl)phenyl borate) complex, where a solvent molecule (dimethyl ether) is interacting with this NH.^2^ Interestingly, the hydrogen-bonded MeOH decreases the barriers for mechanism **A** (Figure 6 and Figure S36).^2,68^ The decrease is slight for the *S* pathway (1.4 kcal/mol) but significant for the *R* pathway (7.1 kcal/mol, Figure 6), which we ascribe to improved charge stabilization.

It should be emphasized that the inclusion of a solvent molecule brings with it computational complications, because many different conformations are possible, which would require dynamics to evaluate. Thus, the barriers obtained in the presence of MeOH are to be viewed as approximate; however, they indicate that formation of the (*R*)-product via mechanism **A** is feasible under the experimental conditions. Also for mechanisms **C**(6m) and **C**(imine), inclusion of an explicit MeOH molecule hydrogen-bonded to MAA results in a lowering of the barriers by 2–5 kcal/mol (Figures S29 and S30 in the Supporting Information). The obtained results indicate that the solvent may play a vital role in hydrogen-bond stabilization during Co-catalyzed enamide hydrogenation. A similar but smaller barrier reduction in the presence of explicit MeOH is observed for DHL (Figures S24, S37, and S38 in the Supporting Information).

It was also tested if MeOH could open other reaction pathways, for example, coordinate to Co (SI, Figure S40) or donate a proton (SI, Figure S41), but both pathways are excluded on the basis of the computed energies. This is in agreement with earlier deuterium labeling studies that indicate that MeOH remains intact during hydrogenation.^34^

An analysis of the computed enantiomeric excesses with the energetically feasible solvent-assisted pathways is provided in Table 2. We note that in the analysis of e.e. values, we assume Curtin–Hammett conditions, which implies that the e.e. values are only dependent on the barrier heights, not on the relative energies of intermediates.^69,70^ For MAA, mechanisms **A**, **C**(6m) and **C**(imine) all show computed e.e. values in line with the experimental selectivity; thus, the e.e. analysis does not help to discriminate among these mechanisms. For DHL, mechanisms **A** and **C**(5m) show good agreement with the high experimental e.e. of ∼98% (*S*), but mechanism **C**(4m) also provides the correct major isomer of the product (Table 2). It can be noted that both the absolute barriers and the computed e.e. values are somewhat dependent on the DFT functional (Table S3), although the trends are preserved. Our results are in line with work by others, showing that computed e.e. values are sensitive to the DFT functional.^71^ This sensitivity may arise from the fact that the scissile bonds at the TS are described slightly differently by different functionals, leading to small changes in ΔΔ*G*^⧧^ values, which, due to the exponential relationship between the ΔΔ*G*^⧧^ and e.e. values,^72^ can result in significant changes in the e.e. Irrespective of the method applied, the optimized TSs indicate that the main factors leading to the preference for (*S*)-TSs are (i) stabilizing interactions between the carbonyl of the substrate and cobalt and (ii) favorable dispersion interactions between the enamide and the phenyl substituents of the BPE ligand (Figure 6).

**Table 2:** Computed e.e. Values for (*R,R*)-(^Ph^BPE)Co-Catalyzed Hydrogenation of MAA and DHL^a^

substrate	mechanism^b^	e.e._comp_ (%)	e.e._exp_ (%)
MAA	**A**^c^	69.4 [94.6] (*S*)	85–93.0 (*S*)^I^
	**C**(6m)^d^	96.0 [91.5] (*S*)	
	**C**(imine)^e^	91.5 [55.3] (*S*)	
			
DHL	**A**^f^	99.9 [99.7] (*S*)	97–98 (*S*)^I^
	**C**(5m)^g^	86.8 [99.4] *(S*)	
	**C**(4m)^h^	49.7 [60.5] *(S*)	

a B3LYP-D3 values are given without brackets, and PBE-D3BJ values are given in brackets (323 K). For the computed barriers see Table S3 in the Supporting Information.

b With explicit MeOH.

c Figure S36.

d Figure S29.

e Figure S30.

f Figure S37.

g Figure S24.

h Figure S38.

I Table 1.

**Figure 7:**
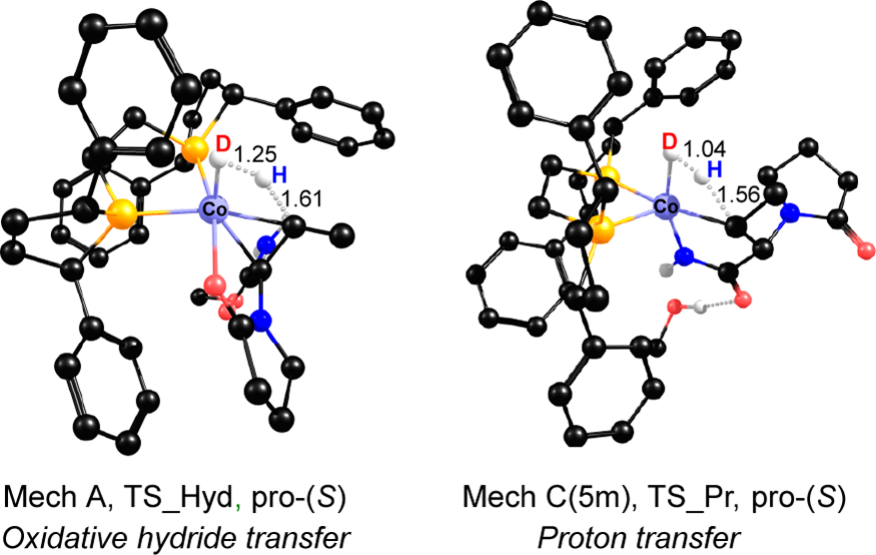
Splitting of HD during hydrogenation of DHL: (left) Mechanism **A**, oxidative hydride transfer (TS_Hyd); (right) mechanism **C**(5m), proton transfer (TS_Pr). Distances are in angstroms.

We have further evaluated what deuterium incorporation the TSs involving HD cleavage would predict for the different mechanisms (Table 3). In this analysis, the computed barrier for initial D transfer from HD to the enamide was compared to the barrier for initial H transfer. In all analyzed cases, initial H transfer is energetically preferred. Thus, in order to match the experimental preference for deuterium in the Cα position (Figure 2), only those mechanisms should be relevant, where the Cα position is hydrogenated second. This includes mechanisms **A** and **C**(6m) for MAA, and **A** and **C**(5m) for DHL. The computed deuterium ratios show that the preference for deuterium in the Cα position appears larger for mechanism **C** than for mechanism **A** (Table 3). This may have to do with the nature of the transition state for HD cleavage, which for mechanism **A** involves an oxidative hydride transfer and for mechanism **C** involves a proton transfer from HD to the enamide substrate (Scheme 2). Thus, the scissile bonds at the critical TSs have different natures and lengths (Figure 7) and are affected differently by replacement of hydrogen with deuterium. Interestingly, the computed deuterium ratios are consistently smaller for DHL than for MAA (Table 3), in agreement with the experimental HD partioning results (Figure 2). This may reflect the different nature of the C–H/D bonds that are formed in these two substrates during hydrogenation.

**Table 3:** Computed Deuterium Ratio (Cα:Cβ) for (^Ph^BPE)Co-Catalyzed Hydrogenation of MAA and DHL with HD^a^

substrate	mechanism^b^	*D* ratio (α:β)_comp_	*D* ratio (α:β)_exp_
MAA	**A**^c^	1.08:1 [1.12:1]^g^	1.64^i^ (1.35^j^):1
	**C**(6m)^d^	1.56:1 [1.55:1]^g^	
			
DHL	**A**^e^	1.02:1 [1.04:1]^h^	1.20^i^:1
	**C**(5m)^f^	1.40:1 [1.41:1]^h^	

a B3LYP-D3 values are given without brackets, and PBE-D3BJ values are given in brackets (298 K).

b With explicit MeOH.

c Figure S36, TS_Hyd.

d Figure S29, TS_Pr.

e Figure S37, TS_Hyd.

f Figure S24, TS_Pr.

g Calculated assuming 85% (*S*) and 15% (*R*) TSs.

h Based only on (*S*)-TSs.

i *In situ* formed (BPE)Co.

j Preformed (BPE)Co.

The overall DFT and experimental results draw a complex mechanistic picture about (^Ph^BPE)Co-catalyzed hydrogenation of enamides. However, by combining the different insights, we can make the following conclusions. For DHL, mechanism **B** (Figure S22 in the Supporting Information) has a barrier that is too high and mechanism **D** is not possible due to the substrate structure. Mechanism **C**(4m) (Figure S38 in the Supporting Information) shows both a computed e.e. that is too low (Table 2) and an initial H transfer from H_2_ to Cα, in disagreement with the HD labeling results (Figure 2). Further, for this substrate, no HH or DD products were formed during the HD labeling, which would rule out mechanism **C**(5m) (Figure S24 in the Supporting Information). This leaves mechanism **A** (Scheme 3 and Figure S37 in the Supporting Information) as the most likely pathway for (^Ph^BPE)Co-catalyzed hydrogenation of DHL. In computations, mechanism **A** provides good agreement with the experimental e.e. and reasonable agreement with HD partitioning results for DHL (Tables 2 and 3).

For MAA, mechanisms **B** and **D** (Figure S28 and S30 in the Supporting Information) have barriers that are too high. Mechanism **C**(imine) (Figure S30 in the Supporting Information) shows initial H transfer from H_2_ to Cα, in disagreement with the HD labeling results. Thus, mechanisms **A** (Figure S36 in the Supporting Information) and **C**(6m) (Figure S29 in the Supporting Information) are the most likely for (^Ph^BPE)Co-catalyzed hydrogenation of MAA. The computed e.e. values and HD partitioning ratios (Tables 2 and 3) indicate a preference for **C**(6m), but a clear distinction between the two pathways is not possible.

The conclusions provide the possibility that both the classical redox mechanism **A** and the metallacycle pathway **C** may be accessible for (^Ph^BPE)Co-mediated enamide hydrogenation. This seems to be in contrast to (^iPr^DuPhos)Co, which only can access the classical redox mechanism **A**.^52^ The results indicate that the nature of the phosphine ligand could influence which hydrogenation pathway is operative. A decisive factor would be if the Co(II)-monohydride species essential for metallacycle mechanism **C** can be formed from the resting state under reaction conditions. Although our computed energies indicate that this may be possible, we do note that, for both MAA and DHL, the (^Ph^BPE)Co-monohydride is ∼10 kcal/mol higher in energy than the (^Ph^BPE)Co(0)-enamide resting state (Figure 3 and Figure S29), indicating that the equilibrium would be toward the latter. In contrast, with hydroxylated alkenes as substrates, the Co(II)-monohydride and the Co(0)-alkene are equienergetic, making a metallacycle mechanism more likely to occur.^10^ Thus, also the type of substrate should heavily influence which of the energetically accessible mechanistic pathways, **A** and **C**, are operative in Co-mediated hydrogenations of unsaturated substrates.

## Conclusions

The intimate details of (^Ph^BPE)Co-catalyzed hydrogenation of enamides have been investigated. Although the computational and experimental results indicate the possible presence of multiple competing mechanisms, clear trends can be identified. Metathesis pathway **B** and imine pathway **D** are excluded for both substrates, while the classical redox mechanism **A** and metallacycle pathway **C** are energetically feasible, as shown in DFT calculations. A significant difference between the two substrates is the type of metallacycle intermediate that they form, with four- and five-membered aza-metallacycles for DHL and a six-membered metallacycle for MAA. HD labeling results indicate that mechanisms **A** and **C**(6m) are both possible for MAA, whereas for DHL formation of only the HD (no HH or DD) product indicates a preference for mechanism **A**.

The original experimental screening of Co-catalyzed enamide hydrogenation displayed a significant effect of the solvent on the observed enantioselectivities, with e.e. values varying from 76 to 94% (*S*) for DHL at RT in different solvents (MeOH, EtOH, iPrOH, TFE).^34^ Our work shows that computational models, which include an explicit MeOH solvent molecule hydrogen-bonded to the enamide, lower critical barriers and provide computed e.e. values in line with the experimental results. Thus, our computations identify a possible role of the protic solvent in Co-catalyzed enamide hydrogenation.^34^

The overall results obtained for bis(phosphine)-Co-catalyzed hydrogenation of enamides highlight the fact that nonprecious metals may show highly complex mechanistic scenarios with competing redox and nonredox reaction pathways. Which mechanism in the end will be operative may be affected by the nature of the bis(phosphine) ligand, the substrate, and the solvent.

## Supporting Information Available

Additional computational results and experimental details as described in the main text (PDF)
Optimized coordinates, which can be conveniently visualized with the Mercury program from the Cambridge Crystallographic Data Centre (XYZ)

## Supplementary Material

om2c00180_si_001.pdf

om2c00180_si_002.xyz
