# Supplementary material for: Cobalt-Catalyzed Asymmetric Hydrogenation of Enamides: Insights into Mechanisms and Solvent Effects
Source: Organometallics. 2022 Jul 25;41(14):1872–82. doi: 10.1021/acs.organomet.2c00180 (PMC9335863; doi:10.1021/acs.organomet.2c00180)
Supplement: Supplementary file 1 — om2c00180_si_001.pdf [file om2c00180_si_001.pdf]

Supporting information

Cobalt-Catalyzed Asymmetric Hydrogenation of Enamides: Insights into Mechanisms and Solvent Effects

Ljiljana Pavlovic,[a] Lauren N. Mendelsohn,[b] Hongyu Zhong,[b] Paul J. Chirik,*[b] and Kathrin H. Hopmann*[a]

[a]Department of Chemistry, UiT - The Arctic University of Norway, N-9037 Tromsø, Norway
[b]Department of Chemistry, Princeton University, New Jersey 08544, United States.

Corresponding Authors: kathrin.hopmann@uit.no and pchirik@princeton.edu

CONTENTS

1. Experimental results 3

2. General Considerations 3

3. Hydrogenation of MAA 4

4. HD experiments 6

5. H_2/D_2 Scrambling 17

6. Computational details 19

7. Evaluation of the Co-Substrate interaction strength 20

8. Alternative mechanisms for the hydrogenation of *dehydro*-levetiracetam (DHL) 21

9. Redox Co(0)-Co(II) mechanism A for the hydrogenation of DHL 21

10. σ-bond metathesis mechanism B for the hydrogenation of DHL 22

11. Computed mechanism for the hydrogenation of DHL via mechanism C with a 4-membered metallacycle (mechanism C(m4)) 23

12. Mechanism C for the hydrogenation of DHL with hydride transfer to Cα and formation of a 5-membered metallacycle (mechanism C(m5)) 24

13. Precatalytic pathways for *dehydro*-levetiracetam (DHL) 25

14. A direct oxidative addition of the ionizable group of the substrate to Co(0) giving Co(II)-monohydride 25

15. MeOH-mediated proton transfer from NH_2 of the Co(0)-DHL to give a metallacycle intermediate 26

16. Alternative mechanisms for the hydrogenation of methyl 2-acetamidoacrylate (MAA) 27

17. Mechanism A for MAA with hydride transfer to the Cα atom 27

18. σ-bond metathesis mechanism B for MAA; hydride transfer to Cβ 28

19. 6-membered metallacycle mechanism C(6m) for MAA, hydride transfer to the Cα 29

20. C(imine) and D mechanisms for MAA 30

21. Alternative C(imine, heterolytic) mechanism for MAA with heterolytic H_2 cleavage 31

22. Precatalytic pathways for methyl 2-acetamidoacrylate (MAA) 32

23. A direct oxidative addition of the ionizable group of MAA to Co(0), giving Co(II)-monohydride in the presence of explicit solvent 32

24. Proposed mechanism for the formation of the active Co(II)-metallacycle_H_2 species via an imine intermediate 33

25. MeOH mediates proton transfer from NH_2 of the Co(0)-enamide giving either 6-mem. metallacycle or Imine intermediates 34

27. Mechanisms for methyl 2-acetamidoacrylate (MAA) and *dehydro*-levetiracetam (DHL) with MeOH 36

28. Mechanism A for MAA with explicit MeOH 36

29. Rate-limiting TSs (Hyd transfer) via mechanism A for DHL with explicit MeOH 37

30. Metallacycle mechanism C (via a 4-membered metallacycle intermediate) for DHL with explicit MeOH 38

31. An alternative metallacycle mechanism C (4m) for DHL where MeOH delivers a proton to the nitrogen of the product 39

32. Alternative mechanism A for MAA with one MeOH molecule coordinated to Co 40

33. Alternative mechanism for MAA with MeOH as proton donor 41

34. The computed barriers and enantiomeric excesses for DHL and MAA 42

35. Additional computational methods (PBE0-D3BJ and ωB97XD) with ^{Ph}BPE ligand for DHL and MAA 43

36. Evaluation of quartet spin states 44

37. REFERENCES 45

1. Experimental results

2. General Considerations

All air- and moisture-sensitive manipulations were carried out using vacuum line, Schlenk and cannula techniques or in an MBraun inert atmosphere (nitrogen) dry box unless otherwise noted. All glassware was stored in a pre-heated oven prior to use. The solvents used for air- and moisture-sensitive manipulations were dried and deoxygenated using literature procedures.[1] ^{1}H NMR spectra were recorded on an I400 Varian Inova spectrometer operating at 400 MHz. ^{13}C{^{1}H} NMR were recorded on a Bruker A500 spectrometer operating at 126 MHz. ^{31}P{^{1}H} NMR were recorded on an I400 Varian Inova spectrometer operating at 162 MHz. All ^{1}H chemical shifts are reported in ppm relative to $SiMe_4$ using the ^{1}H ($CDCl_3$: 7.26 ppm) chemical shifts of the solvent as a standard. Gas chromatography for the alkane products was performed on a Shimadzu GC-2010 gas chromatograph. GC analyses were performed using a Restek 15 m x 0.25 mm RTX-5 5% diphenyl/95% dimethyl polysiloxane column with a film thickness of 0.25 μm. *Dehydro*-levetiracetam was purchased from Sundia Meditech (Shanghai, China) and used as is. Methyl-2-acetamidoacrylate was purchased from Sigma Aldrich and purified by Et_2O filtration through silica. Both chemicals were dried on a high vacuum line prior to use.

3. Hydrogenation of MAA

In a nitrogen-filled glovebox, a thick-walled glass vessel was charged with MAA (0.014 g, 0.10 mmol), (*S*,*S*)-(^{Ph}BPE)$CoCl_2$ (0.002 g, 0.003 mmol, 3 mol%), Zn (0.007 g, 0.10 mmol, 100 mol%), MeOH (1.5 mL), and a stir bar. The vessel was sealed and removed from the glovebox. On a high-vacuum line, the solution was frozen and the head-space removed under vacuum. The vessel was back-filled with 4 atm of H_2. The solution was sealed, thawed, and stirred at 50 °C in an oil bath for 18 hours. Following this time, the reaction was air-quenched and the solvent evaporated. The crude mixture was taken up in $CDCl_3$ and filtered through an alumina plug. The resulting sample was analyzed by ^1H NMR and chiral GC.

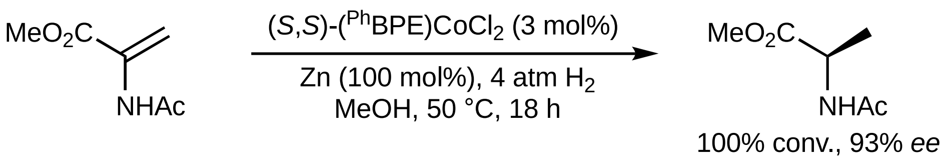

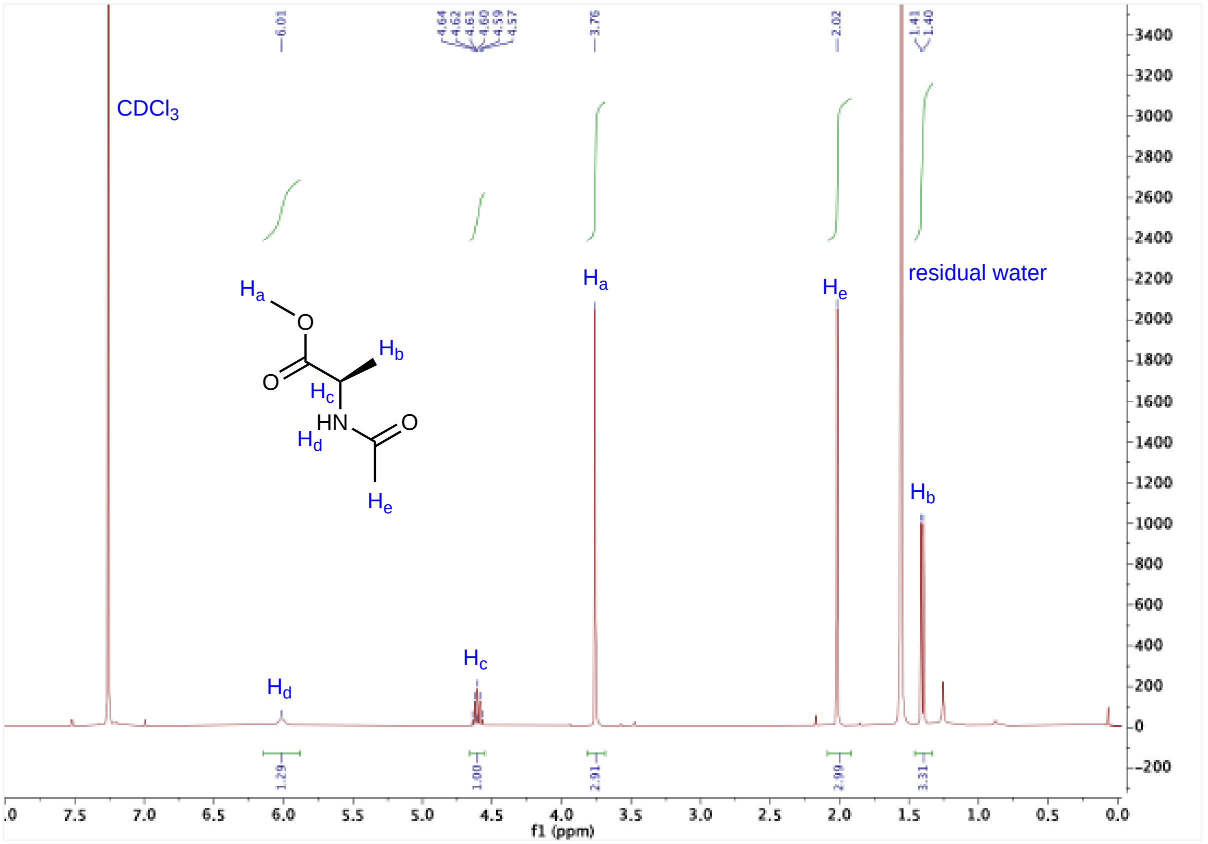

Figure S1. *Top*: Conditions for the hydrogenation of MAA by ^{Ph}BPECoCl$_2$ with Zn reduction, *Bottom*: ^1H NMR spectrum of the resulting solution from hydrogenation of MAA with ^{Ph}BPECoCl$_2$ with Zn reduction.

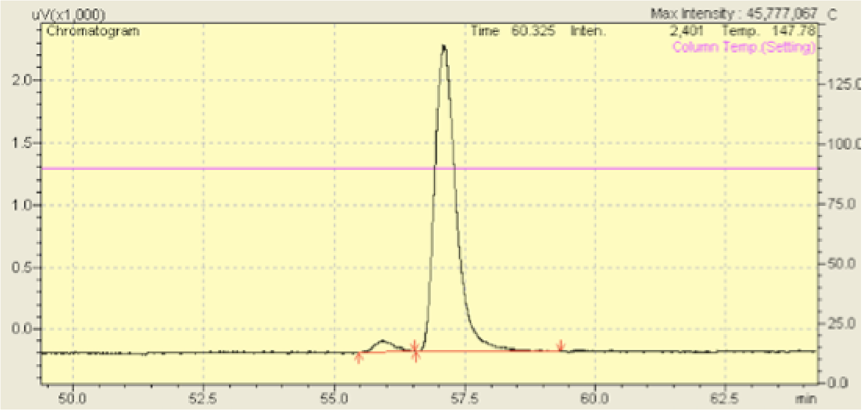

Peak#	Ret.Time	Area
1	55.905	2474.3
2	57.080	70599.9

Figure S2. Chiral GC data for the hydrogenation of MAA by ^{Ph}BPECoCl$_2$ with Zn reduction.

4. HD experiments

MAA

In a nitrogen filled glovebox, a 4 mL vial was charged with a MeOH solution (total volume for each trial was equal to 2 mL) with enamide (0.20 mmol), (*R*,*R*)-(^{Ph}BPE)-Co-(COD) or (*R*,*R*)-(^{Ph}BPE)-$CoCl_2$ (0.04 mmol, 2 mol%; and Zn (20 mol%) when the dihalide was used), and a stir bar. The vial was then placed into a high-pressure reactor, sealed, and removed from the glovebox. The reactor was backfilled with 60 psi of HD and allowed to react for 5 days. At this point the reaction was air-quenched and the volatiles were evaporated under air. The residue was then taken up with EtOAc and filtered through an alumina plug. The solvent was removed, and the residue was taken up in $CHCl_3$ or $CDCl_3$. Deuterium incorporations were determined using ^{1}H, ^{2}H, and quantitative ^{13}C NMR spectroscopy.

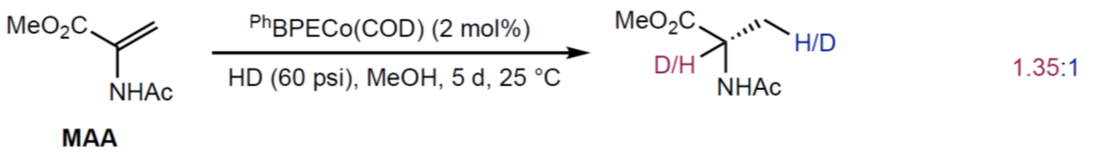

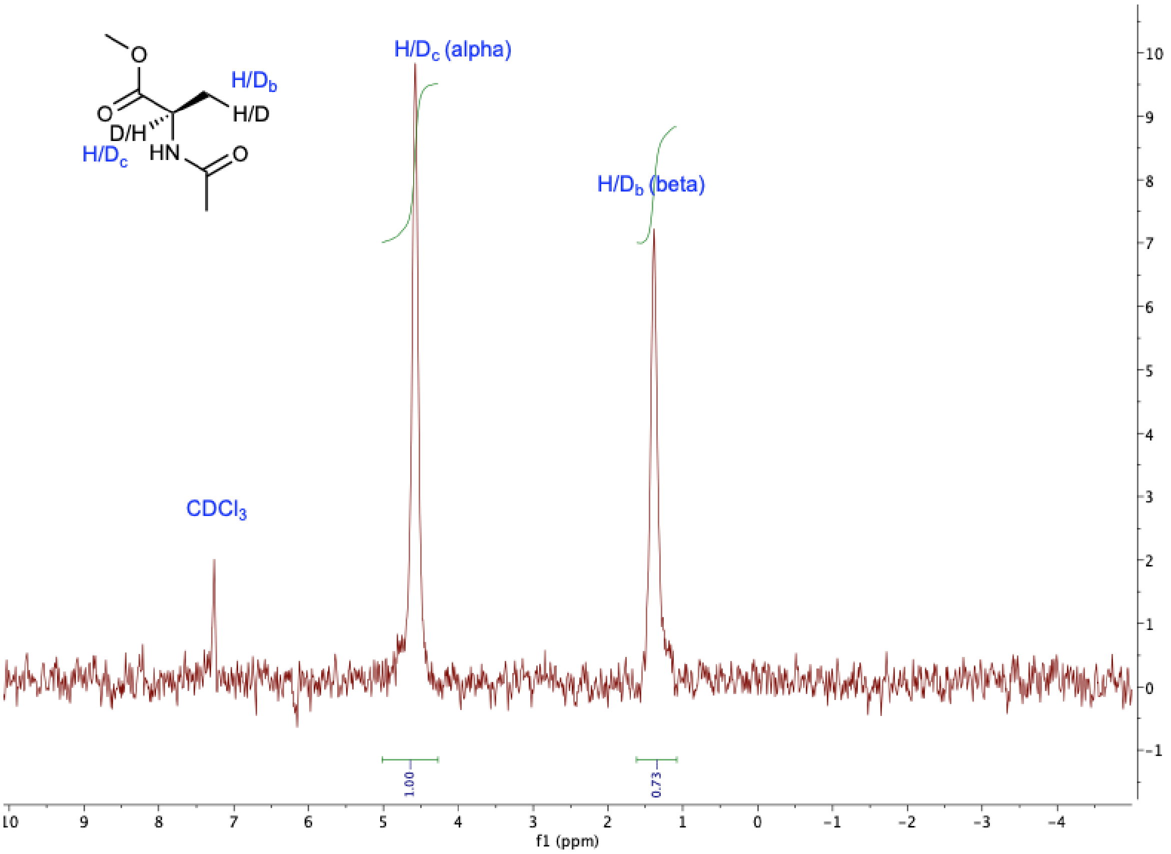

Figure S3. *Top*: Conditions for the ^{Ph}BPE-Co-(COD) catalyzed reaction of MAA with hydrogen deuteride, *Bottom*: ^{2}H NMR of the product of reaction of MAA with HD catalyzed by ^{Ph}BPE-Co-(COD) (rt, $CDCl_3$).

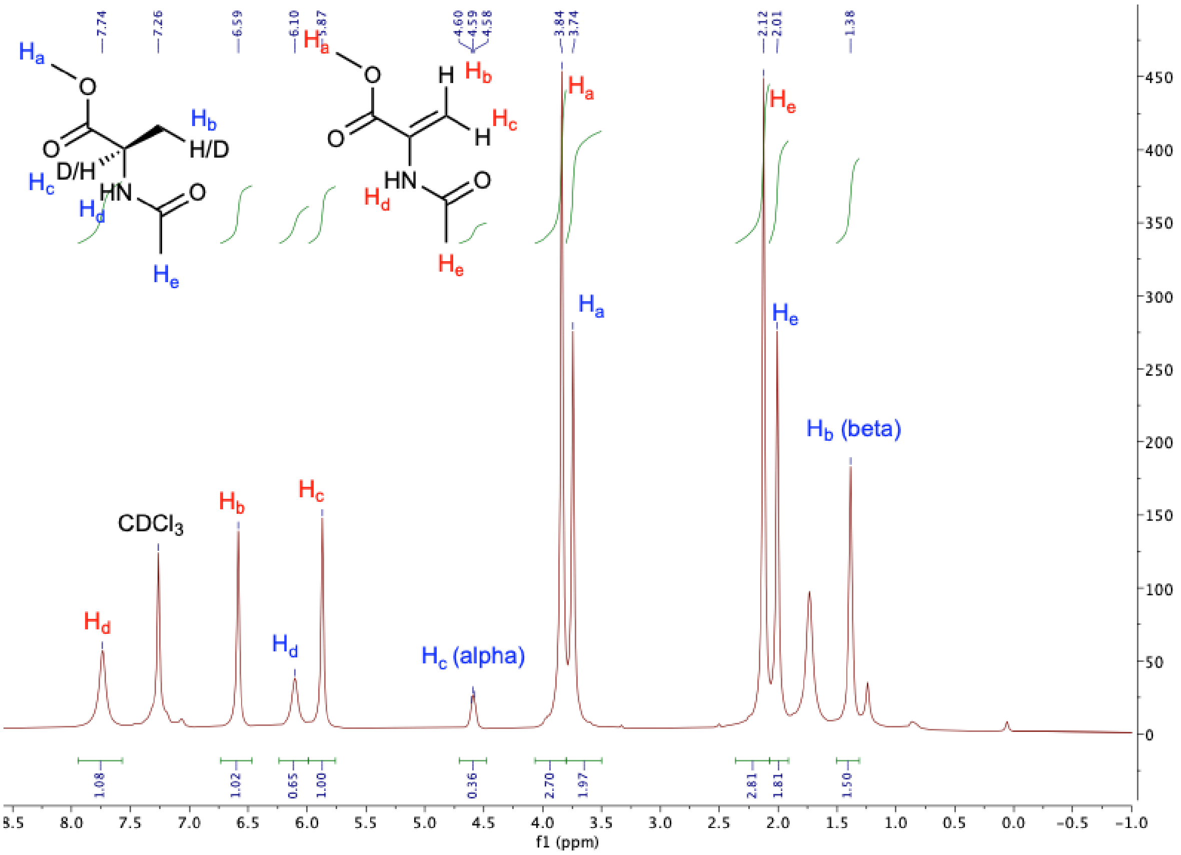

Figure S4. ^{1}H NMR of the product of reaction of MAA with HD catalyzed by [Ph]BPE-Co-(COD) (rt, $CDCl_3$).

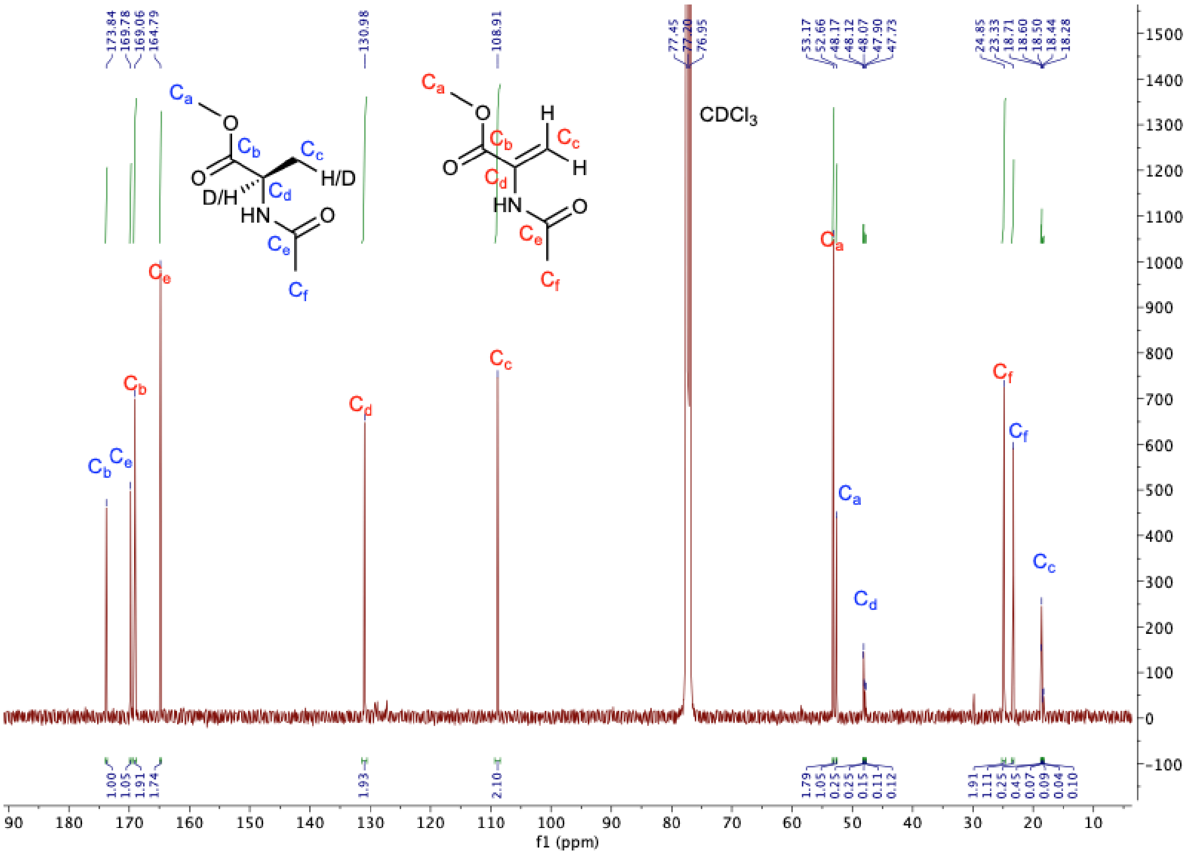

Figure S5. Full quantitative ^{13}C NMR of the product of reaction of MAA with HD catalyzed by ^{Ph}BPE-Co-(COD) (rt, $CDCl_3$).

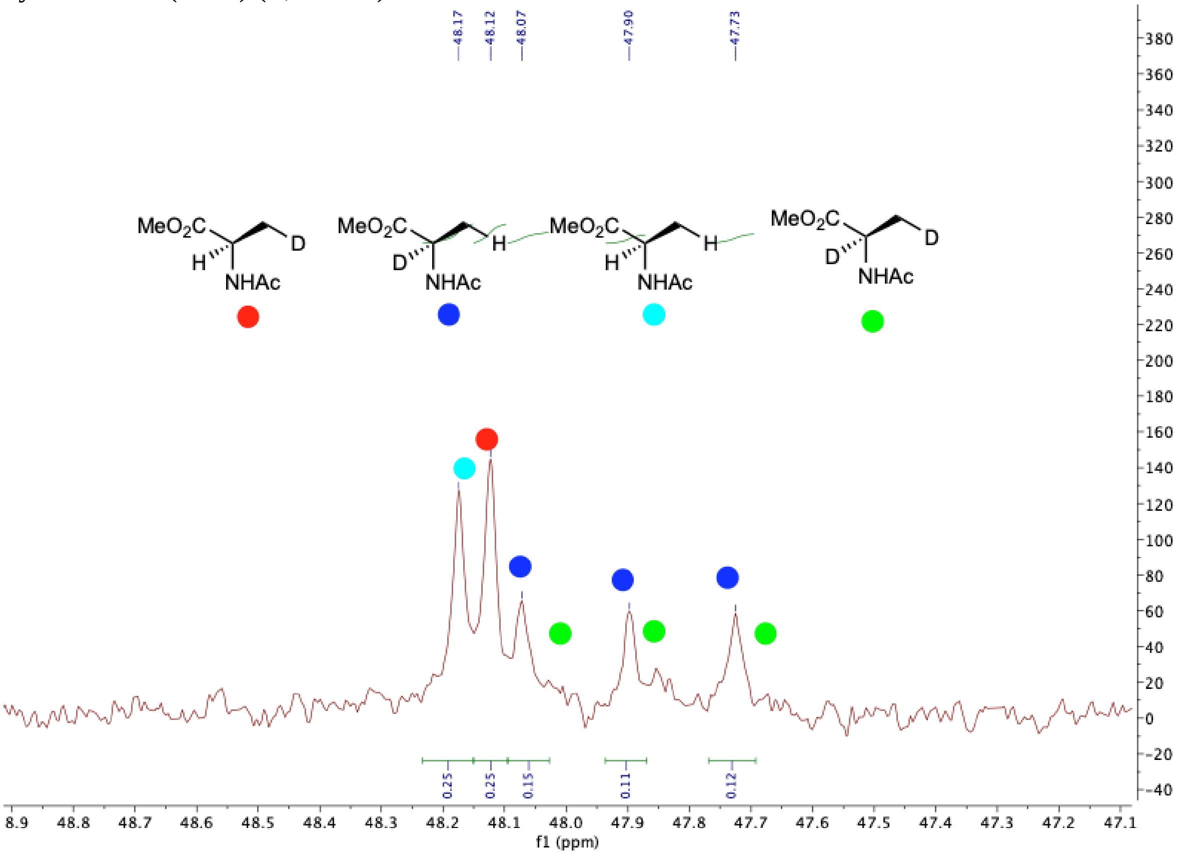

Figure S6. Section of the quantitative ^{13}C NMR of the product of reaction of MAA with HD catalyzed by ^{Ph}BPE-Co-(COD) (rt, $CDCl_3$).

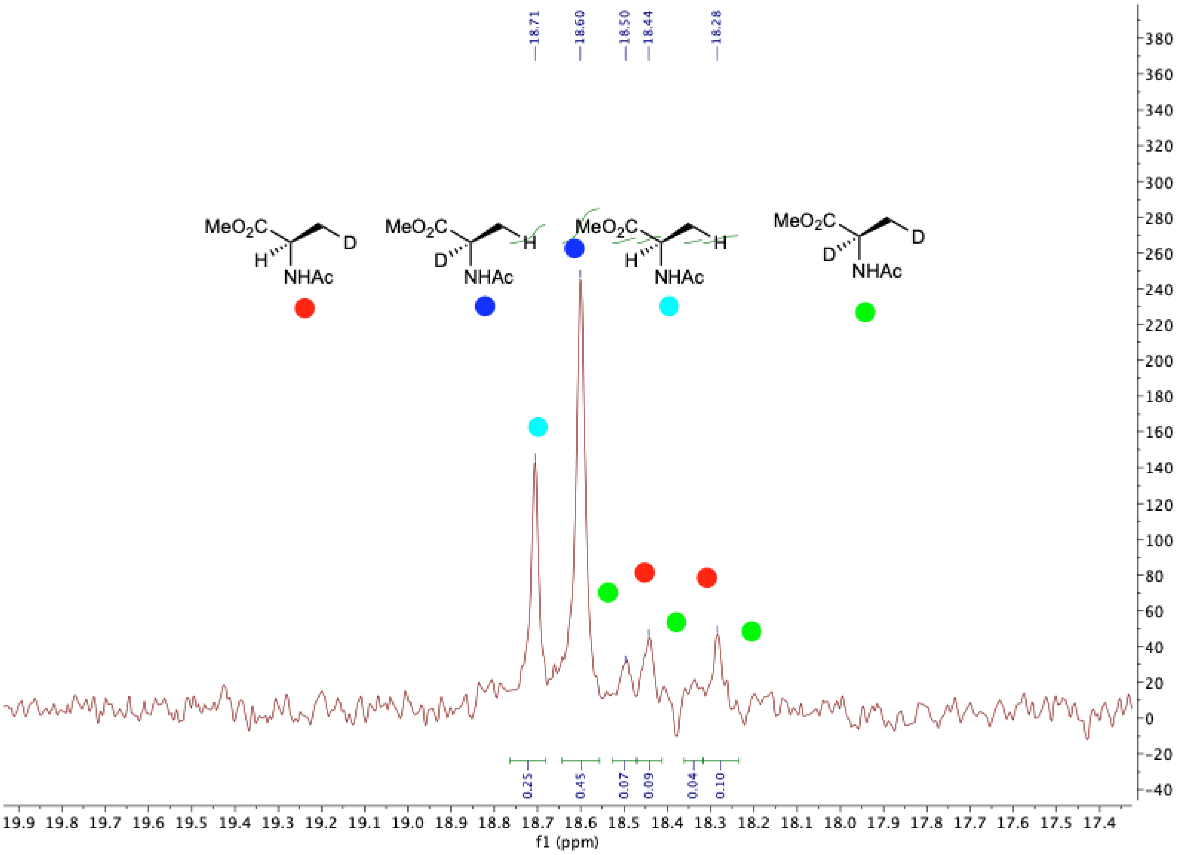

Figure S7. Section of the quantitative ^{13}C NMR of the product of reaction of MAA with HD catalyzed by ^{Ph}BPE-Co-(COD) (rt, $CDCl_3$).

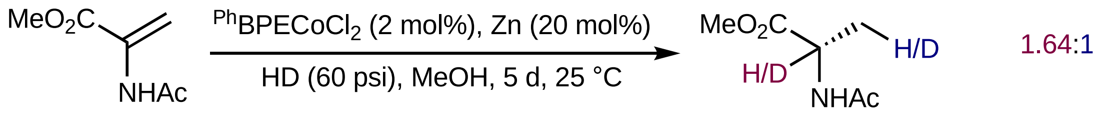

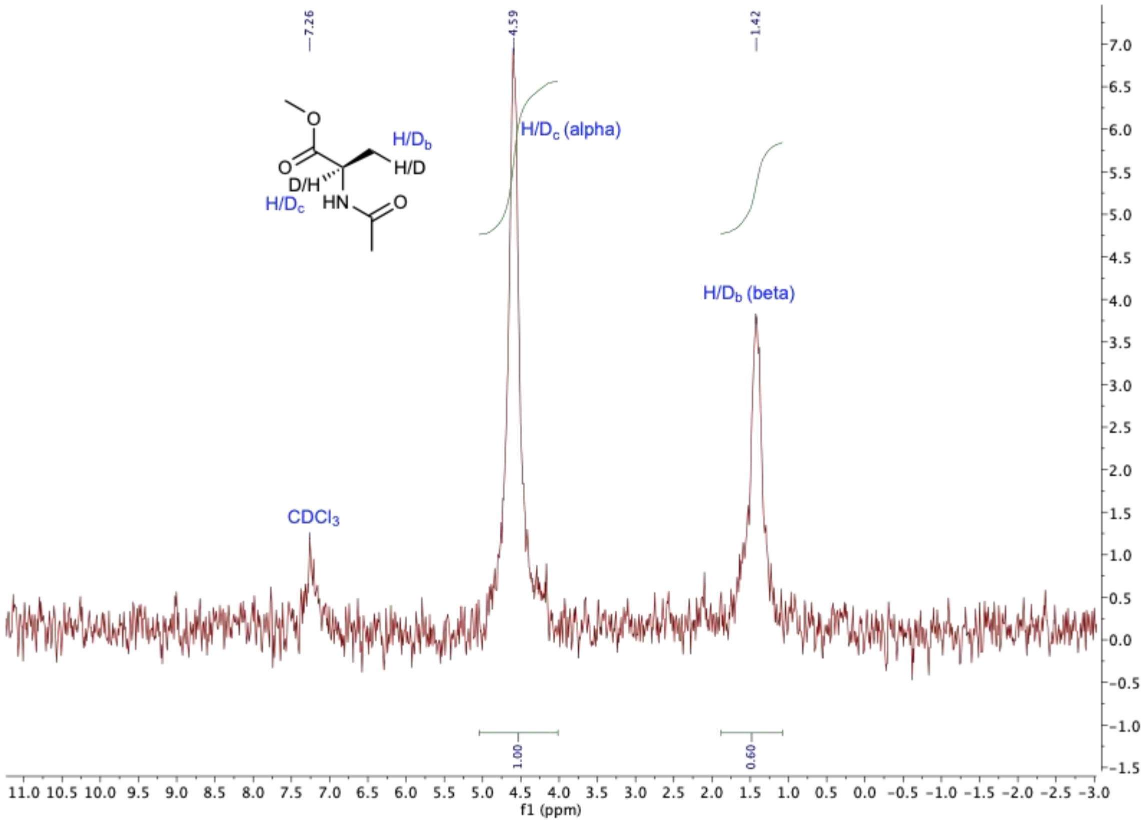

Figure S8. ***Top*:** Conditions for the PhBPE-$CoCl_2$ (with in-situ Zn reduction) catalyzed reaction of MAA with hydrogen deuteride, *Bottom*: ^{2}H NMR of the product of reaction of MAA with HD catalyzed by PhBPE$CoCl_2$ (with in-situ Zn reduction) (rt, $CDCl_3$).

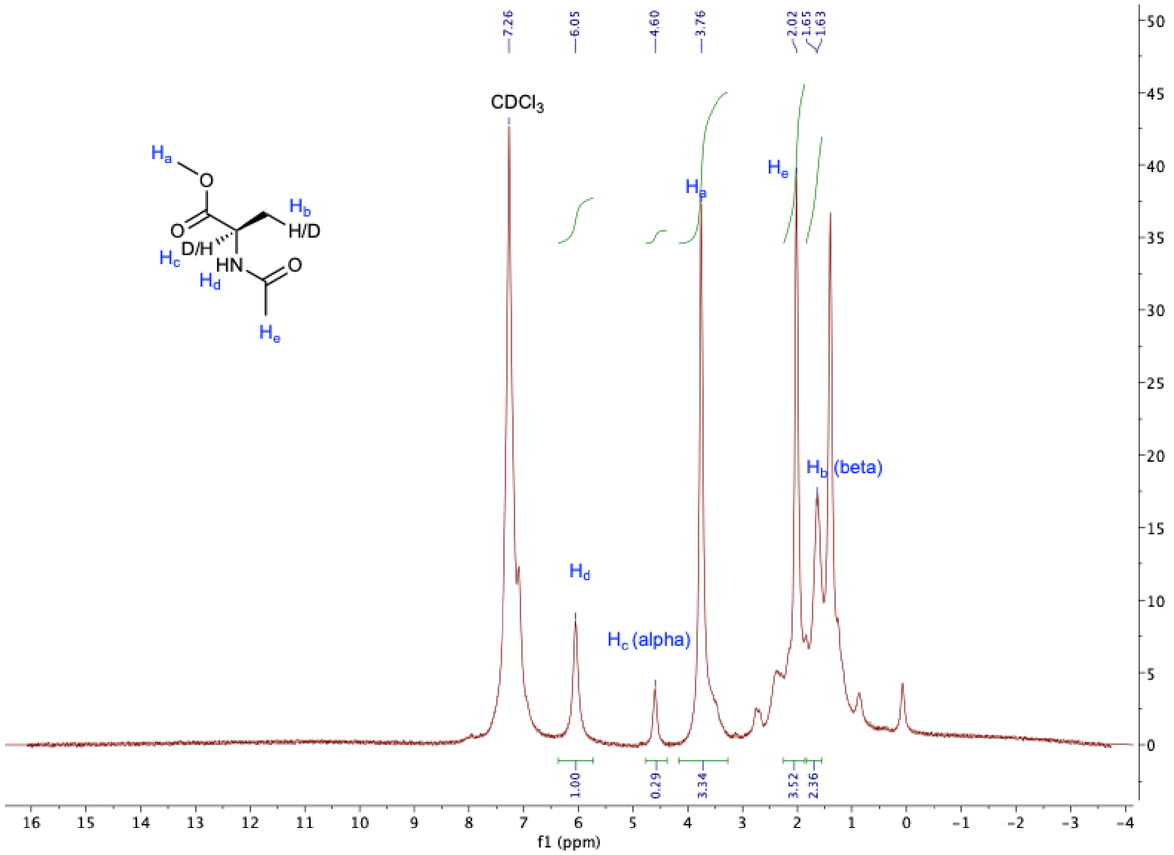

Figure S9. ^{1}H NMR of the product of reaction of MAA with HD catalyzed by ^{Ph}BPE-CoCl$_2$ (with in-situ Zn reduction) (rt, CDCl$_3$).

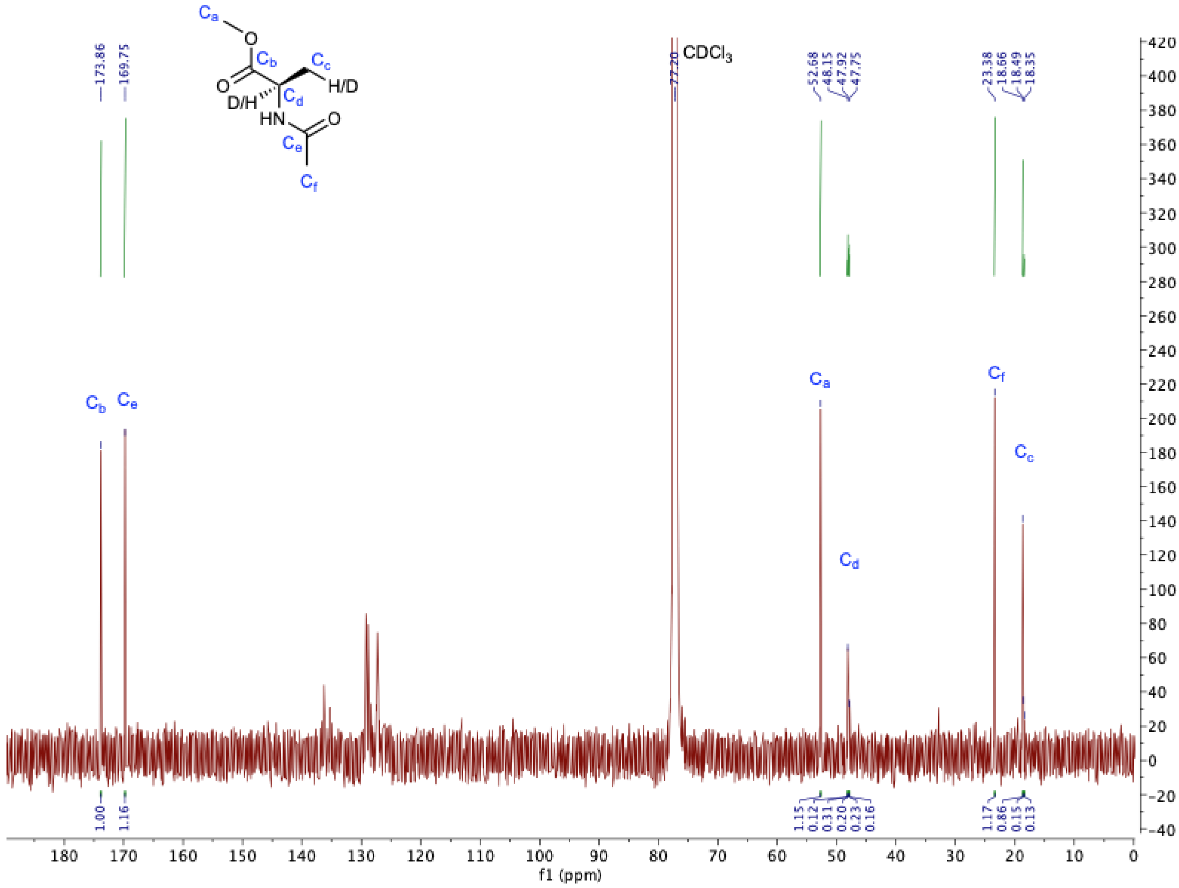

Figure S10. Full quantitative ^{13}C NMR of the product of reaction of MAA with HD catalyzed by ^{Ph}BPE-$CoCl_2$ (with in-situ Zn reduction) (rt, $CDCl_3$).

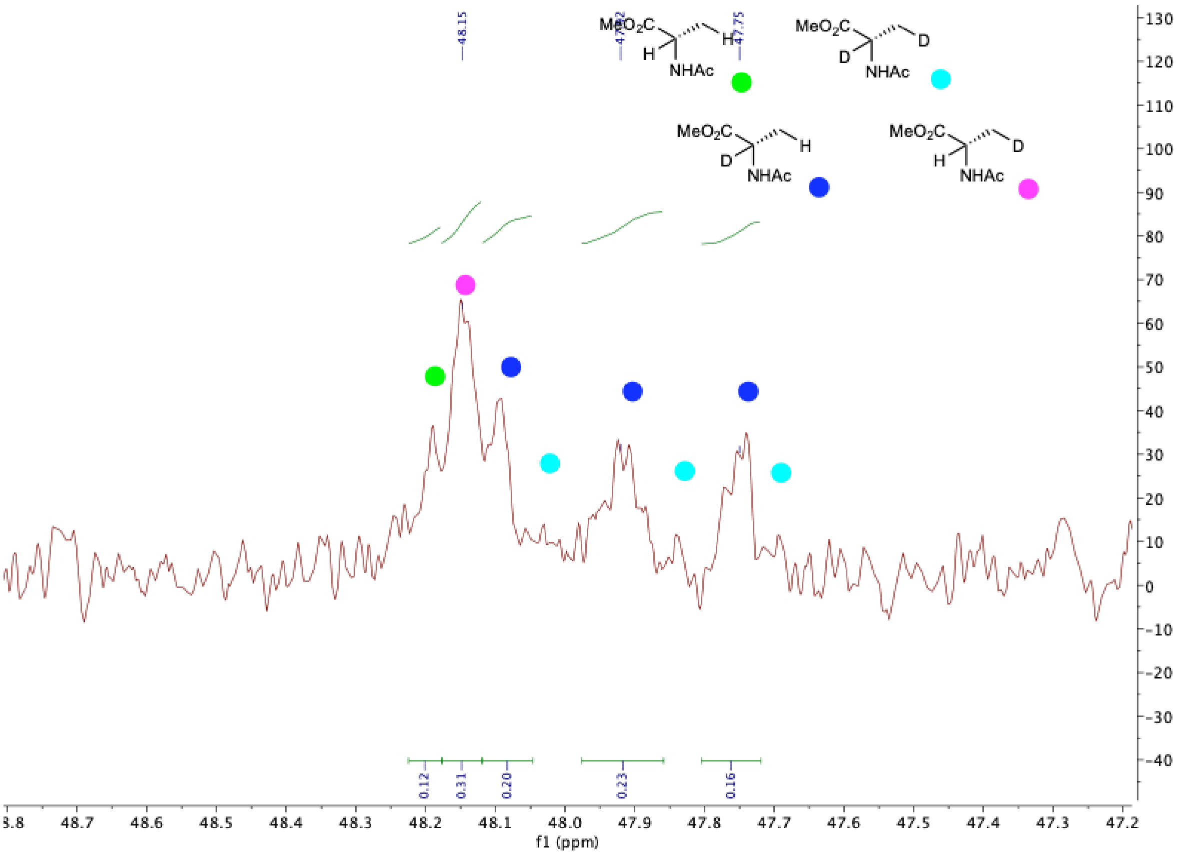

Figure S11. Section of the quantitative ^{13}C NMR of the product of reaction of MAA with HD catalyzed by ^{Ph}BPE-$CoCl_2$ (with in-situ Zn reduction) (rt, $CDCl_3$).

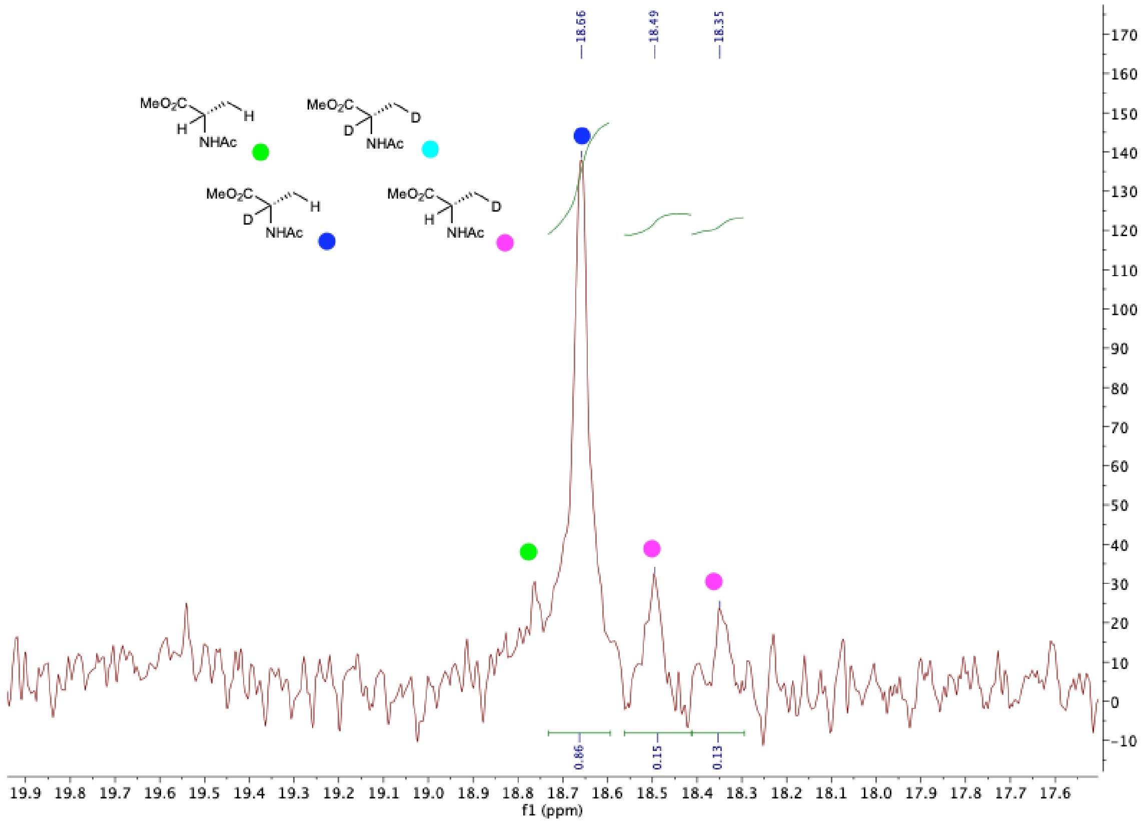

Figure S12. Section of the quantitative ^{13}C NMR of the product of reaction of MAA with HD catalyzed by ^{Ph}BPE-$CoCl_2$ (with in-situ Zn reduction) (rt, $CDCl_3$).

DHL

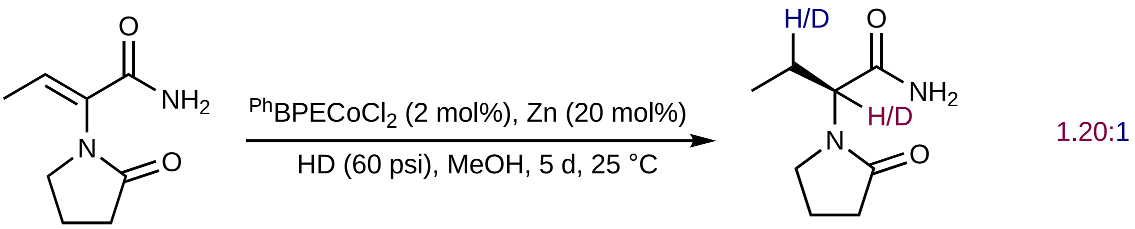

Figure S13. Conditions for the ^{Ph}BPE-$CoCl_2$ (in-situ Zn reduction) catalyzed reaction of DHL with hydrogen deuteride.

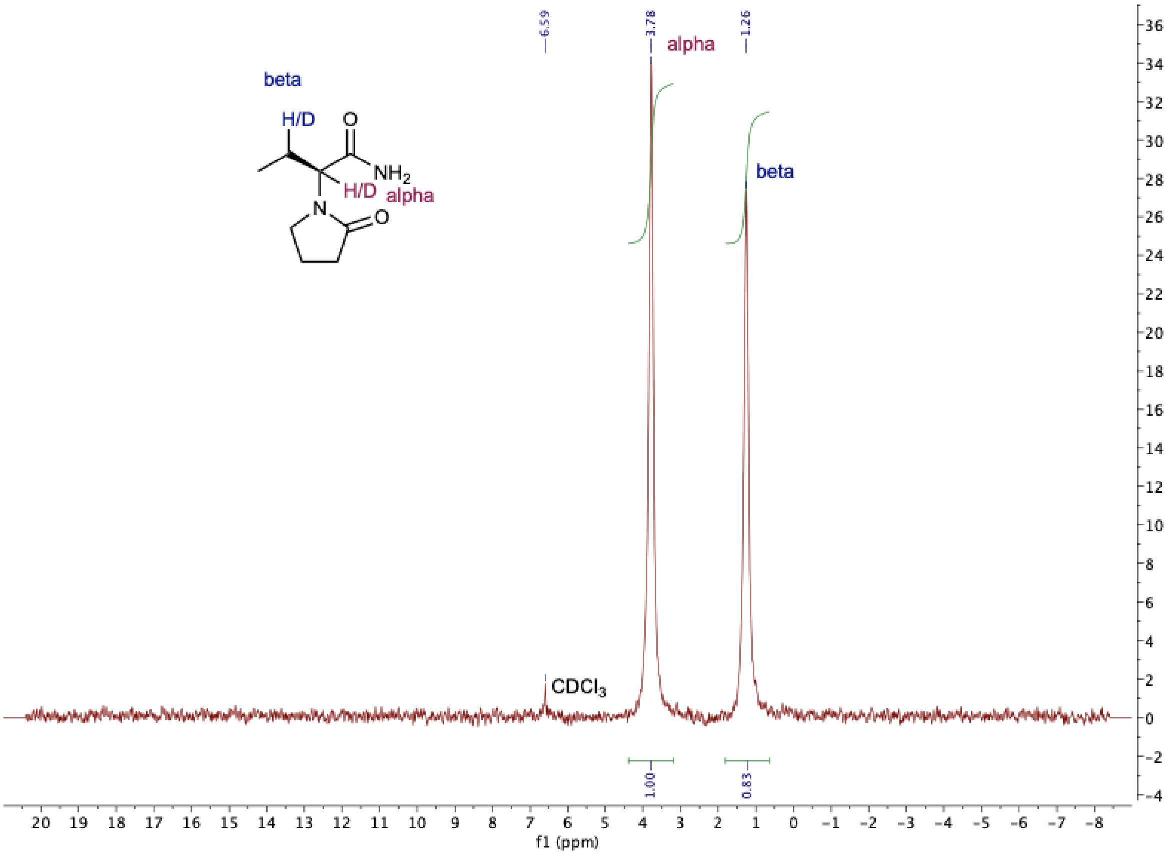

Figure S14. ^{2}H NMR of the product of reaction of DHL with HD catalyzed by ^{Ph}BPE-$CoCl_2$ (with in-situ Zn reduction) (rt, $CDCl_3$).

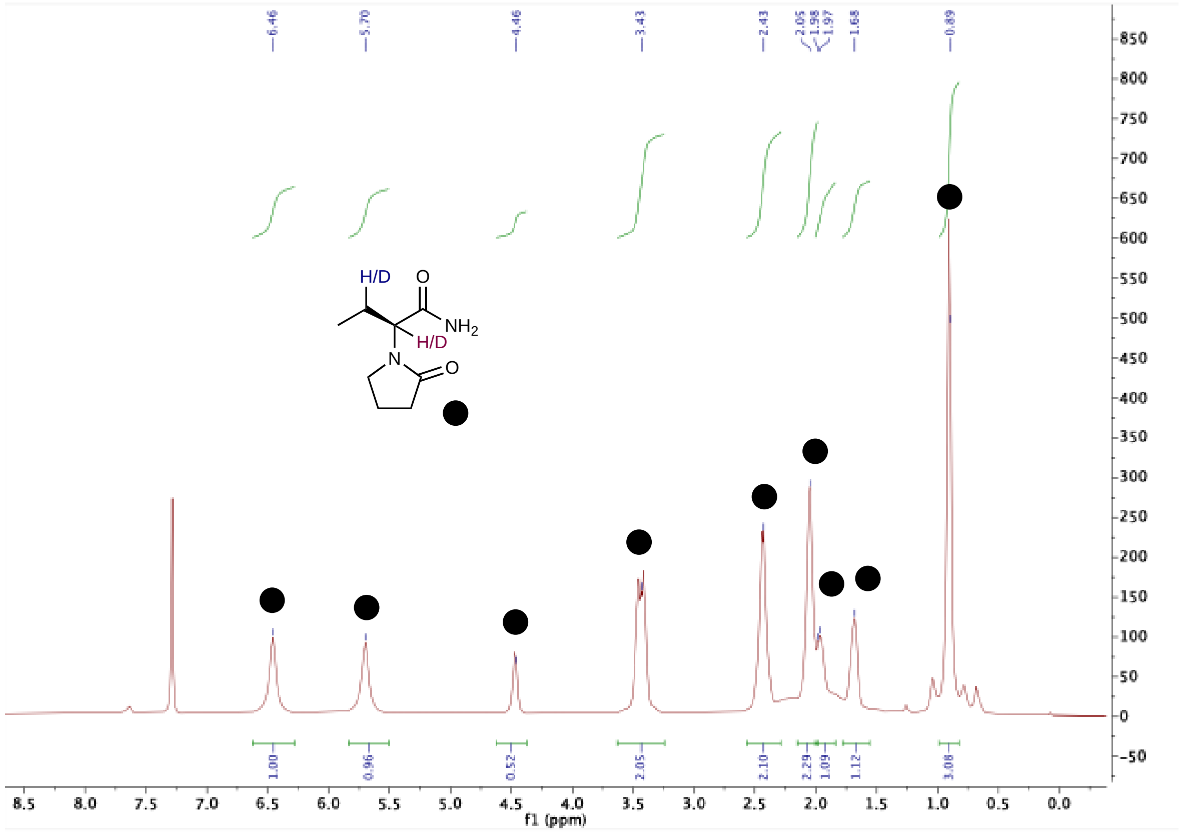

Figure S15. ^{1}H NMR of the product of reaction of DHL with HD catalyzed by ^{Ph}BPE-$CoCl_2$ (with in-situ Zn reduction) (rt, $CDCl_3$).

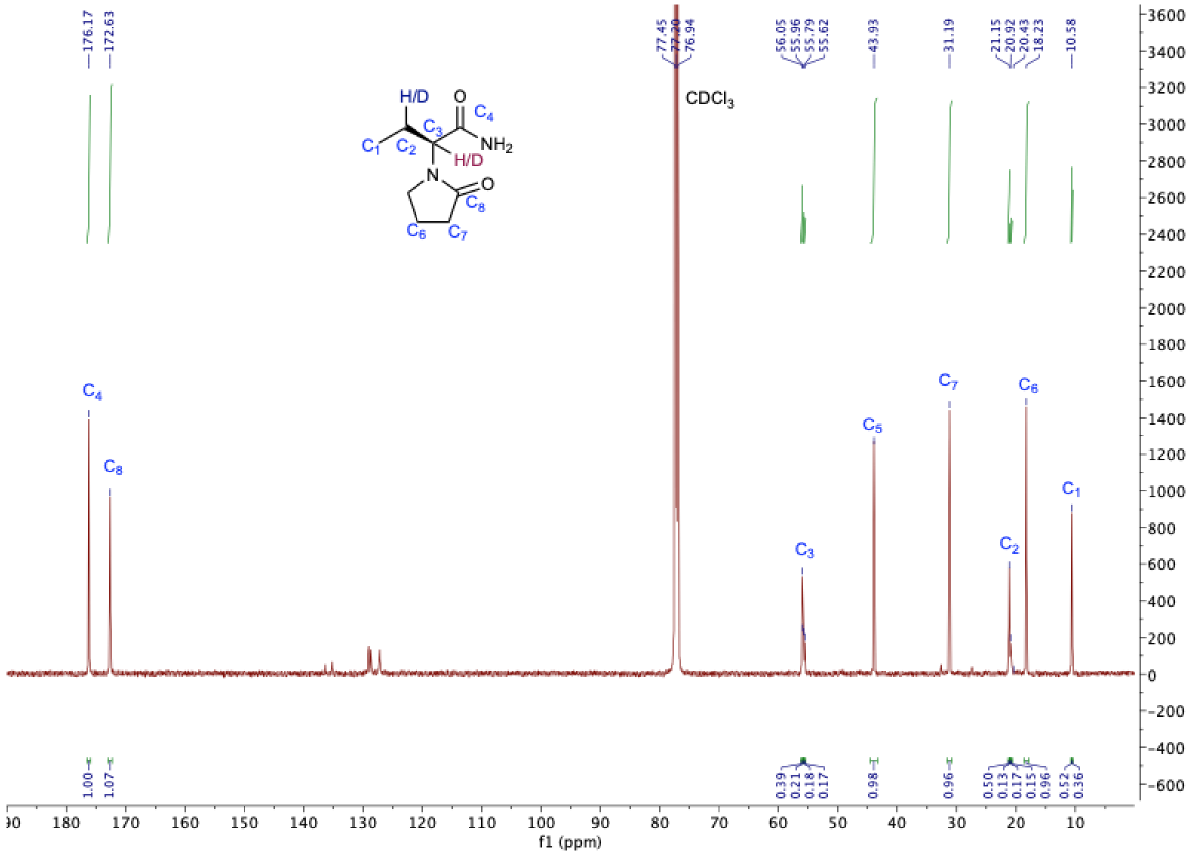

Figure S16. Full quantitative ^{13}C NMR of the product of reaction of DHL with HD catalyzed by $^{Ph}BPE\text{-}CoCl_2$ (with in-situ Zn reduction) (rt, $CDCl_3$).

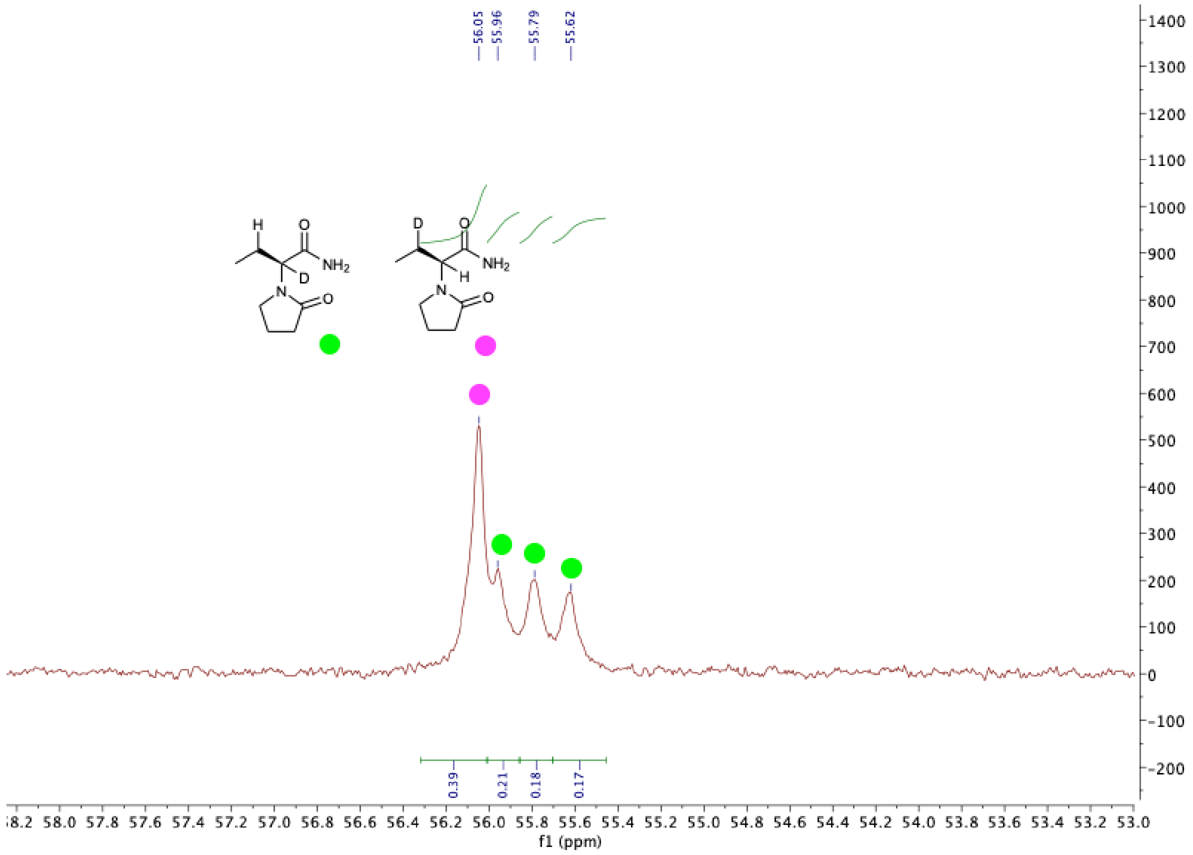

Figure S17. Section of the quantitative ^{13}C NMR of the product of reaction of DHL with HD catalyzed by ^{Ph}BPE-$CoCl_2$ (with in-situ Zn reduction).

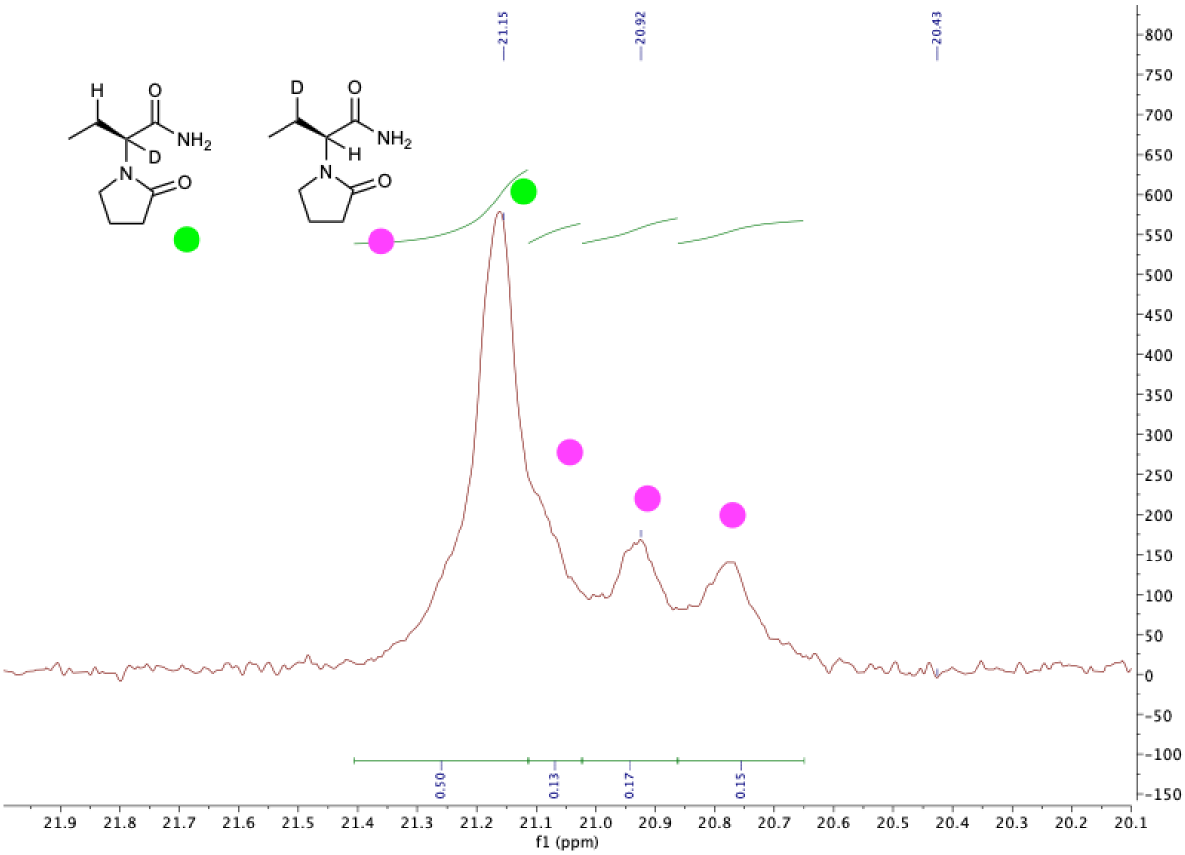

Figure S18. Section of the quantitative ^{13}C NMR of the product of reaction of DHL with HD catalyzed by ^{Ph}BPE-$CoCl_2$ (with in-situ Zn reduction) (rt, $CDCl_3$).

5. H_2/D_2 Scrambling

In a nitrogen filled glovebox, a J. Young NMR tube was charged with a C_6D_6 (0.5 mL) solution of (*R*,*R*)-(^{Ph}BPE)-Co-(COD) (0.010 g, 0.015 mmol) (tube 1). A second J. Young NMR tube was sealed but left empty (tube 2). The tubes were removed and taken to a high-vacuum line. The solution in tube 1 was frozen, and the headspace removed under vacuum. The tube was back-filled with 4 atm of H_2, and the solution was kept frozen. Tube 2 was similarly evacuated and backfilled with 4 atm of D_2. The two tubes were subsequently placed on a two-port, which was evacuated in the middle. The gasses of both tubes were allowed to mix for 10 minutes with the solution still frozen, after which tube 1 was sealed, thawed, and mixed. The contents were analyzed by ^{1}H NMR.

A)

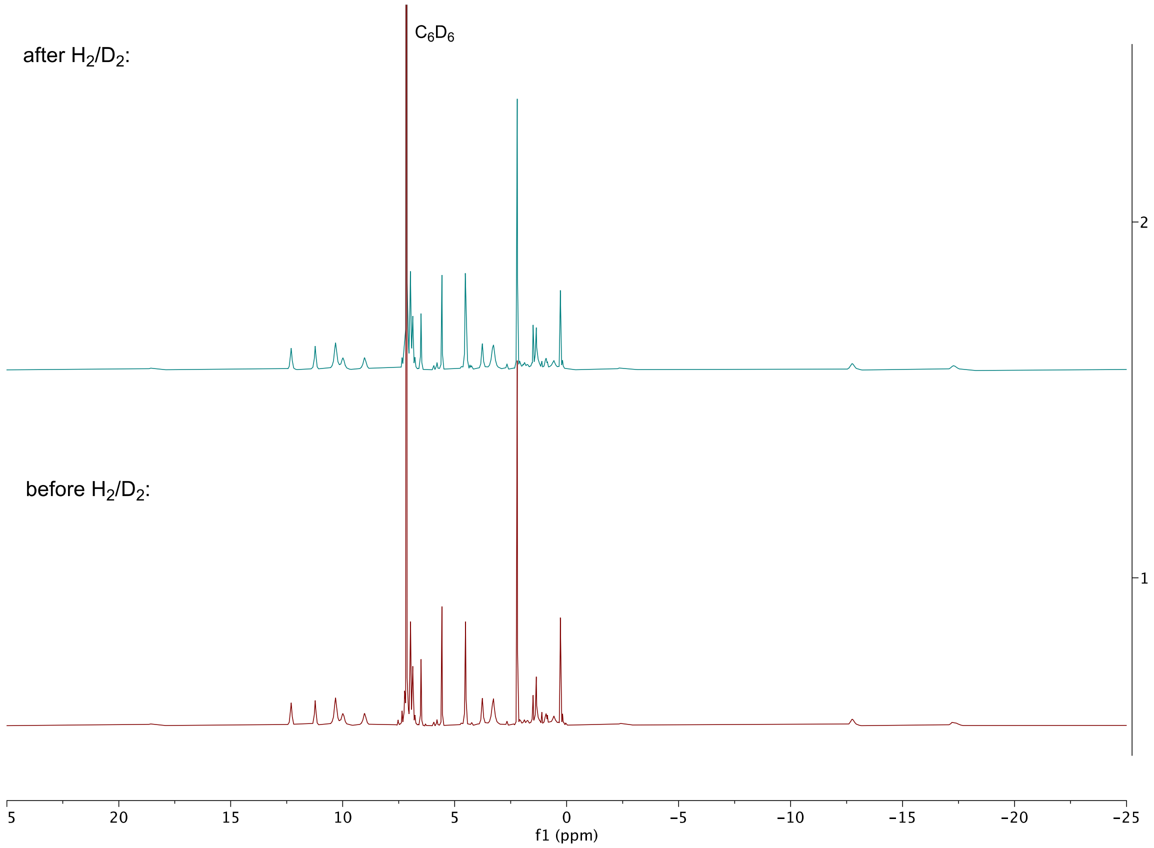

B)

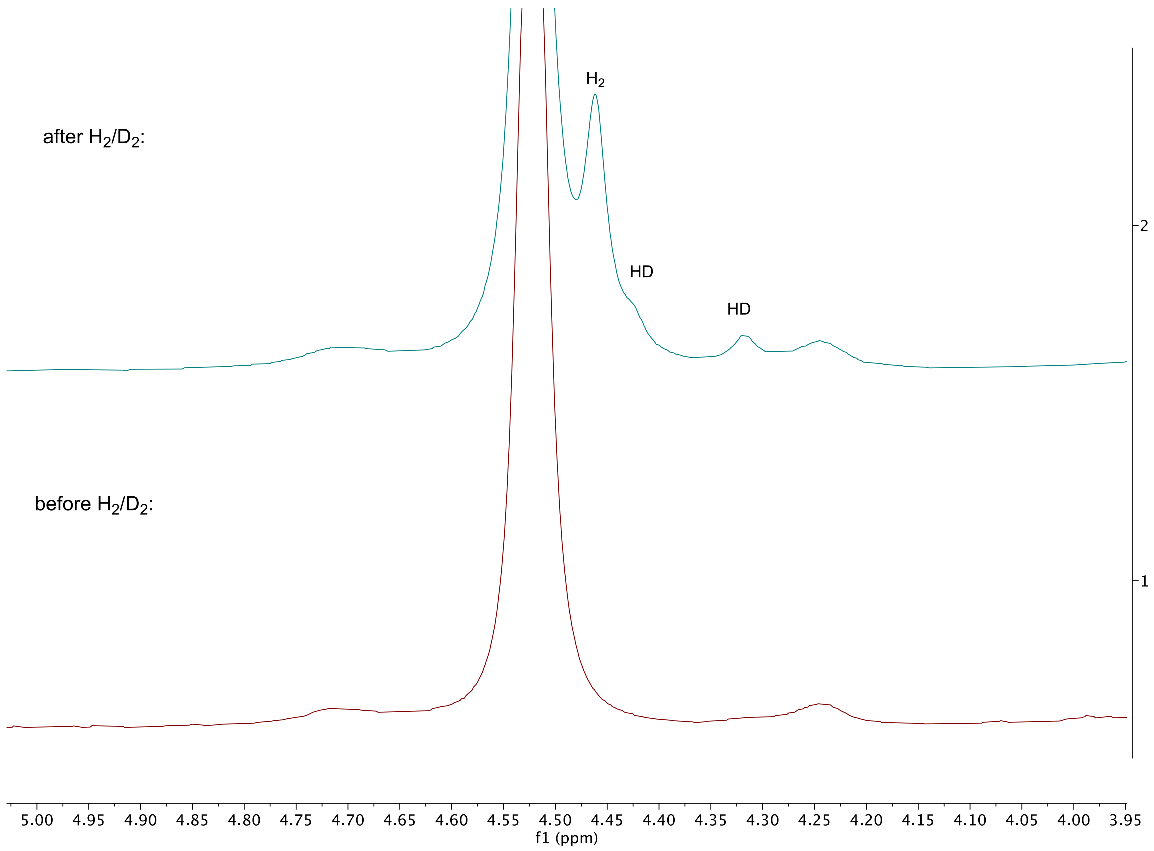

Figure S19. A)^{1}H NMR spectra of [Ph]BPE-Co-(COD) before and after addition of H_2 and D_2 (rt, C_6D_6). **B)** Section of ^{1}H NMR spectra of [Ph]BPE-Co-(COD) before and after addition of H_2 and D_2 (rt, C_6D_6).

6. Computational details

All calculations were performed with the Gaussian09 package, *Rev. D01*.[2] The DFT hybrid functional B3LYP[3,4] was employed with the Grimme empirical dispersion correction (D3).[5] Additional calculations were carried out with two different computational protocols, PBE0[6,7]-D3BJ[8] and ωB97XD[9], to check the robustness of the obtained results. The IEFPCM model was used in all calculation in order to include solvent effects (methanol solvent).[10-13] For the geometry optimizations, the small 6-311G (d,p)[14] (BS1) basis set was used on all non-metals, whereas the basis set and the pseudopotential LANL2TZ[15] was used on Co. In order to obtain more accurate energies, single point calculations were performed with 6-311++G(2df,2pd) on all non-metals, whereas the basis set and the pseudopotential LANL2TZ was used on Co (BS2).

Counter poise corrections[16,17] were computed at the BS2 level (CP_{BS2}) to correct for the artificial lowering of the electronic energy, caused by the borrowing of basis functions when molecular fragments are joined into one model. This correction was computed for the following steps: addition of H_2, MeOH, MAA or DHL to the model. For the addition of H_2, the CP corrections were computed at three levels of theory (CP = 0.9 kcal/mol for B3LYP-D3; CP = 0.9 kcal/mol for PBE0-D3BJ, CP = 0.8 kcal/mol for wB97XD). For the addition of MeOH, CP = 0.8 kcal/mol at the B3LYP-D3 level of theory. The CP corrections for the addition of MAA and DHL were 3.9 kcal/mol and 3.1 kcal/mol, respectively, at the B3LYP-D3 level of theory.

The computed free energies (ΔG°1atm, BS1) in the gas phase were converted into the corresponding 1M standard state energies, employing a standard state (SS) conversion term.[18] Only reactions where the number of moles are changed are affected. For the reaction A + B = C at 323.15 K, SS = -2.1 kcal/mol. For reactions involving explicit solvent, the standard state of the solvent is employed, which is 4.2 kcal/mol (based on the concentration of the pure solvent of 24.7 M for MeOH, derived from the density of 0.792 g/mL). Temperature corrections were included in all free energies to match the experimental temperature (50 °C). The standard state Gibbs free energies ($\Delta G°_{1M,323K}$) reported in the main text correspond to:

(eq.1) $\Delta G°_{1M,323K} = \Delta G_{1atm,\ 323K,BS1} - \Delta E_{1atm,BS1} + \Delta E_{1atm,BS2} + CP_{BS2} + SS_{323K}$

Enantiomeric excesses were evaluated from the computed barriers for the rate limiting steps using the following formula:[19]

(eq. 2) $e.e.(\%)_{theo} = \frac{1-e\left(-\frac{\Delta\Delta G\#}{RT}\right)}{1+e\left(-\frac{\Delta\Delta G\#}{RT}\right)} * 100$

7. Evaluation of the Co-Substrate interaction strength

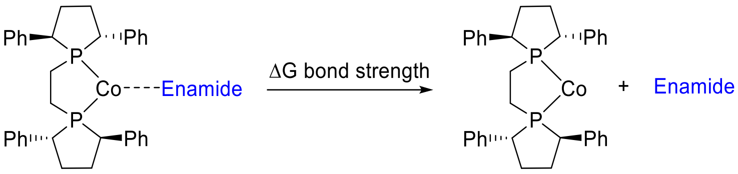

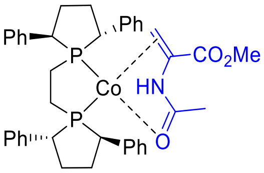

$\Delta G(323K) = 49.6$

(*R*,*R*)-^{Ph}BPE-Co-MAA

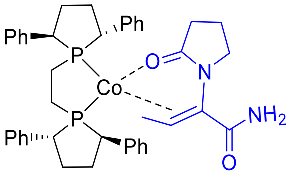

$\Delta G(323K) = 48.0$

(*R*,*R*)-^{Ph}BPE-Co-DHL

Figure S20. Evaluation of the Co-enamide interaction strength (B3LYP-D3[IEFPCM(methanol)], kcal/mol, 323 K).

8. Alternative mechanisms for the hydrogenation of *dehydro*-levetiracetam (DHL)

9. Redox Co(0)-Co(II) mechanism A for the hydrogenation of DHL

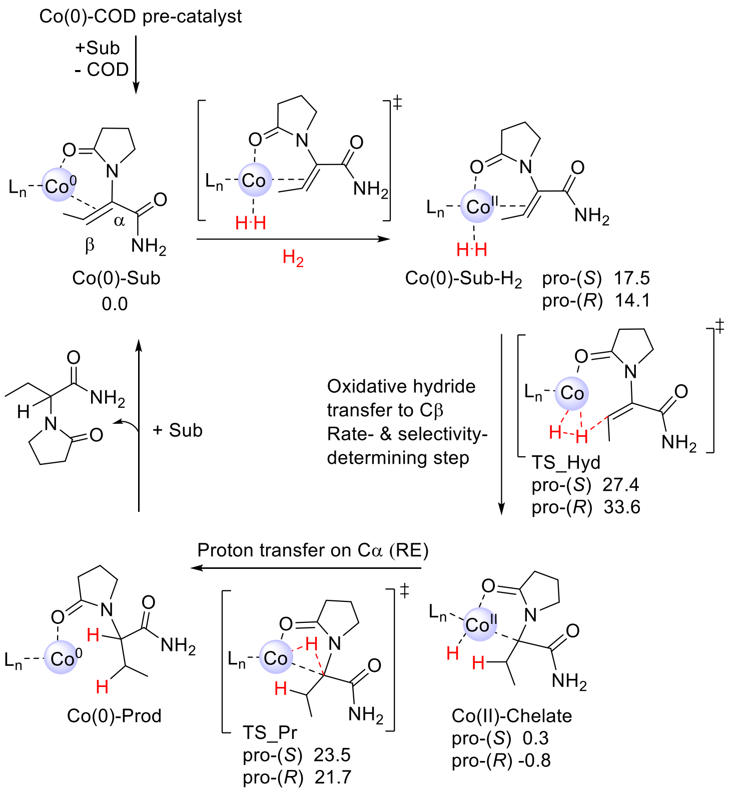

Figure S21. Mechanism and computed free energies (B3LYP-D3[IEFPCM(methanol)], kcal/mol, 323 K, relative to Co-Sub) for ^{Ph}BPE-Co-catalyzed hydrogenation of DHL via mechanism **A**, where oxidative hydride transfer occurs to the Cβ atom via TS-Hyd. This step is rate-limiting, with a barrier of 27.4 kcal/mol for the formation of the (*S*)-product. In the final step, reductive elimination occurs in order to liberate the product and regenerate the catalyst. This step has a barrier of 23.5 kcal/mol for the (*S*)-pathway, assuming that the (*R*) and (*S*) intermediates are not in equilibrium (due to the high backwards barrier), and 24.3 kcal/mol if they are assumed to be in equilibrium.

10. σ-bond metathesis mechanism B for the hydrogenation of DHL

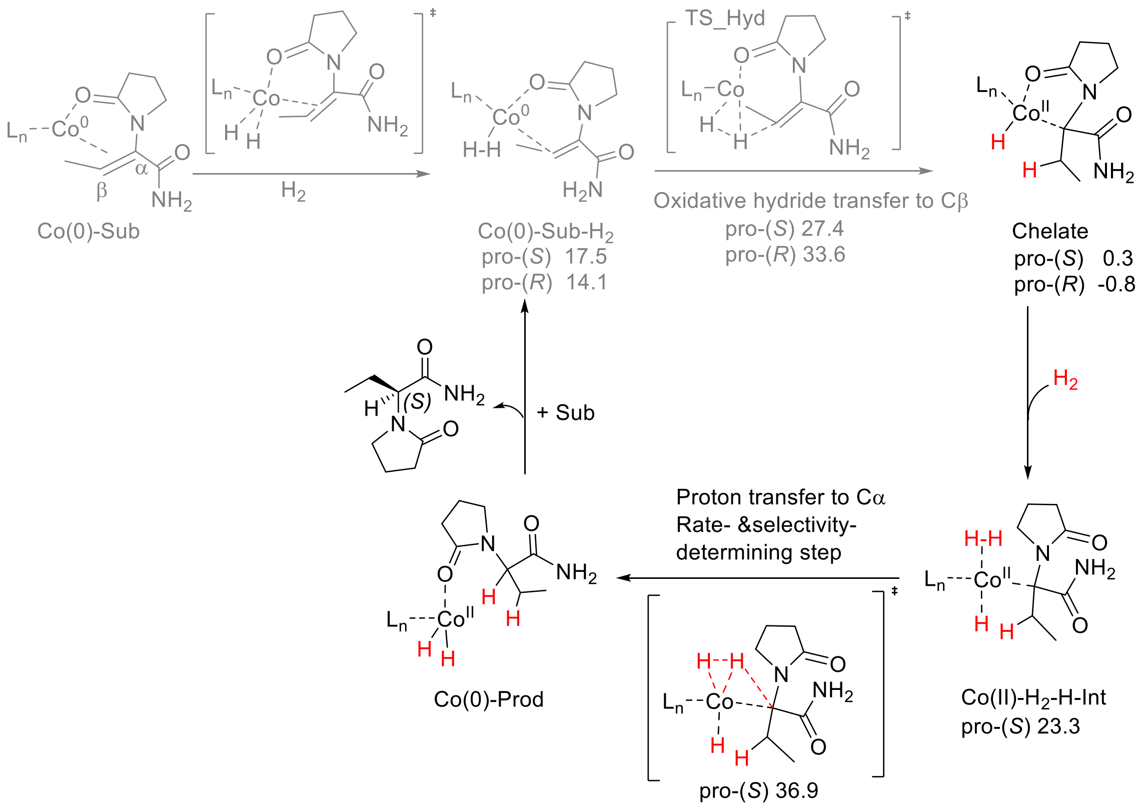

Figure S22. Mechanism and computed free energies (B3LYP-D3[IEFPCM(methanol)], kcal/mol, 323 K, relative to Co-Sub) for ^{Ph}BPE-Co-catalyzed hydrogenation of DHL via a σ-bond metathesis mechanism **B**. After formation of chelate intermediate, coordination of H_2 occurs, followed by a proton transfer to Cα, which is the rate- and selectivity-determining step. The overall barrier for formation of the (*S*)-product (relative to the lowest lying intermediate) is 37.7 kcal/mol.

11. Computed mechanism for the hydrogenation of DHL via mechanism C with a 4-membered metallacycle (mechanism C(m4))

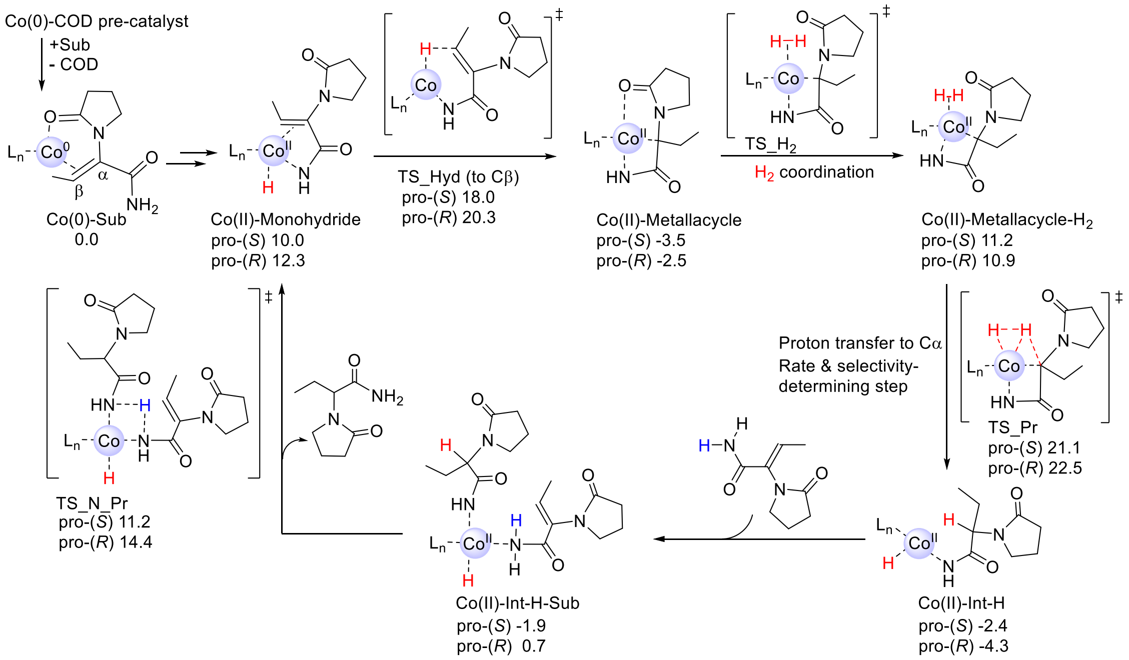

Figure S23. Mechanism and computed free energies (kcal/mol, 323 K, B3LYP-D3/BS2[IEFPCM]//B3LYP-D3/BS1[IEFPCM] level of theory) for the ^{Ph}BPE-Co-catalyzed hydrogenation of DHL via metallacycle mechanism **C** that will give a 4-membered metallacycle, including (*R*) and (*S*) pathways. Energies are given relative to Co(0)-Sub. Hydride transfer to the Cβ atom occurs first (computed barriers 18.0 kcal/mol and 20.3 kcal/mol, for pro-(*S*) and pro-(*R*) TSs, respectively). Then, H_2 coordination takes place, followed by proton transfer to the Cα atom, forming a Co(II)-Int-H intermediate. A proton transfer to the Cα atom is found to be rate-limiting, with the computed barriers of 24.6 kcal/mol and 26.0 kcal/mol for pro-(*S*) and pro-(*R*) TS structures. Finally, coordination of another substrate occurs, which transfers its proton to the nitrogen atom, resulting in the final product and the regeneration of the active Co(II)-monohydride species.

12. Mechanism C for the hydrogenation of DHL with hydride transfer to Cα and formation of a 5-membered metallacycle (mechanism C(m5))

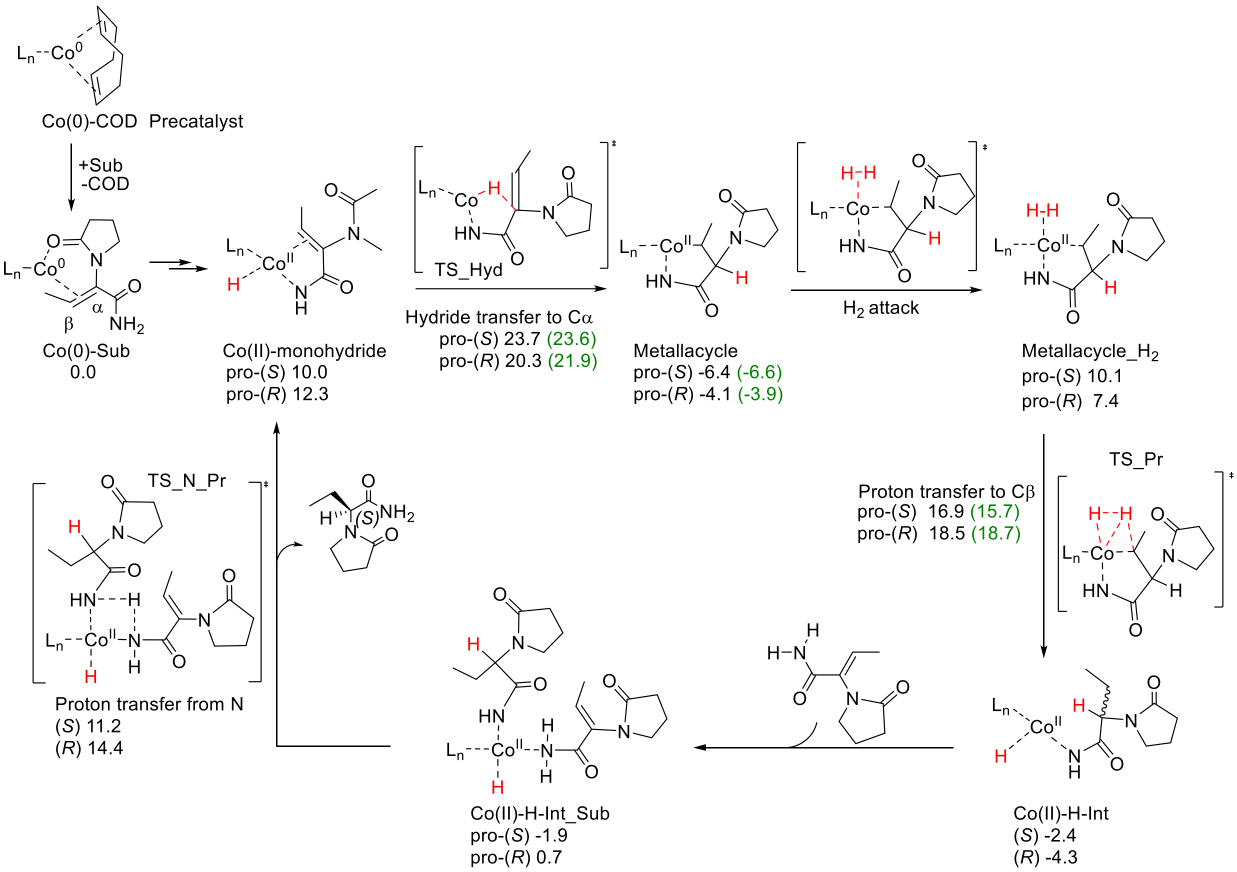

Figure S24. A) Mechanism and computed free energies (B3LYP-D3/[IEFPCM(methanol)], kcal/mol, 323 K, relative to Co-Sub) for ^{Ph}BPE- Co-catalyzed hydrogenation of DHL via a 5-membered metallacycle mechanism **C**(5m) where hydride transfer to the Cα atom occurs first. The barriers for this step are 20.3 kcal/mol and 23.7 kcal/mol, for pro-(*R*) and pro-(*S*) TSs, respectively. Then, H_2 coordination takes place, followed by proton transfer to the Cβ atom, forming a Co(II)-H-intermediate. The computed barriers for this step are 23.3 kcal/mol and 24.9 kcal/mol for pro-(*S*) and pro-(*R*) TS structures. Finally, coordination of another substrate occurs, which transfers its proton to the nitrogen atom, resulting in the final product and the regeneration of the active Co(II)-monohydride species. The rate-limiting step for the formation of (*S*)-product is hydride transfer to Cα (23.7 kcal/mol, with the subsequent proton transfer being close in energy, 23.3 kcal/mol), whereas for the formation of (*R*)-product, it is proton transfer to Cβ (24.9 kcal/mol, relative to the pro-(*S*)-Metallacycle). Energies in parenthesis with green color are from computational models including an explicit MeOH molecule hydrogen-bonded to the substrate. We note that all barriers are computed assuming Curtin-Hammett conditions, implying that intermediates along the (*R*)- and (*S*)-pathways can interconvert. There exist also the possibility that formation of the Co-monohydride or Co-metallacycle is not reversible (non-Curtin Hammett conditions), the analysis of the e.e. in that case is beyond the scope of this work.

13. Precatalytic pathways for *dehydro*-levetiracetam (DHL)

14. A direct oxidative addition of the ionizable group of the substrate to Co(0) giving Co(II)-monohydride

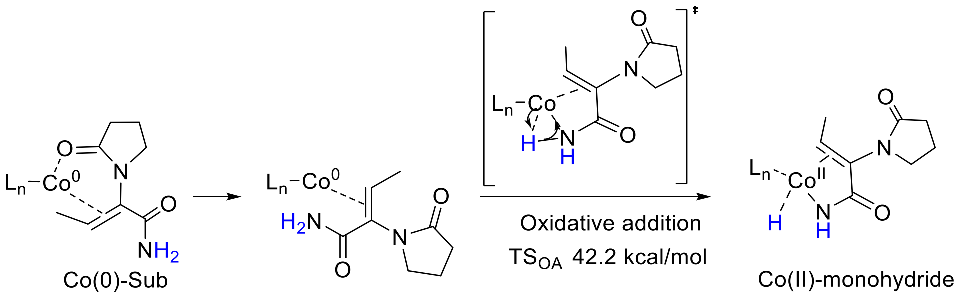

Figure S25. Direct oxidative addition of DHL to convert Co(0)-Sub to a Co(II)-monohydride intermediate via transition state TS_{OA} (B3LYP-D3/[IEFPCM(methanol)], kcal/mol, 323 K, relative to Co-Sub). The free energy barrier of 42.2 kcal/mol is given relative to Co(0)-Sub and is not feasible at the experimental temperature (323 K), indicating that the Co(II)-monohydride intermediate cannot be formed through direct oxidative addition. An alternative pathway is described in the main text (Scheme 3).

15. MeOH-mediated proton transfer from NH_2 of the Co(0)-DHL to give a metallacycle intermediate

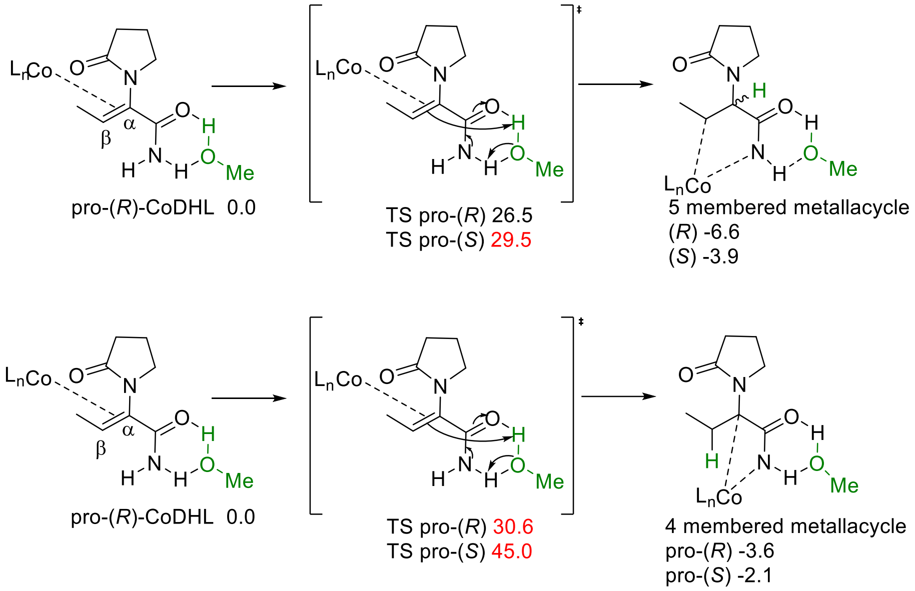

Figure S26. An alternative precatalytic pathway where a protic solvent MeOH mediates proton transfer from NH_2 of the Co(0)-enamide to either Cα or Cβ atoms of the enamide (B3LYP-D3[IEFPCM(methanol)], kcal/mol, 323 K, relative to Co-Sub). If the proton is transferred to Cα atom, a 5-membered metallacycle is formed with a feasible computed barrier of 26.5 kcal/mol. On the contrary, proton transfer to Cβ results in very high barriers.

16. Alternative mechanisms for the hydrogenation of methyl 2-acetamidoacrylate (MAA)

17. Mechanism A for MAA with hydride transfer to the Cα atom

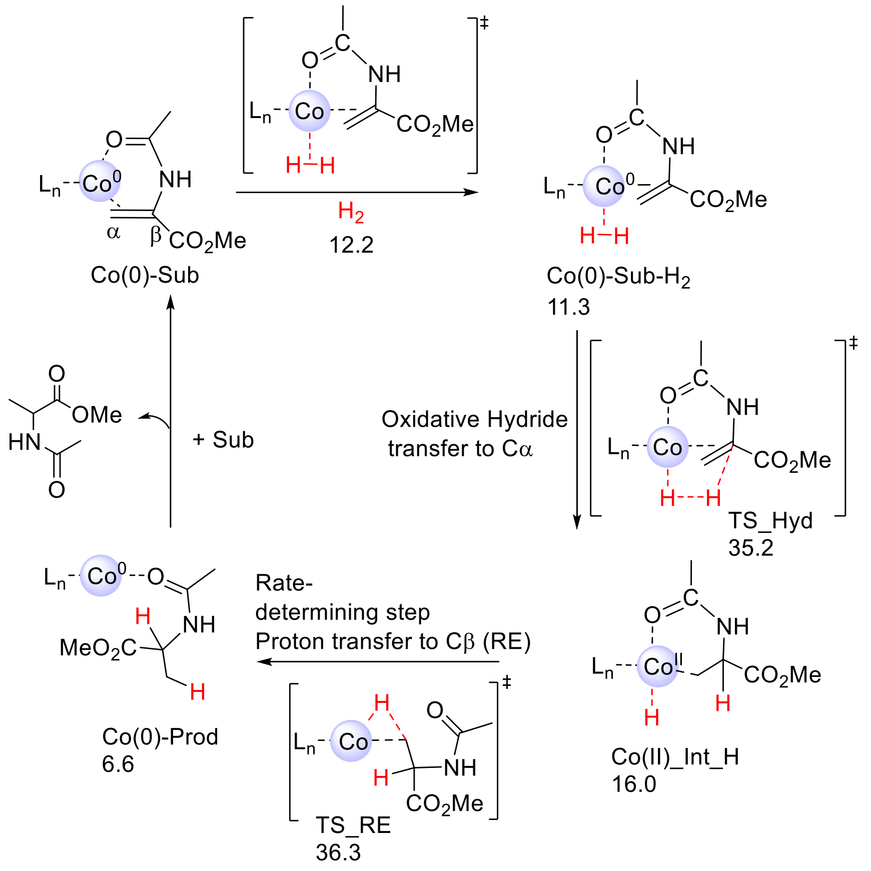

Figure S27. Mechanism and computed free energies (B3LYP-D3/[IEFPCM(methanol)], kcal/mol, 323 K, relative to Co-Sub) for ^{Ph}BPE-Co-catalyzed hydrogenation of MAA via <u>mechanism **A**</u> where oxidative hydride transfer occurs <u>to the Cα atom</u> via TS-Hyd (compared to Cβ in the main text). The rate limiting step the proton transfer to Cβ with a barrier of 36.3 kcal/mol. Evaluated is only the reaction pathway that will give the (*S*)-product.

18. σ-bond metathesis mechanism B for MAA; hydride transfer to Cβ

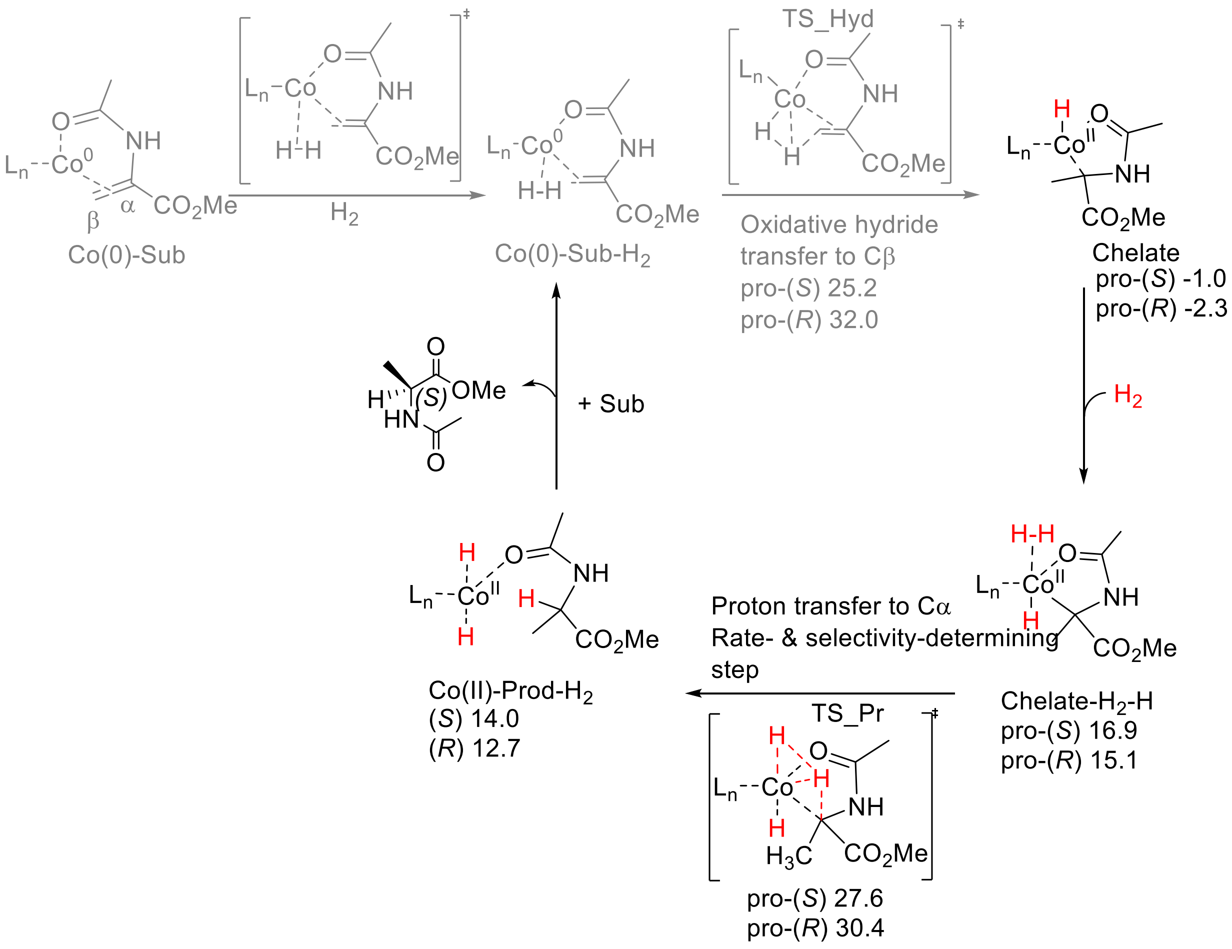

Figure S28. Mechanism and computed free energies (B3LYP-D3[IEFPCM(methanol)], kcal/mol, 323 K, relative to Co-Sub) for ^{Ph}BPE-Co-catalyzed hydrogenation of MAA via σ-bond metathesis mechanism **B**, in which after formation of chelate intermediate, coordination of H_2 occurs, followed by proton transfer to Cα, which the rate- and selectivity-determining step. The overall barrier for formation of the (*R*)-product is 32.7 kcal/mol and 28.6 kcal/mol for the (*S*)-product (for the latter assuming the chelate intermediates are not in equilibrium, if they are, the (*S*)-barrier increases to 29.9 kcal/mol).

19. 6-membered metallacycle mechanism C(6m) for MAA, hydride transfer to the Cα

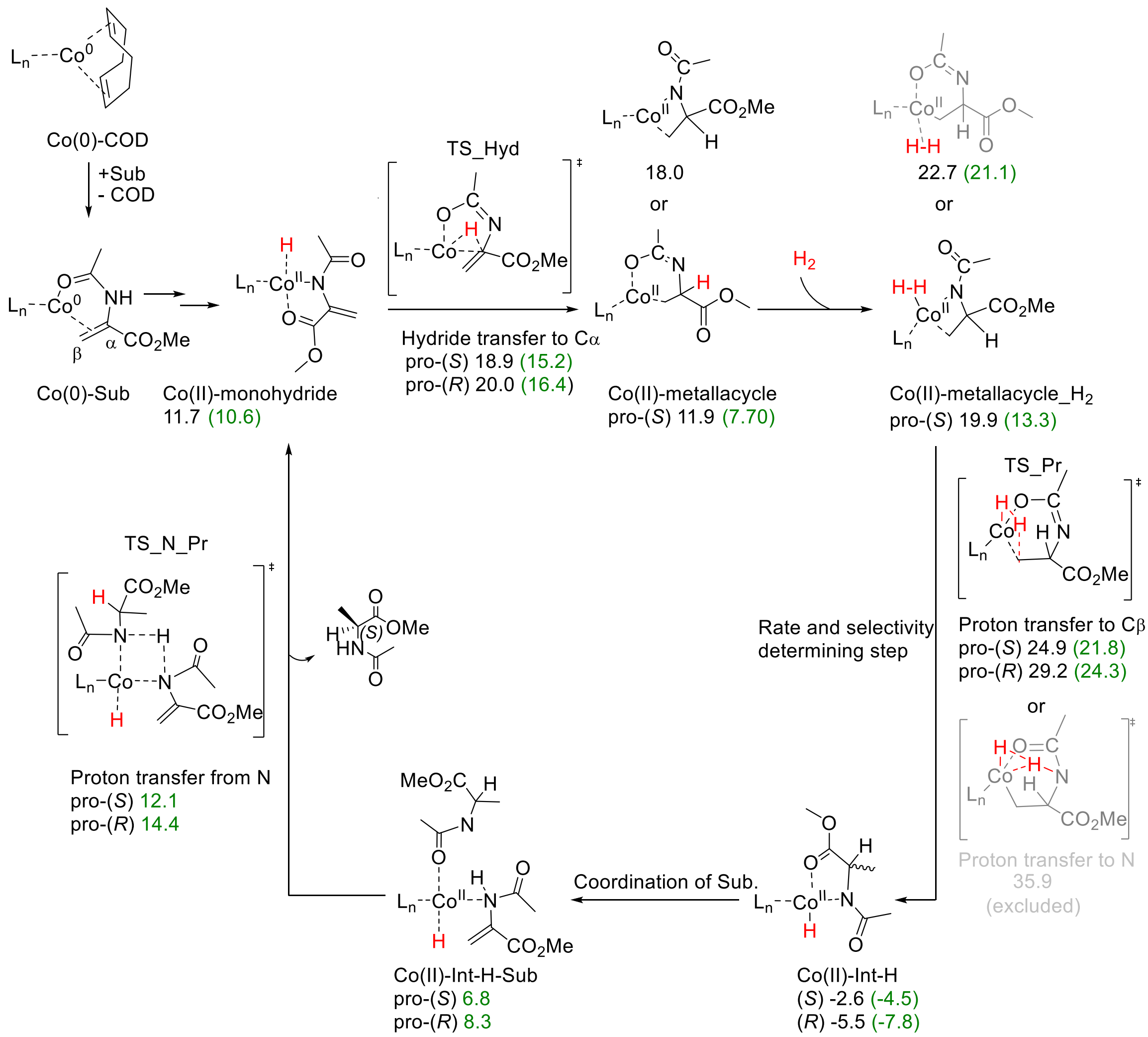

Figure S29. Mechanism and computed free energies (B3LYP-D3/[IEFPCM(methanol)], kcal/mol, 323 K, relative to Co-Sub) for ^{Ph}BPE-Co-catalyzed hydrogenation of MAA via mechanism **C**(6m) where hydride transfer occurs to the Cα atom. The barriers are 18.9 kcal/mol and 20.0 kcal/mol, for the pro-(*S*) and pro-(*R*) TSs. Note that the Co(II)-metallacycle and Co(II)-metallacycle-H_2 intermediate can adopt two conformations, where either the oxygen of the amido group or nitrogen of the amido group interacts with cobalt. In the next step, either proton transfer to N or to Cβ may occur. The barriers are 35.9 kcal/mol and 24.9 kcal/mol, respectively, indicating that proton transfer occurs to Cβ, which is found to be rate-limiting step. We also tested if addition of an explicit MeOH molecule that hydrogen bonds to the substrate changes the barriers of mechanism **C** (energies in parenthesis with green color are from computational models including an explicit MeOH molecule hydrogen-bonded to the substrate). In presence of explicit MeOH, the rate-limiting step is the proton transfer to Cβ, with barriers of 21.8 kcal/mol and 24.3 kcal/mol, for the pro-(*S*) and pro-(*R*) TSs, respectively.

20. C(imine) and D mechanisms for MAA

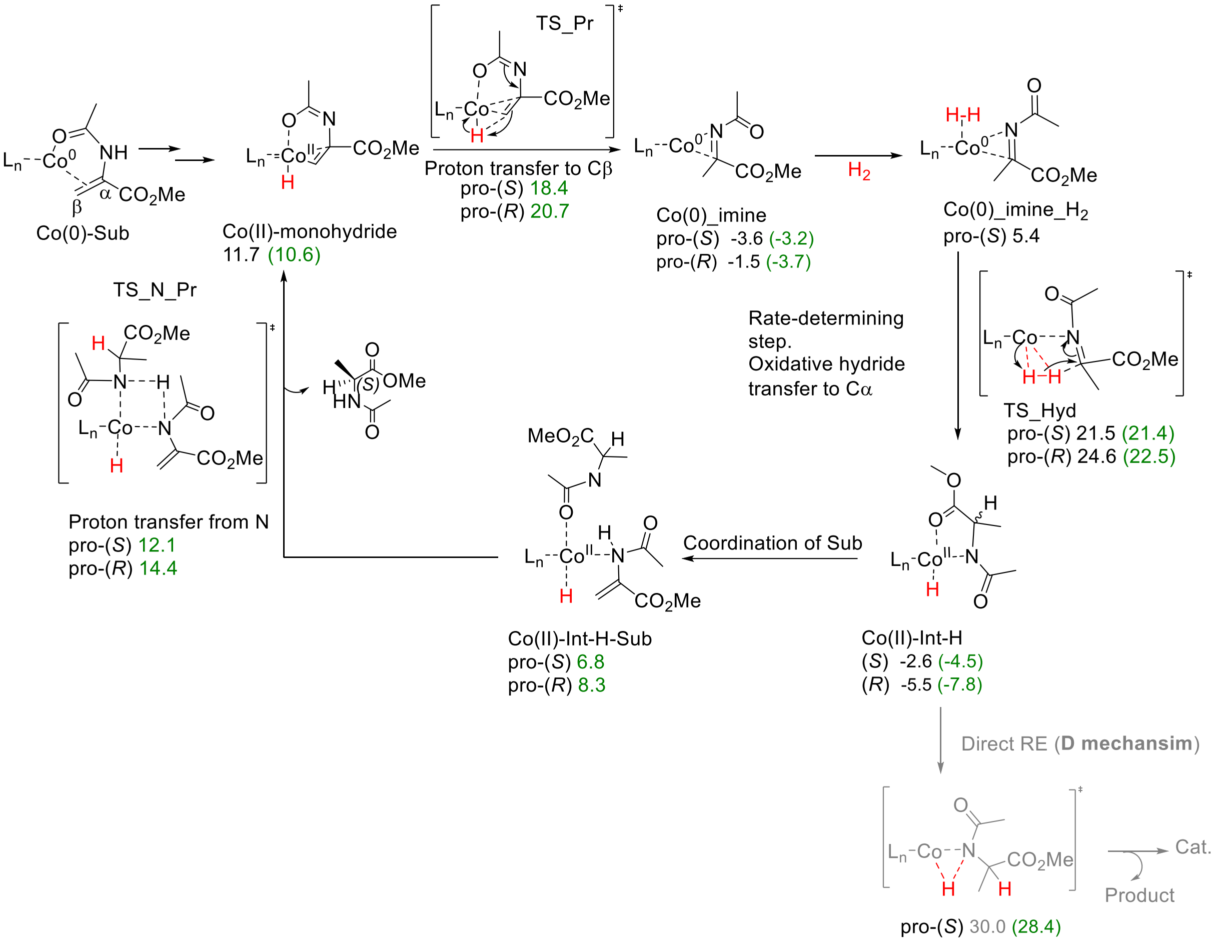

Figure S30. Mechanisms and computed free energies (B3LYP-D3/[IEFPCM(methanol)], kcal/mol, 323 K, relative to Co-Sub) for ^{Ph}BPE-Co-catalyzed hydrogenation of methyl 2-acetamidoacrylate (MAA) <u>via an imine mechanism in which enamine-imine tautomerization occurs</u>. After proton transfer to Cβ (barrier of 18.4 kcal/mol, with the explicit MeOH-Substrate interaction), H_2 coordination occurs. This is is followed by hydride transfer from H_2 to the Cα atom to form the Co(II)-Int-H. The barrier is 25.1 kcal/mol (*S*-product), with MeOH hydrogen bonded to the substrate. From the Co(II)-Int-H intermediate, the mechanism can proceed either through coordination of another substrate and transfer of a proton to N (Mechanism **C**(imine)), or a direct reductive elimination where a proton from Co-H is transferred to N (Mechanism **D**). The latter has a computed barrier of 33.7 kcal/mol (32.1 kcal/mol with MeOH) and is not feasible at 323K. Energies in parenthesis with green color are from computational models including an explicit MeOH molecule hydrogen-bonded to the substrate.

21. Alternative C(imine, heterolytic) mechanism for MAA with heterolytic H_2 cleavage

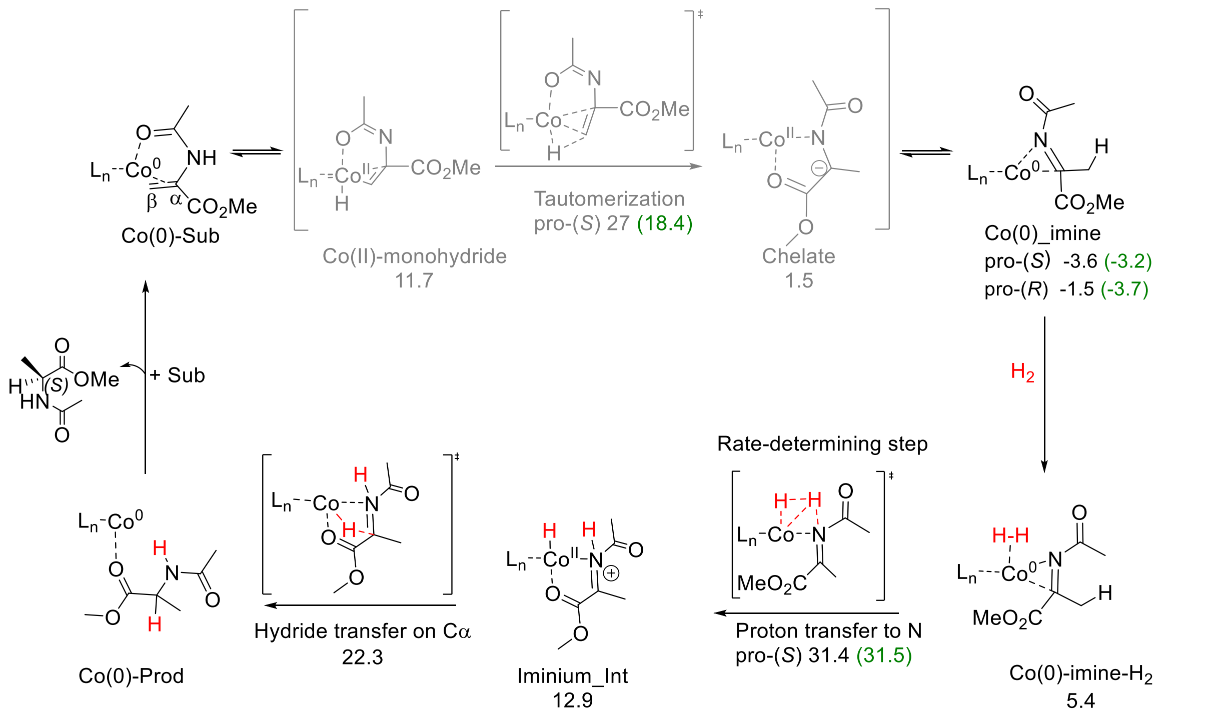

Figure S31. Mechanism and computed free energies (B3LYP-D3/[IEFPCM(methanol)], kcal/mol, 323 K, relative to Co-Sub) for ^{Ph}BPE-Co-catalyzed hydrogenation MAA via an alternative **C**(imine, heterolytic) mechanism, where after H_2 coordination, proton transfer from H_2 to the N atom takes place to form an iminium intermediated. This step is found to be rate-limiting with an overall barrier of 35.0 kcal/mol (computed relative to the Co-imine intermediate). After proton transfer to the N atom, hydride transfer to the Cα atom occurs in order to liberate the product. The overall barrier with the explicit MeOH-substrate interaction is 35.2 kcal/mol, which is consideren not feasible. Energies in parenthesis with green color are from computational models including an explicit MeOH molecule hydrogen-bonded to the substrate.

22. Precatalytic pathways for methyl 2-acetamidoacrylate (MAA)

23. A direct oxidative addition of the ionizable group of MAA to Co(0), giving Co(II)-monohydride in the presence of explicit solvent

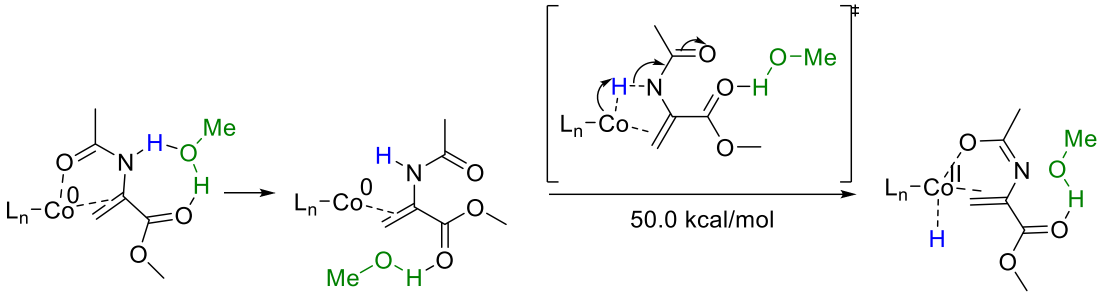

Figure S32. Direct oxidative addition of MAA to convert Co(0)-Sub to a Co(II)-monohydride intermediate via transition state TS_{OA} (B3LYP-D3[IEFPCM(methanol)], kcal/mol, 323 K, relative to Co-Sub). The free energy barrier of 50.0 kcal/mol is given relative to Co(0)-Sub and is not feasible at the experimental temperature (323 K), indicating that the Co(II)-monohydride intermediate cannot be formed through direct oxidative addition.

24. Proposed mechanism for the formation of the active Co(II)-metallacycle_H_2 species via an imine intermediate

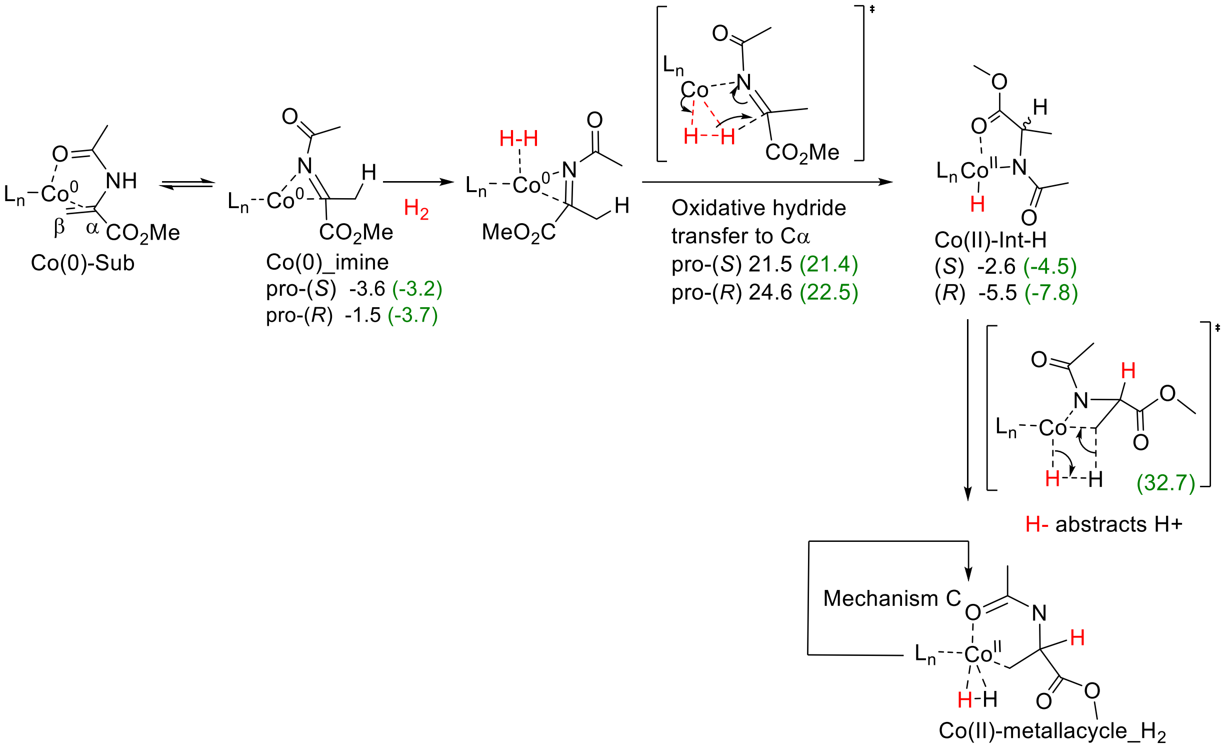

Figure S33. An investigated mechanism for the formation of an active catalyst species via an imine intermediate (B3LYP-D3[IEFPCM(methanol)], kcal/mol, 323 K, relative to Co-Sub). In the next step H_2 binds, followed by hydride transfer to the Cα atom. After formation of Co(II)-Int-H, a hydride may abstract a proton of the methyl group. This mechanism is excluded due to a very high barrier of 40.5 kcal/mol. Energies in parenthesis with green color are from computational models including an explicit MeOH molecule hydrogen-bonded to the substrate.

25. MeOH mediates proton transfer from NH_2 of the Co(0)-enamide giving either 6-mem. metallacycle or Imine intermediates

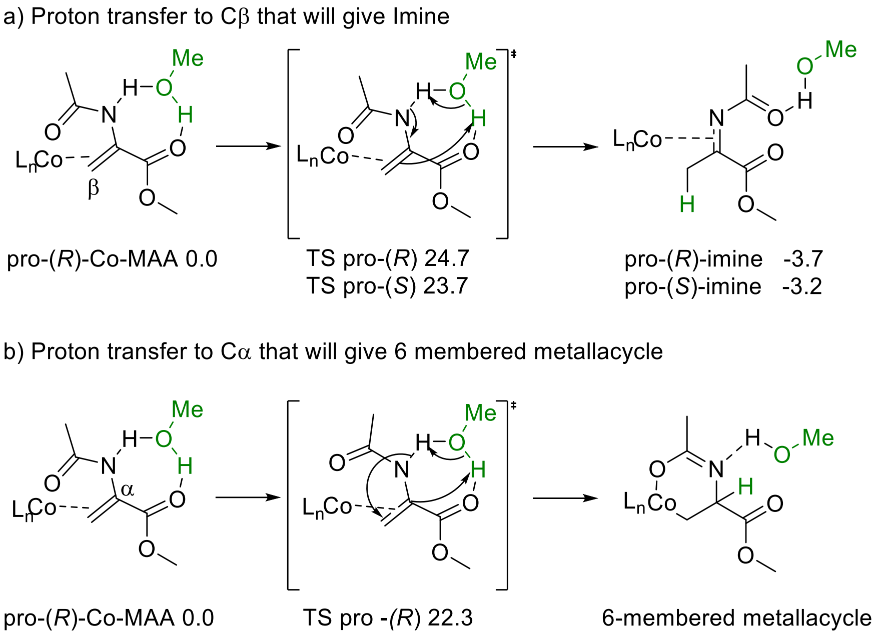

Figure S34. An alternative precatalytic pathway where a protic solvent MeOH mediates proton transfer from NH_2 of the Co(0)-enamide to either Cβ (a) or Cα (b) atoms of MAA (B3LYP-D3[IEFPCM(methanol)], kcal/mol, 323 K, relative to Co-Sub). Proton transfer to Cβ (a) can give either pro-(*R*) or pro-(*S*) imine intermediates. Our calculations show that both proton transfers might occur with barriers of 24.7 kcal/mol and 23.7 kcal/mol for the formation of pro-(*R*) or pro-(*S*) imine, respectively. Proton transfer to Cα giving a 6-membered metallacycle (b) is also feasible with the barrier of 22.3 kcal/mol.

26. H_2-assisted proton transfer from NH of the Co(0)-MAA giving Co-metallacycle-H_2

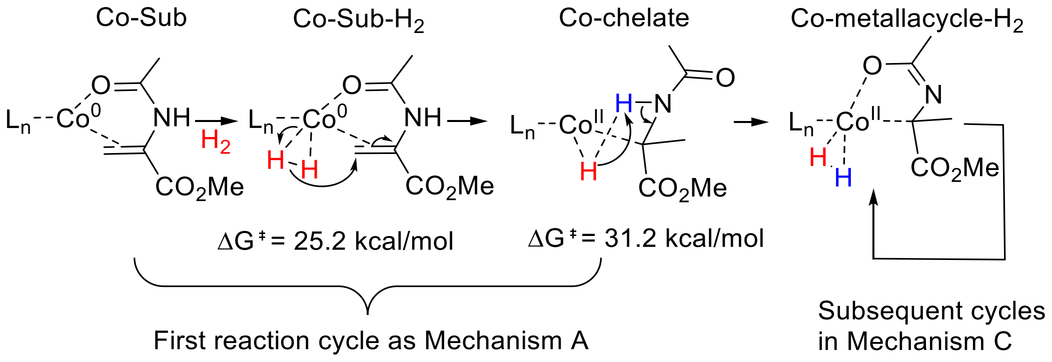

Figure S35. Pathway for formation of an active Co(II)-monohydride species, starting from a Co(0)-MAA, which binds H_2 and undergoes a hydride transfer to the enamide (B3LYP-D3[IEFPCM(methanol)], kcal/mol, 323 K, relative to Co-Sub), as in Mechanism **A**. The formed hydride may then abstract a proton from the ionizable NH group of the substrate, resulting in formation of the metallacycle that is part of Mechanism **C**. The barrier from Co(0)-Sub to the metallacycle is 31.2 kcal/mol, making it not feasible at the reaction temperature of 323K.

27. Mechanisms for methyl 2-acetamidoacrylate (MAA) and *dehydro*-levetiracetam (DHL) with MeOH

28. Mechanism A for MAA with explicit MeOH

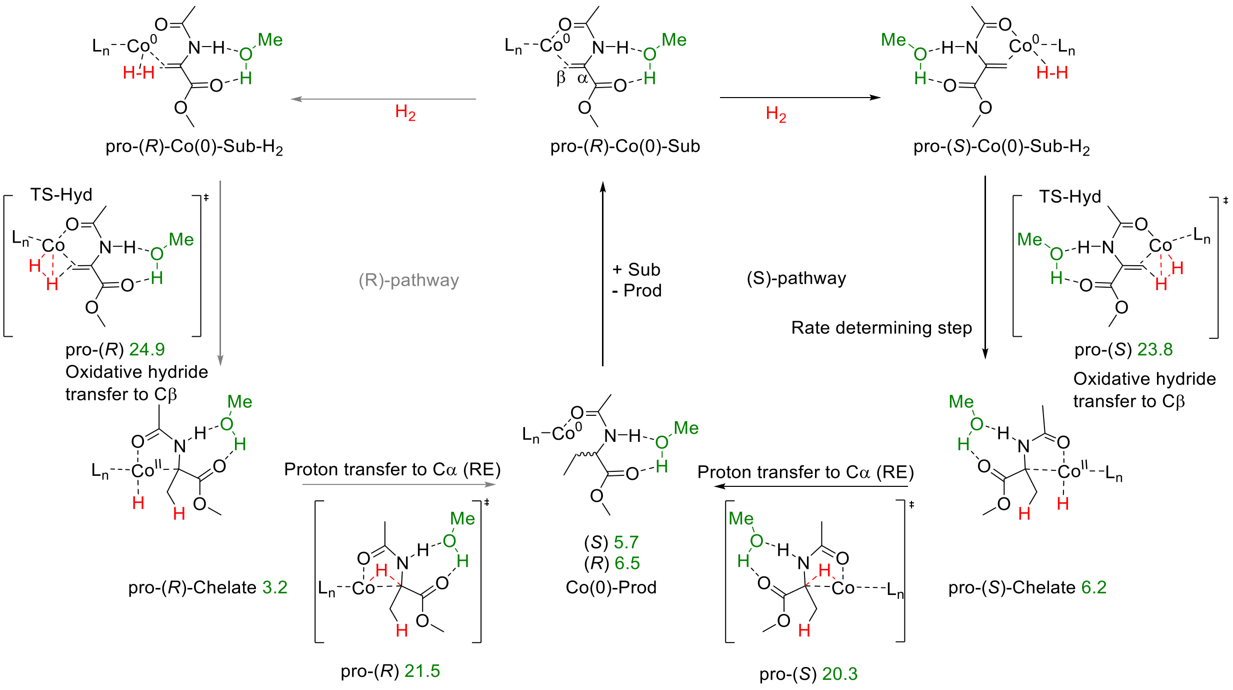

Figure S36. Mechanism and computed free energies (B3LYP-D3[IEFPCM(methanol)], kcal/mol, 323 K, relative to Co-Sub) for ^{Ph}BPE-Co-catalyzed hydrogenation of methyl 2-acetamidoacrylate (MAA) via an alternative mechanism **A**, with MeOH as a hydrogen bond donor to the substrate. Evaluated are both pro-(*S*) and pro-(*R*) reaction pathways. In the first step, oxidative hydride transfer occurs to the Cβ atom via pro-(*S*) and pro-(*R*) TS-Hyd structures to from the corresponding chelate intermediates. In the presence of MeOH (energies are given with green color), the barrier for the pro-(*S*) TS-Hyd is 23.8 kcal/mol, whereas the barrier for the pro-(*R*) TS-Hyd is 24.9 kcal/mol. This step is found to be rate-limiting. In the next step, the reductive elimination occurs through pro-(*S*) and pro-(*R*) TS_Pr structures with the barriers of 20.3 kcal/mol and 21.5 kcal/mol, respectively.

29. Rate-limiting TSs (Hyd transfer) via mechanism A for DHL with explicit MeOH

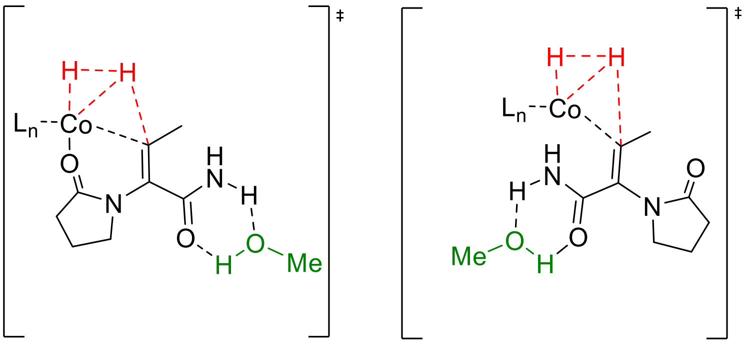

pro-(*S*) TS $\Delta G^{\ddagger}$ =27.4
pro-(*S*) TS_MeOH $\Delta G^{\ddagger}$ =26.3

pro-(*R*) TS $\Delta G^{\ddagger}$ =33.6
pro-(*R*) TS_MeOH $\Delta G^{\ddagger}$ =31.8

Figure S37. The optimized pro-(*S*) and (*R*)-TSs for the hydride transfer step and free energies (B3LYP-D3[IEFPCM(methanol)], kcal/mol, 323 K, relative to Co-Sub) for the ^{Ph}BPE-Co-catalyzed hydrogenation of DHL, Mechanism **A**, with and without an explicit MeOH-substrate interaction. The barriers are given relative to the reactant with or without explicit MeOH-substrate interaction, respectively. The barrier decrease is 1.1 kcal/mol for the pro-(*S*) TS whereas the decrease of the pro-(*R*) TS is 1.8 kcal/mol.

30. Metallacycle mechanism C (via a 4-membered metallacycle intermediate) for DHL with explicit MeOH

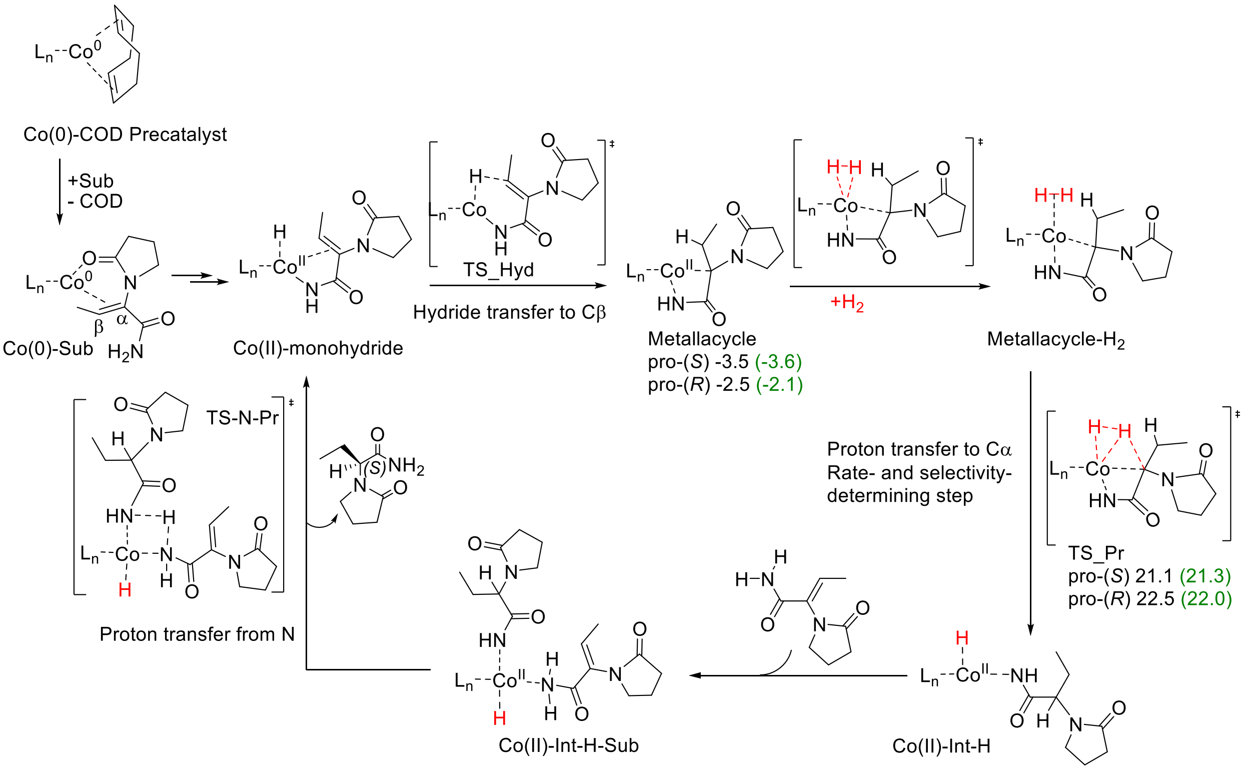

Figure S38. Proposed non-redox metallacycle mechanism **C**(4m) (via a four-membered metallacycle intermediate) and free energies (B3LYP-D3[IEFPCM(methanol)], kcal/mol, 323 K, relative to Co-Sub) for the ^{Ph}BPE-Co-catalyzed hydrogenation of DHL, with computed energies in presence of an explicit MeOH molecule given in green.

31. An alternative metallacycle mechanism C (4m) for DHL where MeOH delivers a proton to the nitrogen of the product

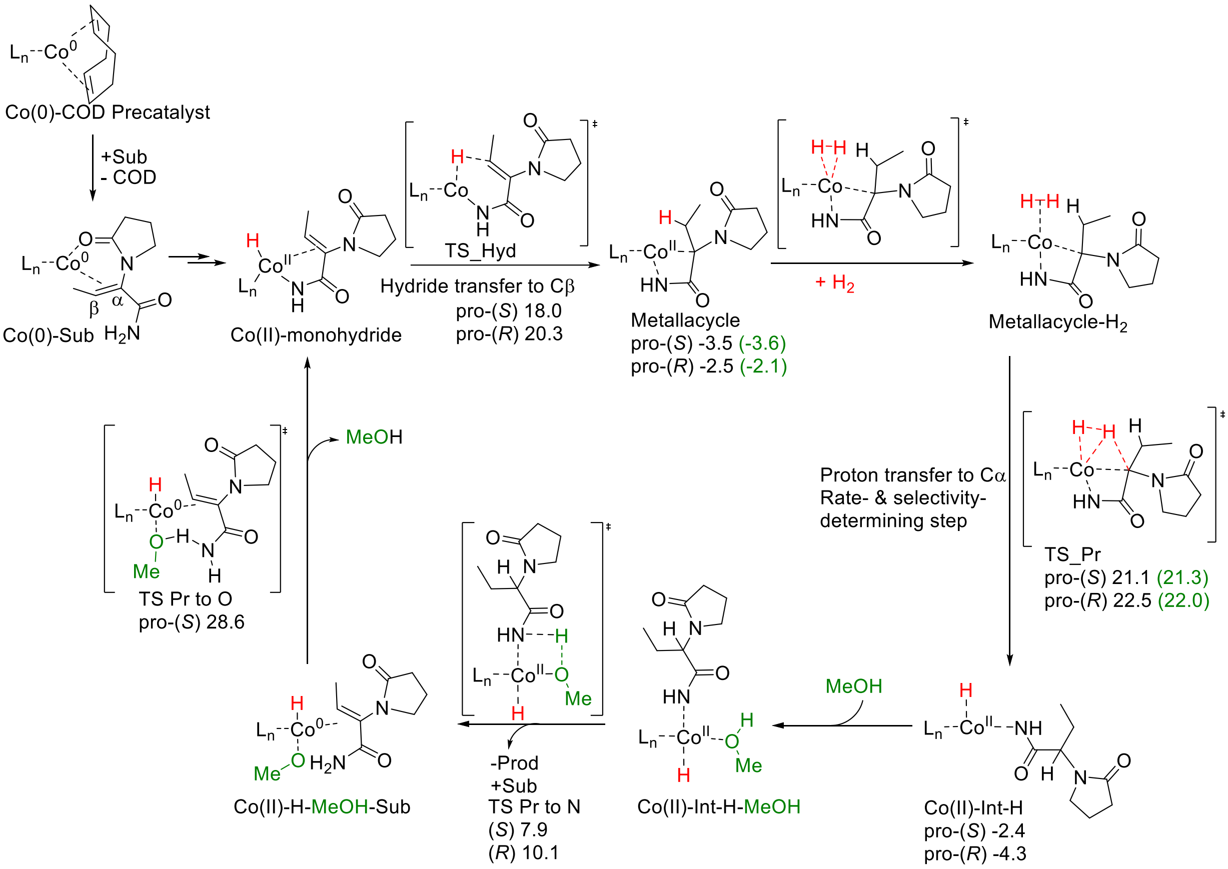

Figure S39. Metallacycle mechanism **C**(4m) (via a four-membered metallacycle intermediate) and free energies (B3LYP-D3[IEFPCM(methanol)], kcal/mol, 323 K, relative to Co-Sub) for the PhBPE-Co-catalyzed hydrogenation of DHL, with computed energies in presence of an explicit MeOH molecule given in green. Here, it is proposed that instead of substrate coordination step, MeOH coordinates first and delivers its proton to the nitrogen of the product. The computed barriers are feasible (12.2 kcal/mol for *S*-product, 14.4 kcal/mol for *R*-product). In the last step, in order to deprotonate the methoxy intermediate and reform Co(II)-monohydride one more substrate has to coordinate to the Co. This step is found to be rate limiting with the overall barrier of 32.9 kcal/mol, excluding this mechanism.

32. Alternative mechanism A for MAA with one MeOH molecule coordinated to Co

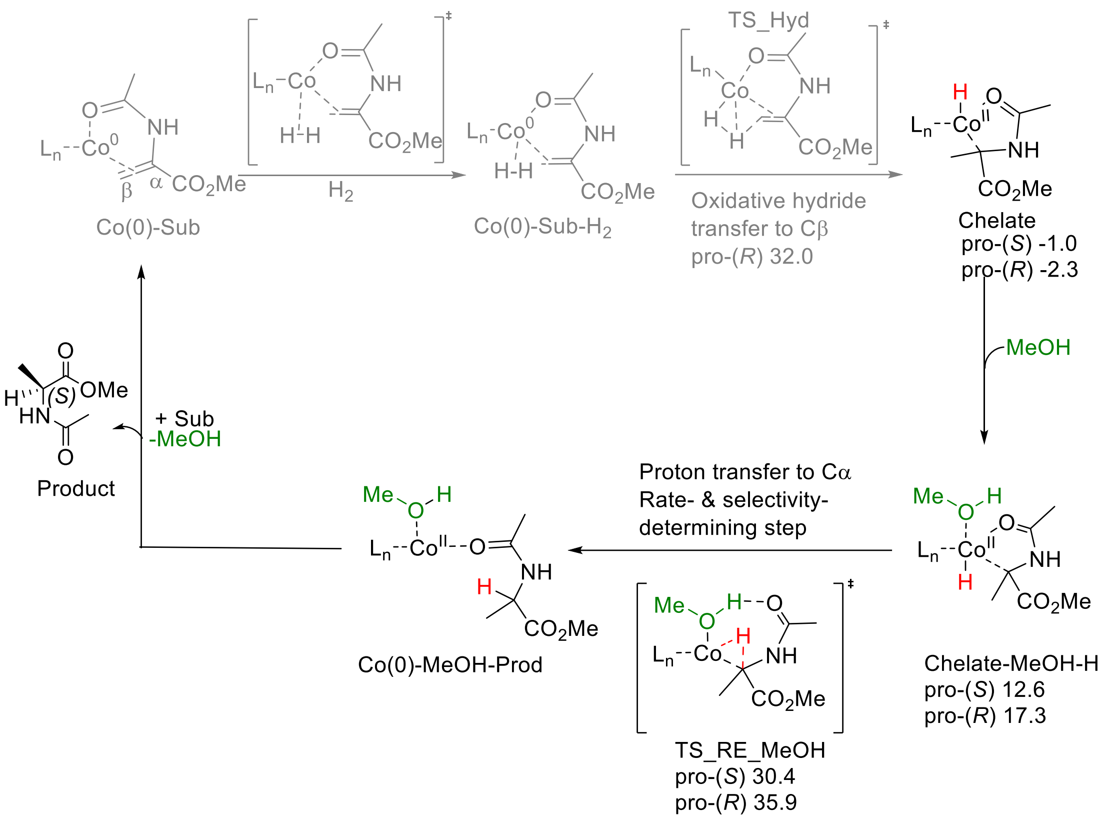

Figure S40. Mechanism and computed free energies (B3LYP-D3[IEFPCM(methanol)], kcal/mol, 323 K, relative to Co-Sub) for ^{Ph}BPE-Co-catalyzed hydrogenation of methyl 2-acetamidoacrylate (MAA) via an alternative MeOH-assisted mechanism **A** in which after formation of chelate intermediate, coordination of MeOH occurs. In the next step, reductive elimination takes place, while MeOH is coordinated to the metal centre. The overall barrier for the formation of (*S*)-product is 31.4 kcal/mol and for the (*R*)-product 38.2 kcal/mol, which are not feasible. The barriers were computed assuming that the (*R*) and (*S*) intermediates are not in equilibrium (due to the high backwards barrier).

33. Alternative mechanism for MAA with MeOH as proton donor

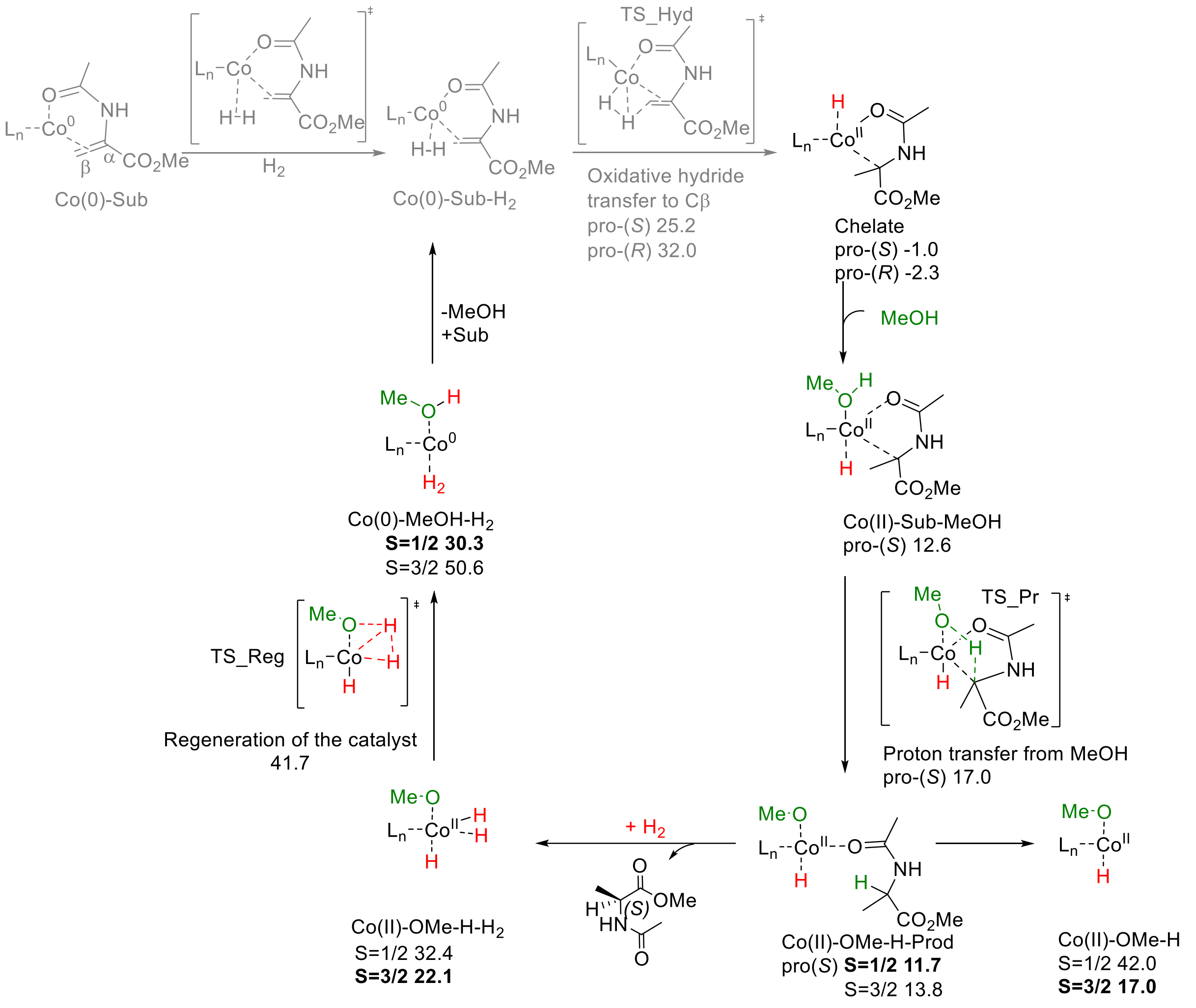

Figure S41. Mechanism and computed free energies (B3LYP-D3[IEFPCM(methanol)], kcal/mol, 323 K, relative to Co-Sub) for ^{Ph}BPE-Co-catalyzed hydrogenation of MAA via an alternative MeOH mechanism, in which after formation of the chelate intermediate, coordination of MeOH occurs. In the next step, methanol transfers its proton on Cα atom with reasonable barrier of 19.2 kcal/mol via pro-(*S*) TS_Pr (assuming that the (*R*) and (*S*) intermediates are not in equilibrium, due to the high backwards barrier). In order to regenerate the catalyst, coordination of one more H_2 molecule may occur, followed by proton transfer from H_2 to the methoxy group. The overall barrier is 45.8 kcal/mol, which is not feasible. The Co(II)-OMe-H and Co(II)-OMe-H-H_2 intermediates favor a quartet (S=3/2) spin state, which could open a possibility for side reactions.

34. The computed barriers and enantiomeric excesses for DHL and MAA

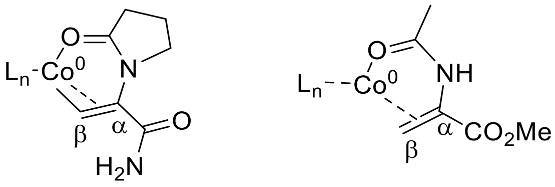

Table S1. The overall barriers for the ^{Ph}BPE-Co-catalyzed hydrogenation of DHL, without (left) and with (right) explicit MeOH-Substrate interactions (B3LYP-D3[IEFPCM(methanol)], kcal/mol, 323 K, relative to Co-Sub), via Mechanism **A** (**Figure S21**) and **C**(5m) (**Figure S24**).

TS	Mechanism C(5m)	Mechanism C(5m) (MeOH)
Pro-(*S*) (Hydride transfer to Cα)	23.7	23.6
Pro-(*R*) (Proton transfer to Cβ)	24.9	25.3
Computed e.e.	73.3 % (*S*)	86.8 (*S*)
TS	Mechanism A	Mechanism A (MeOH)
Pro-(*S*) (Hydride transfer to Cβ)	27.4	26.3
Pro-(*R*) (Hydride transfer to Cβ)	33.6	31.8
Computed e.e.	99.9 % (*S*)	99.9 % (*S*)

Table S2. The overall barriers for the ^{Ph}BPE-Co-catalyzed hydrogenation of MAA, without (left) and with (right) explicit MeOH-Substrate interactions (B3LYP-D3[IEFPCM(methanol)], kcal/mol, 323 K, relative to Co-Sub), via Mechanism **A** (**Figures 5, S36**) and via mechanism C(6m) (**Figure S29**).

Rate-limiting TS	Mechanism A	Mechanism A (MeOH)
Pro-(*S*) (Hydride transfer to Cβ)	25.2	23.8
Pro-(*R*) (Hydride transfer to Cβ)	32.0	24.9
Computed e.e.	99.9 % (*S*)	69.4%(*S*)
	Mechanism C(6m)	Mechanism C(6m) (MeOH)
Pro-(*S*) (Proton transfer to Cβ)	24.9	21.8
Pro-(*R*) (Proton transfer to Cβ)	29.2	24.3
Computed e.e.	99.9 % (*S*)	96.0 % (*S*)

35. Additional computational methods (PBE0-D3BJ and ωB97XD) with ^{Ph}BPE ligand for DHL and MAA

Table S3. Additional computational methods (PBE0-D3BJ and ωB97XD) with ^{Ph}BPE ligand for DHL and MAA. For MAA, PBE0-D3BJ and ωB97XD gave feasible barriers for rate-limiting TSs where (*S*) pathway is preferred via redox and C(6m) mechansim. A similar conclusion can be made for DHL, where additional methods gave feasible barriers (at reaction temperature of 323K) for Mechanisms A and C(5m). Energies are for models with one explicit MeOH.

Substrates	Mechanism	B3LYP-D3	PBE0-D3BJ	ωB97XD
Methyl 2-acetamidoacrylate (MAA)<br><br>Experimental *e.e.*: 85 % (*S*)	**A**	23.8 (*S*),<br>24.9 (*R*)	19.5 (*S*),<br>21.8 (*R*)	21.1 (*S*),<br>21.8 (*R*)
	Comp. *e.e.* (%)	69.4 (*S*)	94.6 (*S*)	49.7 (*S*)
	**C**(6m)	21.8 (*S*),<br>24.3 (*R*)	20.4 (*S*),<br>22.4 (*R*)	21.2 (*S*),<br>23.6 (*R*)
	Comp. *e.e.* (%)	96.0 (*S*)	91.5 (*S*)	95.3 (*S*)
	**C**(imine)	24.2 (*S*),<br>26.2 (*R*)	21.4 (*S*),<br>22.2 (*R*)	22.6 (*S*),<br>23.8 (*R*)
	Comp. *e.e.* (%)	91.5 (*S*)	55.3 (*S*)	73.3 (*S*)
Dehydro-levetiracetam (DHL)<br>Experimental *e.e.*: 97 % (*S*)	**A**	26.3 (*S*),<br>31.8 (*R*)	22.3 (*S*),<br>26.3 (*R*)	22.8 (*S*),<br>25.7(*R*)
	Comp. e.e. (%)	99.9 (S)	99.6 (S)	97.8 (S)
	**C**(5m)	23.6 (*S*),<br>25.3 (*R*)	24.1 (*S*),<br>27.8 (*R*)[b]	22.4 (*S*),<br>24.9 (*R*)
	Comp. *e.e.* (%)	86.8 (*S*)	99.4 (*S*)	96.0 (*S*)
	**C**(4m)	24.9 (*S*),<br>25.6 (*R*)	21.8 (*S*),<br>22.7 (*R*)	22.3 (*S*),<br>24.1 (*R*)
	Comp. *e.e.* (%)	49.7 (*S*)	60.5 (*S*)	88.6 (*S*)

36. Evaluation of quartet spin states

Table S4. Comparison of doublet and quartet spin states for critical structures, including the energetic reference state Co-enamide and the rate-limiting transition states of Mechanism **A** and **C**(6m) for MAA (free energies in kcal/mol at 323 K, B3LYP-D3, with SS and CP corrections, geometries without explicit MeOH). The quartet states are between 11.8 and 14.7 kcal/mol higher than the corresponding doublet states, making it highly unlikely that quartet states play a role in the catalytic cycle.

**Substrate**	**Species**	**Doublet (S = ½)**	**Quartet (S = 3/2)**
Methyl 2-acetamidoacrylate (MAA)	Co-MAA	0.0	11.8
	TS_Hyd (Mech. A)[a]	25.2	39.9
	TS_Pr (Mech. C(6m))[b]	24.9	37.1

[a]Figure S36, TS_Hyd, [b]Figure S29, TS_Pr

37. REFERENCES

1. Pangborn, A. B.; Giardello, M. A.; Grubbs, R. H.; Rosen, R. K.; Timmers, F. J., Safe and Convenient Procedure for Solvent Purification. *Organometallics* **1996**, *15*, 1518-1520.
2. Frisch, M. J. T., G. W.; Schlegel, H. B.; Scuseria, G. E.; Robb, M. A.; Cheeseman, J. R.; Scalmani, G.; Barone, V.; Mennucci, B.; Petersson, G. A.; Nakatsuji, H.; Caricato, M.; Li, X.; Hratchian, H. P.; Izmaylov, A. F.; Bloino, J.; Zheng, G.; Sonnenberg, J. L.; Hada, M.; Ehara, M.; Toyota, K.; Fukuda, R.; Hasegawa, J.; Ishida, M.; Nakajima, T.; Honda, Y.; Kitao, O.; Nakai, H.; Vreven, T.; Montgomery, J. A., Jr.; Peralta, J. E.; Ogliaro, F.; Bearpark, M.; Heyd, J. J.; Brothers, E.; Kudin, K. N.; Staroverov, V. N.; Kobayashi, R.; Normand, J.; Raghavachari, K.; Rendell, A.; Burant, J. C.; Iyengar, S. S.; Tomasi, J.; Cossi, M.; Rega, N.; Millam, J. M.; Klene, M.; Knox, J. E.; Cross, J. B.; Bakken, V.; Adamo, C.; Jaramillo, J.; Gomperts, R.; Stratmann, R. E.; Yazyev, O.; Austin, A. J.; Cammi, R.; Pomelli, C.; Ochterski, J. W.; Martin, R. L.; Morokuma, K.; Zakrzewski, V. G.; Voth, G. A.; Salvador, P.; Dannenberg, J. J.; Dapprich, S.; Daniels, A. D.; Farkas, O.; Foresman, J. B.; Ortiz, J. V.; Cioslowski, J.; Fox, D. J. , *Gaussian 09, rev. D.01* **2013**, Gaussian, Inc.: Wallingford, CT.
3. Becke, A. D., Density-functional exchange-energy approximation with correct asymptotic behavior. *Phys. Rev. A* **1988**, *38*, 3098-3100.
4. Lee, C.; Yang, W.; Parr, R. G., Development of the Colle-Salvetti correlation-energy formula into a functional of the electron density. *Phys. Rev. B* **1988**, *37*, 785-789.
5. Grimme, S.; Antony, J.; Ehrlich, S.; Krieg, H., A Consistent and Accurate Ab Initio Parametrization of Density Functional Dispersion Correction (DFT-D) for the 94 Elements H-Pu. *J. Chem. Phys.* **2010**, *132*, 1541041-15410419.
6. Perdew, J. P.; Burke, K.; Ernzerhof, M., Generalized Gradient Approximation Made Simple. *Phys. Rev. Lett.* **1996**, *77*, 3865-3868.
7. Adamo, C.; Barone, V., Toward reliable density functional methods without adjustable parameters: The PBE0 model. *J. Chem. Phys.* **1999**, *110*, 6158-6170.
8. Grimme, S.; Ehrlich, S.; Goerigk, L., Effect of the damping function in dispersion corrected density functional theory. *J. Comput. Chem.* **2011**, *32*, 1456-1465.
9. Chai, J.-D.; Head-Gordon, M., Long-range corrected hybrid density functionals with damped atom–atom dispersion corrections. *Phys. Chem. Chem. Phys.* **2008**, *10*, 6615-6620.
10. Tomasi, J.; Mennucci, B.; Cances, E., The IEF version of the PCM solvation method: an overview of a new method addressed to study molecular solutes at the QM ab initio level. *J. Mol. Struct.* **1999**, *464*, 211-226.
11. Tomasi, J.; Mennucci, B.; Cammi, R., Quantum Mechanical Continuum Solvation Models. *Chem. Rev.* **2005**, *105*, 2999-3094.
12. Tomasi, J.; Mennucci, B.; Cancès, E., *J. Mol. Struct.: THEOCHEM* **1999**, *464*, 211.
13. Tomasi, J.; Mennucci, B.; Cammi, R., Quantum mechanical continuum solvation models. *Chem Rev* **2005**, *105*, 2999-3093.
14. Krishnan, R.; Binkley, J. S.; Seeger, R.; Pople, J. A., Self-consistent molecular orbital methods. XX. A basis set for correlated wave functions. *J. Chem. Phys.* **1980**, *72*, 650-654.
15. Hay, P. J.; Wadt, W. R., Ab initio effective core potentials for molecular calculations. Potentials for K to Au including the outermost core orbitals. *J. Chem. Phys.* **1985**, *82*, 299-310.
16. Boys, S. F.; Bernardi, F., The calculation of small molecular interactions by the differences of separate total energies. Some procedures with reduced errors. *Mol. Phys.* **1970**, *19*, 553-566.
17. Simon, S.; Duran, M.; Dannenberg, J. J., How does basis set superposition error change the potential surfaces for hydrogen-bonded dimers? *J. Chem. Phys.* **1996**, *105*, 11024-11031.
18. Hopmann, K. H., How Accurate is DFT for Iridium-Mediated Chemistry? *Organometallics* **2016**, *35*, 3795-3807.
19. Hopmann, K. H., Quantum Chemical Studies of Asymmetric Reactions: Historical Aspects and Recent Examples. *Int. J. Quantum. Chem.* **2015**, *115*, 1232-1249.
